# Methods for non-proportional hazards in clinical trials: A systematic review

**DOI:** 10.1177/09622802241242325

**Published:** 2024-04-09

**Authors:** Maximilian Bardo, Cynthia Huber, Norbert Benda, Jonas Brugger, Tobias Fellinger, Vaidotas Galaune, Judith Heinz, Harald Heinzl, Andrew C Hooker, Florian Klinglmüller, Franz König, Tim Mathes, Martina Mittlböck, Martin Posch, Robin Ristl, Tim Friede

**Affiliations:** 1 Department of Medical Statistics, 27177 University Medical Center Göttingen, Göttingen, Germany; 2 9195 Federal Institute for Drugs and Medical Devices, Bonn, Germany; 3 Center for Medical Data Science, Section of Medical Statistics, 27271 Medical University of Vienna, Vienna, Austria; 4 Agentur für Gesundheit und Ernährungssicherheit (AGES), Vienna, Austria; 5 Department of Pharmacy, 8097 Uppsala University, Uppsala, Sweden; 6 Center for Medical Data Science, Section of Clinical Biometrics, 8097 Medical University of Vienna, Vienna, Austria; * Maximilian Bardo and Cynthia Huber contributed equally to this study.

**Keywords:** Cox model, log-rank test, survival analysis, right-censored observations, non-proportional hazards

## Abstract

For the analysis of time-to-event data, frequently used methods such as the log-rank test or the Cox proportional hazards model are based on the proportional hazards assumption, which is often debatable. Although a wide range of parametric and non-parametric methods for non-proportional hazards has been proposed, there is no consensus on the best approaches. To close this gap, we conducted a systematic literature search to identify statistical methods and software appropriate under non-proportional hazard. Our literature search identified 907 abstracts, out of which we included 211 articles, mostly methodological ones. Review articles and applications were less frequently identified. The articles discuss effect measures, effect estimation and regression approaches, hypothesis tests, and sample size calculation approaches, which are often tailored to specific non-proportional hazard situations. Using a unified notation, we provide an overview of methods available. Furthermore, we derive some guidance from the identified articles.

## 1. Introduction

**Figure 1.**
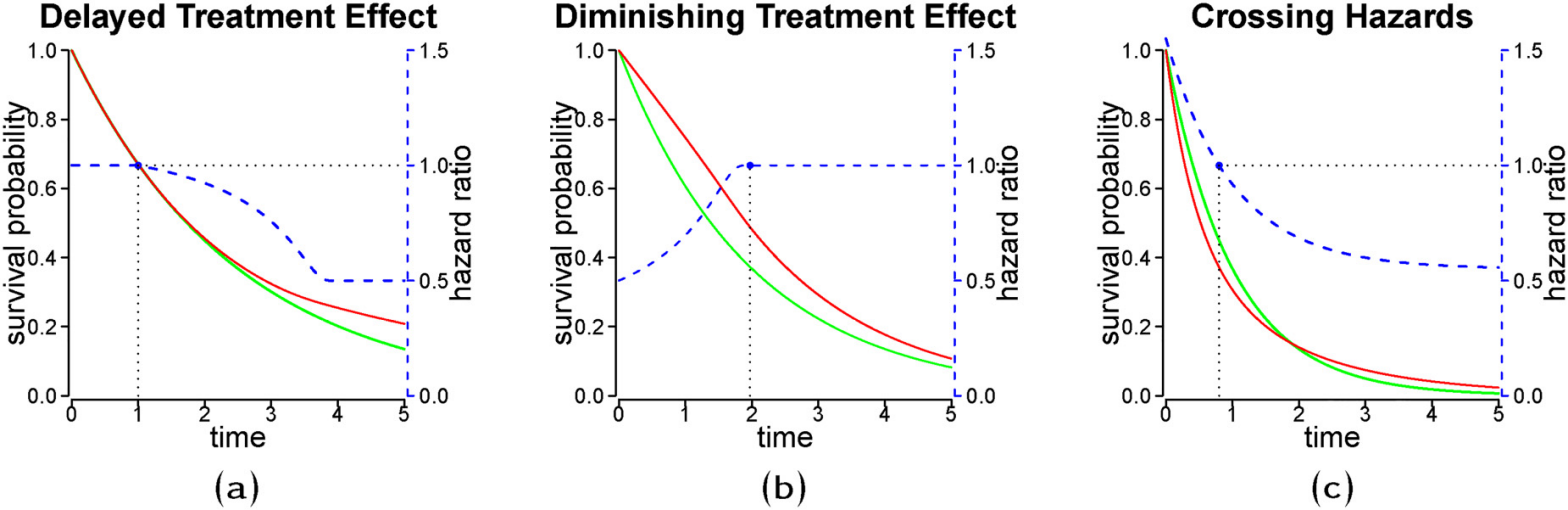
Stylized non-proportional hazard (NPH) treatment effect scenarios with hazard ratio (blue dashed line) and corresponding survival curves (red and green solid lines). The green line represents the reference group. The dotted line with black solid points refers to the time when the hazard ratio is equal to 1 . The displayed NPH scenarios are (a) Delayed effect; (b) diminishing effect; (c) crossing hazards.

In clinical studies with time-to-event outcomes, it is commonly assumed that the hazard functions of the treatment groups are proportional. However, several scenarios can lead to non-proportional hazards (NPHs). Figure 1(a) and (b) illustrate the hazard ratio (HR) of a delayed and a diminishing treatment effect, respectively. A delayed treatment effect for the experimental arm can also lead to crossing hazards (see Figure 1(c)) if the comparator is an active treatment with an immediate response as is often the case in trials concerning immuno-oncology drugs. Other scenarios of crossing hazards are experiments where the treatment effect is non-homogeneous across subgroups, i.e. if the treatment is harmful in a subgroup but beneficial in its complement.^1^ NPH can also occur in settings with long-term survivors in one treatment arm or if there is treatment switching to another arm after disease progression on the original arm.

Under proportional hazards (PHs), comparisons of HRs or cumulative hazard ratios (cHR) result in equivalent conclusions, whereas under NPH these results may vary substantially. Standard statistical tests for the comparison of time-to-event outcomes between groups such as the log-rank test or tests based on Cox regression models are not optimal for detecting relevant differences under NPH. Additionally, the HR estimate of the standard Cox regression model, a commonly used effect measure, is neither robust nor meaningful under NPH.^2^ In contrast to PH, the interpretation of estimates of a specific effect measure, such as the HR or the cHR, depend on the follow-up considered for evaluation in the presence of NPH.

Well-established methods for time-to-event data are available when the PH assumption holds. However, there is no consensus on best practices under NPH. Moreover, approaches to deal with NPH are not globally optimal but depend on the specific NPH scenario. A variety of parametric and non-parametric methods for treatment effect estimation and hypothesis testing in NPH settings have been proposed. We aim to identify statistical methods and, if available, the corresponding software that is suitable for NPH. In contrast to other overview articles that focus on specific disease areas (e.g., oncology^1^), NPH patterns (e.g., switching treatment^3^), or specific methods (e.g., statistical testing^4,5^), the scope of this literature review is broader and based on a systematic approach to identifying relevant literature. The remainder of this paper is organized as follows. In Section 2, we show the relevance of scenarios with NPH by investigating reconstructed data from a clinical trial. In Section 3, we describe the literature search, data extraction and summarize the quantitative results of the review. The identified approaches are presented in a common notation, which can be found in Section 4, where we focus on NPH for the treatment indicator. We categorize and discuss approaches to estimate and model treatment or covariate effects under NPH in Section 5. Testing and sample size calculation approaches under NPH are discussed in Section 6. We compare the flexibility of the proposed methods presented in Sections 5 and 6 on theoretical grounds and highlight results of conducted comparison studies if available. Finally, we summarize and discuss the findings in Section 7. The Appendix A provides more detailed information on the literature search and data extraction. The Online Supplement S provides more detailed information on the estimation and testing approaches identified as appropriate for NPH.

## 2. Motivation

**Figure 2.**
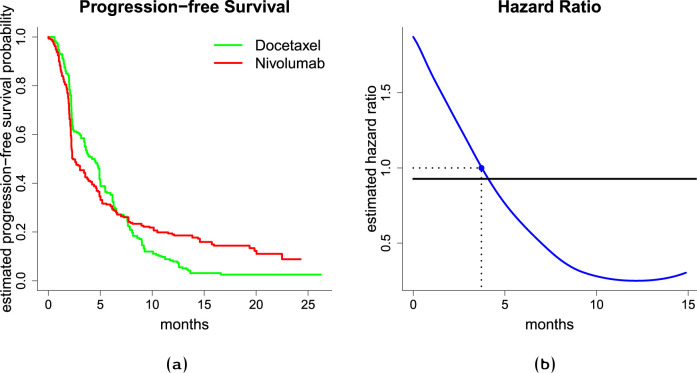
(a) Left-hand side figure shows Kaplan-Meier estimates of the survival function, (b) and right-hand side figure shows estimated hazard ratio. The solid blue line is an estimate of the time-varying hazard ratio obtained through smoothing the increments of the Nelson-Aalen estimate of the cumulative hazard function, the solid black line is an estimate from the Cox PH model, the dotted line with a blue solid point indicates a hazard ratio of 1, i.e. the time point, where the estimated hazard rates are equal.

Borghaei et al.^6^ report a phase 3 trial comparing the effect of nivolumab versus docetaxel in nonsquamous non-small lung cancer concerning overall survival. For illustration, we consider the study’s secondary endpoint, progression-free survival (PFS). Using the webplotdigitizer^7^ and the method described in Guyot et al.,^8^ we reconstructed the individual patient data by digitizing the Kaplan-Meier (KM) estimates of the survival functions. During follow-up, 238 of 292 patients in the nivolumab arm and 248 of 290 patients in the docetaxel arm either died or had lung cancer progression (reconstructed data). Figure 2(a) shows the re-estimated KM estimates of the PFS curve for the nivolumab and docetaxel group. The estimated KM curves are crossing, indicating a non-constant, crossing-hazards treatment effect. This is further investigated in Figure 2(b). The blue line shows the estimated (time-dependent) HR which is obtained by smoothing the increments of the cumulative hazard rates which are computed via the Nelson-Aalen estimator. Smoothening was done via kernel-based methods and global bandwidth as implemented in the R package muhaz. The estimated curve of the HR indicates an inferior treatment effect of nivolumab as compared to docetaxel early on. After approximately 4 months, however, the HR falls below one, favoring nivolumab. The crossing hazards result in crossing PFS curves, approximately two months after the HR crosses the threshold one, suggesting better performance of nivolumab. Borghaei et al.^6^ suspect that a delayed effect of the nivolumab treatment causes this effect which is typical for immunotherapy. The time-invariant estimate of the HR under the PH assumption is indicated by the solid black line in Figure 2(b). While the estimate of the constant HR is close to 1, indicating no treatment effect, the estimates of the time-varying HR and the KM curves suggest otherwise and provide additional insights. A log-rank test (on the reconstructed data) yields a test statistic of 0.6 , resulting in a p -value of 0.4 . This type of statistical analysis is typical for clinical studies. Jachno et al.^9^ review 66 trials with time-to-event outcomes. For analysis, the majority of papers reported KM curves ( 98 %) and the Cox PH model ( 97 %) and inference was based on the log-rank test in 88 % of the papers. Only 11 % of the reviewed papers in Jachno et al.^9^ reported either testing for or visual inspection of NPH. Moreover, at the stage of trial planning, only 11 % considered non-constant hazard rates or NPH, i.e. trial analysis is often restricted to PH methods. This could be problematic as the Cox PH model is misspecified under NPH. Consequently, its parameters are inappropriate to be interpreted as parameters of the time-to-event distribution, as it may not capture the nature of the treatment effect, as illustrated in the example above. Alternatively, the HR could be interpreted as a summary measure that quantifies the treatment effect in a single number, while the time-to-event distributions can be investigated using the KM estimates. However, the HR estimate under NPH depends on the censoring distribution and therefore lacks a clear interpretation.^10^ In addition, the log-rank test loses power under NPH, which could lead to medical advances not being detected as such. Ignoring the methodology for NPH and not testing for NPH makes it challenging to understand the impact of the PH assumption on the analysis of a specific trial. Dormuth et al.^4^ re-examined 18 clinical trials characterized by crossing survival curves and inconclusive log-rank tests. They discovered significant differences in survival outcomes in 9 of these trials when using testing procedures appropriate for NPH.

## 3. Systematic literature search and study selection

**Figure 3.**
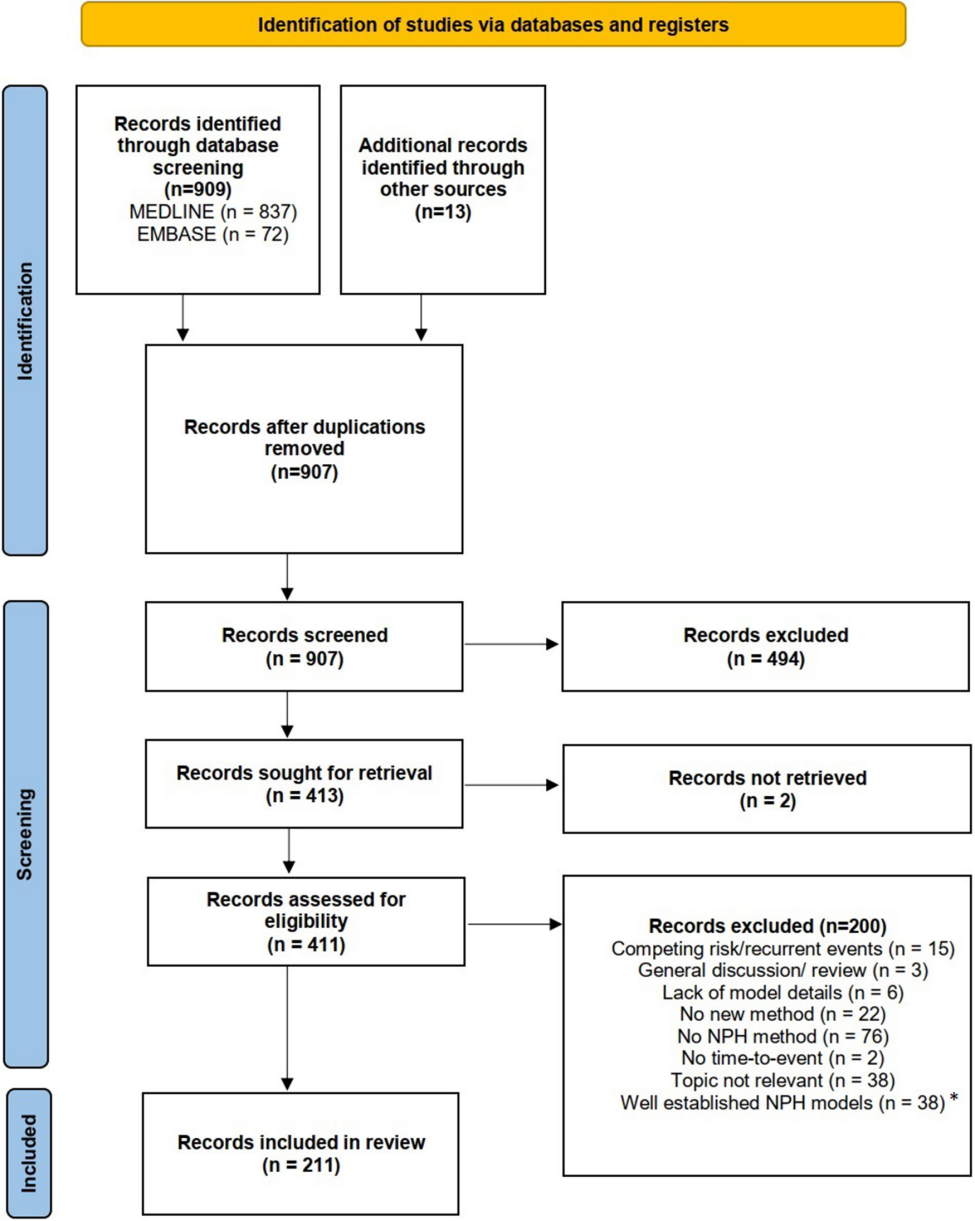
PRISMA 2020 flow diagram^11^—identified and included studies from the database searches (MEDLINE and EMBASE). *e.g. Stratified Cox PH model or use of time-dependent covariates in PH models as described in Klein and Moeschberger.^12^ ,Ch 9

We performed a comprehensive literature search using two electronic databases, MEDLINE and EMBASE, on March 15th, 2022. Details on the literature search and the data extraction are provided in Appendix A.1. In total 907 articles were identified, which were screened for eligibility. After the abstract screening and retrieval of full texts, a total of 411 articles were assessed for eligibility. In total, 200 articles (49%) were excluded. The most frequent reason for exclusion was that the articles neither developed nor applied any NPH method. The final analysis included 211 publications, see PRISMA flow chart in Figure 3. The complete list of included articles is available in Table S5 of the Online Supplement.

**Figure 4.**
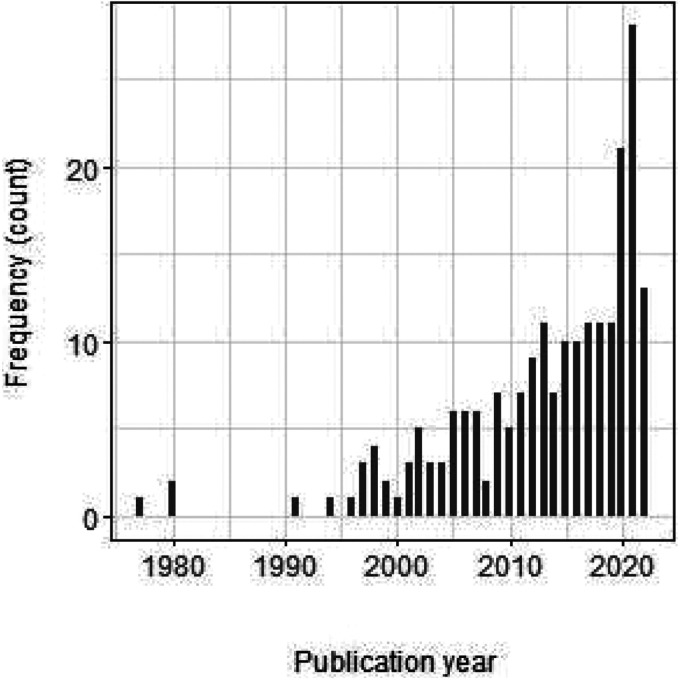
Publication year of the 211 included articles. Note that the number for 2022 is based on the articles published until 15th March 2022 and is therefore incomplete.

Figure 4 shows the publication years of the articles included. In our review more than 70% of the articles were published in 2010 or later and only a few before 2000. However, it has to be considered that the total number of published articles grew over the last years.^13^

**Table 1.** Absolute and relative frequencies of publications discussing a method class.

*Estimation approaches ( n =139)*	
Kaplan-Meier based estimation approaches	18 (12.9%)
Stratified Cox model	3 (2.2%)
Time-varying coefficients for the hazard rates	47 (33.8%)
Transformation models with time-covariate interaction	9 (6.5%)
Short- and long-term hazard ratio (HR)	9 (6.5%)
Joint models	3 (2.2%)
Frailty models	17 (12.2%)
Parametric models	38 (27.3%)
Machine learning approaches	11 (7.9%)
Other	8 (5.8%)
*Hypothesis testing approaches ( n =98)*	
Log-rank tests	63 (64.3%)
Kaplan-Meier-based tests	26 (26.5%)
Combination tests	20 (20.4%)
Other tests	12 (12.2%)

Note that publications may discuss methods belonging to multiple classes of method categories. Therefore the classes are not mutually exclusive.

The vast majority of articles ( > 80%) introduce statistical methods for NPH; reviews and applications were less frequent ( 10% ). Concerning the methods introduced in the identified articles we distinguished whether articles include methods for estimation of time-varying covariate/treatment effects and/or hypothesis testing. To further characterize the articles, we additionally introduce categories for articles focusing on estimation and/or testing methods. These categories are displayed in Table 1. Note that these categories are non-exclusive. The categories summarize the core contribution of the methods discussed in the corresponding articles. The category “Kaplan-Meier based estimation approaches” includes articles discussing for example approaches based on KM or Nelson-Aalen estimates, pseudo values and quantile regression. Articles grouped into “Time-varying coefficients for the hazard rates” discuss for example change-point approaches, splines and fractional polynomials. Further explanation of the categories is given below in Sections 5 and 6. The allocation of each paper to at least one of the categories according to Table 1 can be found in the Online Supplement, see Table S2. A categorization of estimation approaches into more detailed categories discussed in Section 5 can also be found in the supplement, see Table S3. In Tables S2 and S3, we attempted to identify the main NPH contribution for each paper, sometimes ignoring possible extensions that might have been mentioned. However, papers often cover multiple topics resulting in papers being classified into more than one category. Table S4 gives details regarding the null hypothesis considered in the proposed testing approaches. In total, 113 out of 211 (53%) articles identified in the review include estimation methods, 72 out of 211 (24%) involve hypothesis testing methods, and 26 out of 211 (13%) involve both hypothesis and estimation methods. Log-rank test approaches are the most frequent hypothesis test methods that we identified in our literature review (Table 1). Methods for trials including an interim analysis are considered in 12% of the articles.

The literature review identified articles covering different aspects of survival analysis in NPH settings. We identified articles proposing new test statistics for testing whether the survival is different in two treatment groups, as well as articles proposing new effect measures or regression models for quantifying the treatment effect in settings violating the common PH assumption.

In 72 out of 211 (34%) articles freely accessible software is provided. Another 13% of the articles provide the code for the methods upon request. Software was considered to be freely available code in form of e.g. R packages, code snippets given in the text, or freely accessible code (e.g. supplement of article or online repository). Additionally, publicly available code or code snippets for commercial software are also categorized as freely available although the software needed to run the code is not freely available. Code snippets published in the articles sometimes implement only specific features or are used to deepen the understanding of the methods. Moreover, the code snippets are usually intended to enable users to apply the methods proposed. Simulation studies are reported in 158 (75%) articles. This is more pronounced in articles considering testing procedures, where 86 out of 98 (88%) papers provide simulation studies. For 91 out of 139 (65%) papers that focus on estimation procedures simulation studies are provided.

## 4. Notation and summary effect measures

Before we proceed with describing the method categories according to Table 1, we introduce the notation that is used throughout this paper and the supplement. We also define the identified treatment effect measures.

### 4.1. Notation

The number of subjects included in a trial is denoted by N . Z is a treatment indicator with Z=0 indicating the control (placebo or comparator) and Z=1 the experimental treatment arm. The outcome of interest is the time to event T , whereas C denotes the censoring time. The event indicator is denoted by δ=I(T≤C) . The maximum follow-up time of the trial is set to $\tilde{t}$ and a specific time point during the follow-up time is denoted by $t^{*}$ . The distinct ordered event-times are indicated by $t_{\left(i\right)}$ , i.e. $0<t_{\left(1\right)}<t_{\left(2\right)}<\ldots$ where at each time $t_{\left(i\right)}$ at least one event occurred. Note that we define $t_{\left(0\right)}=0$ . Covariates or factors other than the treatment indicator are denoted by x . The regression coefficients for the treatment indicator and covariates are denoted γ and β , respectively. Note that we use γ(t) to denote a time-dependent treatment effect. Additionally, we will use indexing of γ if more than one parameter is required to specify the treatment effect. With $\lambda^{\left(Z\right)}(t)$ we denote the hazard rate of the treatment group Z with covariates x at time t , i.e. $\lim_{\epsilon \to 0}\;\frac{P(t\leq T<t+\epsilon |T\geq t,Z,x)}{\epsilon }\;$ , and with $\lambda_{0}(t)$ the baseline hazard rate respectively. The cumulative hazard rate $\Lambda^{\left(Z\right)}(t)$ equals $\int_{0}^{t}\lambda^{\left(Z\right)}(u)du$ . The survival function of treatment group Z with covariates x , P(T>t|Z,x) is indicated by $S^{\left(Z\right)}(t).$ Note that potential dependence of $\lambda^{\left(Z\right)},\Lambda^{\left(Z\right)}(t)$ , $S^{\left(Z\right)}$ on covariates x is suppressed in the notation. The at-risk indicator $Y_{i}(t)$ denotes whether patient i is uncensored and event-free at time t , $Y_{i}(t)=1$ , or not, $Y_{i}(t)=0$ . The number of patients at risk at time t is denoted by $Y(t)=\sum_{i}Y_{i}(t)$ . In general, we use ‘ (Z) ’ in the superscript to denote group-specific quantities.

Table S1 of the Supplement gives an overview of the used notation and the quantities defined in Section 4.2.

### 4.2. Effect measures

The treatment effect can be quantified, e.g., by the difference or ratio of the survival function at a chosen landmark time $t^{*}\leq \tilde{t}$ , i.e. $S^{\left(1\right)}(t^{*})-S^{\left(0\right)}(t^{*})$ or $S^{\left(1\right)}(t^{*})/S^{\left(0\right)}(t^{*})$ .^14^ Equivalent conclusions can be obtained by the cumulative HR ( cHR ) at time $t^{*}$ , $\text{cHR}(t^{*})=\Lambda^{\left(1\right)}(t^{*})/\Lambda^{\left(0\right)}(t^{*})$ . Alternatively, the $\tau^{\mathrm{th}}$ quantile of $T^{\left(0\right)}$ and $T^{\left(1\right)}$ may be compared, i.e. taking differences or ratios of $t_{\tau }^{\left(1\right)}={S^{\left(1\right)}}^{-1}(1-\tau )$ and $t_{\tau }^{\left(0\right)}={S^{\left(0\right)}}^{-1}(1-\tau )$ , where ${S^{\left(Z\right)}}^{-1}$ denotes the inverse of the survival function of treatment group Z .^14^ The above treatment effect measures are cumulative in that sense that they compare survival functions or cumulative hazard rates. Instantaneous differences between the treatment and the placebo group can be investigated by the instantaneous HR at $t^{*}$ , $\text{HR}(t^{*})=\lambda^{\left(1\right)}(t^{*})/\lambda^{\left(0\right)}(t^{*})$ . However, the $\text{HR}(t^{*})$ cannot necessarily be interpreted as the current (causal) effect of the treatment, as the population of survivors in the two treatment groups may differ for unmeasured characteristics or unadjusted covariates. Furthermore, conclusions based on a single time point $t^{*}$ (or quantile τ ) may not be meaningful, because analysis at a single time point (quantile) is not informative for the time points (quantiles) before or after the chosen $t^{*}$ ( τ ), where the treatment effect may be substantially different or even in the opposite direction. The dynamic of differences between survival time distributions over the course of time may be investigated by calculating the above effect measures over a suitable grid of time. Given that the hazard rate is often the pivot of modeling approaches, the HR(t) is a common choice. However, it may be difficult to assess the overall effectiveness of the treatment from an examination of the effect measure over time. This is particularly relevant for the HR(t) . For illustration assume a crossing hazards scenario as depicted by the blue dashed line in Figure 1(c). The trajectory of the HR(t) alone does not provide relevant information if and when the survival curves cross. For that, the trajectory of the baseline hazard rate in the same time period is also required. This illustrates that “extreme” values of the HR(t) in a certain time period do not tell whether these extreme differences in treated and untreated individuals result in relevant differences between survival time distributions. Hence, dynamic effect measures such as the HR(t) may be less useful to clinicians who need to make a treatment decision at t=0 and therefore need to know which treatment is superior with respect to some feature of the time-to-event distributions. For such binary decision-making, summary effect measures that summarize the treatment effect in a single number can be helpful.

A summary effect measure considers the entire survival curve (within the interval $[0,t^{*}],t^{*}\leq \tilde{t}$ ) and is thus usually of average nature. An attempt to summarize the treatment effect in a single number is the average HR $\text{aHR}(t^{*})=\;\int_{0}^{t^{*}}\frac{\lambda^{\left(1\right)}(t)}{\lambda (t)}dG(t)/\int_{0}^{t^{*}}\frac{\lambda^{\left(0\right)}(t)}{\lambda (t)}dG(t)$ , with $\lambda (t)=\lambda^{\left(1\right)}(t)+\;\lambda^{\left(0\right)}(t)$ and G(t) is a chosen weighting function, which might depend on the survival function and inverse probability of censoring weights.^15–17^ Note, that competing definitions of the aHR , different weighting functions and various estimation techniques exist. A related summary effect measure utilized in survival analysis is the concordance probability $P(T^{\left(0\right)}<T^{\left(1\right)})$ or odds of concordance $\frac{P(T^{\left(0\right)}<T^{\left(1\right)})}{1-P(T^{\left(0\right)}<T^{\left(1\right)})}$ .^16,18,19^

The restricted mean survival time (RMST) is the mean survival time within the time period [0, $\;t^{*}$ ], $t^{*}\leq \;\tilde{t}$ , i.e. $\text{RMST}(t^{*})=\;\int_{0}^{t^{*}}S(t)dt$ . An effect measure can again be constructed from the difference ${\text{RMST}}^{\left(1\right)}(t^{*})-\;{\text{RMST}}^{\left(0\right)}(t^{*})$ or the ratio, ${\text{RMST}}^{\left(1\right)}(t^{*})/{\text{RMST}}^{\left(0\right)}(t^{*})$ between the RMST of the treatment and the control group.^14^

In a NPH setting summary effect measures do not cover the dynamics of treatment efficacy and hence, do not necessarily deliver an adequate picture of the nature of the treatment effect over time. See Dehbi et al.^20^ for a discussion and potential remedy that relies on calculating more than one summary effect measure over varying time ranges.

For a comprehensive description of the survival distribution, the group-specific quantities $\lambda^{\left(Z\right)},\Lambda^{\left(Z\right)}$ or $S^{\left(Z\right)}$ are required. These can be estimated from statistical models or stratification of non-parametric estimation approaches by Z . Stratification reduces the need for assumptions, such as the PHs assumption, across different subgroups, as the estimation within each stratum relies solely on the information from that specific subgroup.

Effect measures unconditional of covariates x might require additional steps in estimation. As the covariates typically enter the survival function in a non-linear fashion, it will, in general, not be sufficient to plug E[X|Z] into $S^{\left(Z\right)}(t)$ to obtain $E[S^{\left(Z\right)}(t)|Z]$ , where the expectation is taken with respect to the covariates, due to Jensens’s inequality. The interested reader is referred to Keiding^21^ and Chapter 10 of Therneau and Grambsch.^22^ This also holds for the hazard rate $\lambda^{\left(Z\right)}(t)$ . Even if covariates would enter the hazard rate linearly, the expected covariate values given survival up to t of each treatment group would be necessary to obtain the hazard rate unconditional of covariates other than the treatment indicator. Further, note that the above-mentioned approaches would result in quantities that depend on the covariate distribution of the treatment group. To isolate the treatment effect, G-computation approaches could be used.

## 5. Estimation approaches for NPH treatment effects

This section describes the categories of identified estimation approaches for NPH treatment/covariate effects. The first column of Table 2 shows the main categories as introduced in Table 1. Some categories are divided into sub-categories given in the second column of Table 2. The third column of Table 2 provides a brief description of the methods. References to the Supplement are given in the first two columns of Table 2, where a more detailed overview can be found. In the corresponding section of the Supplement, references to Table S3 are given. In Table S3 of the Online Supplement, each paper that was considered in this literature review is allocated into one or more sub-categories according to Table 2, indicating the paper’s main contribution to address NPHs. Table S3 (column K) also provides information on whether the corresponding paper took a Bayesian estimation approach or not. The degree of detail and information given is hierarchical: Table 2 gives an overview, the referenced sections in the Online Supplement provide more detailed explanations including model formulas and a discussion of the literature, whereas Table S3 in combination with the respective papers (and the references therein) provide full information on the specific approaches to cope with NPH that we detected in the literature.

**Table 2.** Overview of methods suitable to estimate NPH effects.

Category	Sub-category		Assumptions on		
(#, ref.)^*^	(#, ref.)^*^	Description	$\lambda^{\left(0\right)}(t)$	HR(t)	Software
KM and NA based approaches (18, S.2.1)	Stratified KM, NA estimates (11, S.2.1)	The KM estimate of the survival function and the NA estimate of the cumulative hazard function are non-parametric estimators. Both do not impose any modeling assumption on the hazard rate. Hence, a stratified approach across treatment groups is suitable for any trajectory of HR(t) . The estimates of $S^{\left(Z\right)}(t)$ and $\Lambda^{\left(Z\right)}\left(t\right)$ might be processed to estimates of (summary) effect measures such as $\text{aHR}(t^{*})$ , $\text{cHR}(t^{*})$ or differences/ratios of the ${\text{RMST}}^{\left(Z\right)}(t^{*})$ .	None	None	survival, km.ci, survRM2, AHR (R), LIFETEST (SAS)
	Pseudo values (5, S.2.1.1)	Pseudo values are usually based on KM estimates of the survival function for the pooled data or summary measures that are computed thereof such as the $\text{RMST}(t^{*})$ . In an iterated leave-one-out fashion (jackknife) the difference for each individual between N times the whole sample estimate and N−1 times the estimate without the individuals’ contribution are calculated for a chosen landmark time $t^{*}$ . This difference represents a newly created metric variable for every observation and can be further investigated by linear or generalized linear regression models which include the treatment indicator as well as other covariates.	None	None	pseudo, prodlim (R), RMSTREG (SAS)
	Quantile regression (2, S.2.1.2)	In quantile regression, either the τth quantile or its logarithm are assumed to have a linear relationship with the covariates and the treatment indicator, e.g. $\ln\;\{t_{\tau }\}=x^{T}\beta (\tau )+Z\gamma (\tau )$ . Estimation procedures are either based on a generalized KM estimator or martingale-based estimation equations. Treatment efficacy at the τth survival quantile can be evaluated by γ(τ) . This process can be iterated over a grid of quantiles τ∈(0,1) , to get a complete picture of the potentially time-varying treatment effect over the course of time through γ(τ) .	None	None	quantreg (R), QUANT-LIFE (SAS)
Stratified Cox model (3, S.2.2)		The stratified Cox model relaxes the PH assumption by stratifying the baseline hazard rate along the treatment indicator, i.e. $\lambda^{\left(Z\right)}(t)=\exp\;\{x^{T}\beta \}\lambda_{0}^{\left(Z\right)}(t)$ . In the case of the non-parametric Breslow estimate of the baseline hazard rates, no structure is placed on the baseline hazards. The model is suitable for any trajectory of HR(t) . However, the PH assumption still holds for the remaining covariates. An estimate of the cHR(t) may be utilized to evaluate the treatment effect.	None	None	survival (R), PHREG (SAS)
Short- and long-term HR (9, S.2.6)		The models in this category differ from the models of the time-varying coefficient category in that sense that they do not formulate a non-constant HR(t) via a non-constant coefficient for the treatment effect γ(t) . Instead, a specific function is imposed on the hazard rate such that NPH arise. The approaches in this category differ from fully parametric approaches in that they have semi- or non-parametric components and do not fully specify the distribution of the survival time by assumption. This category mainly encompasses the Yang and Prentice model. The hazard function in the Yang and Prentice model is set to $\lambda^{\left(Z\right)}(t)=\;\frac{\exp(x^{T}\beta )\;}{\exp(-\gamma_{1}Z)\;S^{\left(0\right)}(t)+\exp(-\gamma_{2}Z){(1-S}^{\left(0\right)}(t)\;)}\lambda_{0}(t)$ . A time-dependent weight function puts all the weight to the first parameter $\gamma_{1}$ at the beginning. This weight is reduced over time and the weight for the second parameter $\gamma_{2}$ is analogously increased. Consequently, the HR is a time-weighted average of the short-term and the long-term hazard-ratio, $\exp\;\{\gamma_{1}\}$ and $\exp\;\{\gamma_{2}\}$ , respectively.	None to few	Strict	YPPE, YPBP (R)
Time-varying coefficients for the hazard rates (47, S.2.3)	Change point models (11, S.2.3.1)	The hazard rate equals $\lambda^{\left(Z\right)}(t)=\exp\;\{x^{T}\beta +Z\gamma (t)\}\lambda_{0}(t)$ . The time-dependent treatment coefficient is piecewise constant, $\gamma (t)=\gamma_{1}+\gamma_{2}I\{t\geq t^{*}\}$ , where I is the indicator function which is equal to one if the statement in the brackets is correct and 0 else. Note that the treatment coefficient is based on a time-covariate interaction and is not restricted to factors. The change point $t^{*}$ is pre-specified and more than one change point can be included, but black-box approaches also exist. Along with γ(t) , the HR(t) is also piecewise constant and hence rather restricted. The model might be estimated via the partial likelihood, which places no modeling assumption on the baseline hazard. Parametric choices for the baseline hazard are also possible.	Depends	Medium to strict	survival (R), PHREG (SAS)
	Smooth time-varying coefficients (26, S.2.3.2), e.g. fractional polynomials (6, S.2.3.3) or splines (12, S.2.3.4)	The hazard rate equals $\lambda^{\left(Z\right)}(t)=\exp\;\{x^{T}\beta +Z\gamma (t)\}\lambda_{0}(t)$ . The time-dependent treatment coefficient differs along with the chosen complexity and so do the trajectories of HR(t) that can be represented by the model. A general depiction is $\gamma (t)=\sum_{d}B_{d}(t)\gamma_{d}$ , where the basis functions $B_{d}(t)$ are smooth in t . Examples of basis function are, for example, fractional polynomials or B-Splines. The baseline hazard rate may be left unspecified by utilizing the partial likelihood or piecewise constant through the use of the Poisson GLM routine.	None to few	Few to strict	dynsurv, polspline, PenCoxFrail (R), ICPHREG (SAS)
	Weighted partial likelihood (12, S.2.3.5)	The weighted partial likelihood can be utilized to estimate a representative value for a time-varying treatment effect. This is usually the average HR $\text{aHR}(t^{*})$ . The weights have to be chosen and are not necessarily suitable for any trajectory of the HR(t) . Note that other approaches to estimate the $\text{aHR}(t^{*})$ exist, e.g. via KM.	None	None	coxphw (R), macro WCM (SAS)
	Aalen’s additive hazard model (4, S.2.3.6)	The hazard rate is equal to $\lambda^{\left(Z\right)}(t)=\lambda_{0}(t)+x^{T}\beta (t)+Z\gamma (t)$ . Estimation is based on the increments of the martingale process and is estimated via least squares at the distinct event times. In Aalen’s additive hazard model, there is no smoothness assumption on $\lambda_{0}(t)$ and γ(t) e.g. unlike when γ(t) is modeled via fractional polynomials or B-Splines.	None	None	timereg (R)
Frailty models (17, S.2.7)		Frailty or random effects models assume a heterogeneous population. Even if PH are assumed on the individual level, i.e. given the unobservable characteristics that make the population heterogeneous, the selection effects across the treatment and the placebo cause NPH on the population level in general, i.e. with unobservable characteristics marginalized out. In case of a diminishing treatment effect even crossing hazards are possible which might be caused by a catch-up process of the treatment group but not by a toxic treatment effect. This category also includes cure rate models that can be motivated by a discrete mixture distribution of a susceptible and non-susceptible population.	Depends	Strict	survival, coxme, frailtyEM, frailty-pack, PenCoxFrail (R), PHREG, NLMIXED (SAS)
Fully parametric approaches (38, S.2.8)	Piecewise exponential model (9, S.2.8.1)	The hazard rate is piecewise constant for that kind of model, i.e. there is a distinct parameter for each of non-overlapping time intervals. If the hazard rate is stratified by treatment the HR(t) is piecewise constant. Also termed piecewise constant hazard model.	Few to strict	Few to strict	pch, eha (R), PHREG (SAS)
	AFT & GAMLSS models (18, S.2.8.2)	AFT’s assume a specific distribution for T which typically leads to NPH, the Weibull distribution being a prominent exception. AFT’s formulate a treatment and covariate-specific location parameter, GAMLSS extend this to shape and scale parameters.	Medium to strict	Medium to strict	flexsurv, brms, spBayesSurv, mpr, gamlss.cen (R), LIFEREG (SAS)
	First hitting time models (4, S.2.8.3)	First hitting time models formulate a health process. The event occurs once the health process reaches a certain threshold (typically 0 ). Distributional assumptions on the health process determine the distribution of the survival time which typically has NPH. If the health process is assumed to be a Wiener process the distribution of the survival time is inverse Gaussian.	Medium to strict	Medium to strict	thregI (R)
	Other fully parametric approaches (10, S.2.8.4)	Other parametric approaches that do not fit in the former sub-categories are gathered in this category. This for example encompasses GLMs.	Medium to strict	Medium to strict	E.g. standard software for GLMs.
Transformation models with time-covariate interaction (9, S.2.4)		This category considers the Royston-Parmar and the conditional transformation model. Both approaches do not directly impose a model on the hazard rate, but formulate transformation functions as a spline function in (log) time. Time-varying treatment/covariates effects might be incorporated by spline by covariate interaction. The transformation functions are then brought into a parametric framework that determines their interpretation, e.g. as log cumulative hazard rates.	Few	Few	flexsurv (R), macro sas_stpm2 (SAS)
Joint models (3, S.2.5)		Joint models are typically fully parametric models, where measurements of, for example, drug concentrations over time per individual are modeled simultaneously with the time-to-event endpoint. The predictions of the other variable (concentration) from the model, or summary measures (exposure, area under the concentration-time curve) are used as a covariate in the time-to-event model.	Depends	Depends	JM (R and SAS), INLAjoint
Machine learning approaches (11, S.2.9)		We encountered a couple of machine learning approaches in the context of NPH scenarios such as trees (for example for finding the number and time points of a change point model) and forests, model averaging, k-nearest neighbor (in order to determine weights for weighted KM estimates), kernel smoothing based approaches and neural networks.	None to few	None to few	trtf, BART, mboost, ipred, randomForestSRC, Ranger (R)
Other approaches (8, S.2.10)		This is a collective category of methods that did not fit properly into one of the other categories. Among these methods are the rank preserving structural failure time model, concordance regression, the accelerated hazard model, the semi-parametric proportional likelihood ratio model, a Bayesian dependent Dirichlet process to model the time-to-event distribution, and others.	Depends	Depends	rpsftm (R)

More details on the categories can be found in the Online Supplement as indicated by the references given in brackets. ^*^ The assignment of papers to categories is not mutually exclusive. The symbol # refers to the number of papers in the corresponding category, ref. is a reference to the corresponding sections in the Supplement for the method description. KM: Kaplan-Meier; NPH: non-proportional hazard; NA: Nelson-Aalen; HR: hazard ratio; RMST: restricted mean survival time; cHR: cumulative hazard ratio; AFT: accelerated failure time; GAMLSS: generalized additive models for location scale and shape.

The fourth and fifth columns of Table 2 give a simplified impression of how flexible the corresponding approach is with respect to patterns of the hazard rate $\lambda^{\left(0\right)}(t)$ and time-varying NPH-patterns, respectively. Note that these statements focus on the treatment groups or, more generally, on the covariates where NPH are modeled. The assumptions on remaining covariate effects might be strict without mentioning this in Table 2. Hence, for many approaches, the flexibility of $\lambda^{\left(0\right)}(t)$ is determined by the flexibility of the baseline hazard rate $\lambda_{0}(t)$ . The statements on the flexibility of HR(t) can be understood as the flexibility of $\lambda^{\left(1\right)}(t)$ in contrast to $\lambda^{\left(0\right)}(t)$ and this determines the applicability of the method to arbitrary complex NPH scenarios. If there are no assumptions on the trajectory of $\lambda^{\left(0\right)}(t)$ and the HR(t) this is specified as “none” in Table 2. We define the assumptions to be “strict” if the corresponding methods tend to be restricted to monotonically increasing or decreasing trajectories for the HR(t) and to a single change in slope for $\lambda^{\left(0\right)}(t)$ . The statements “few” and “medium” indicate something in between. This categorization is closely related to a non- (none), semi- (few), and -parametric (“medium” to “strict”) handling of $\lambda^{\left(0\right)}(t)$ and HR(t) , where non-parametric approaches do not impose assumptions on the corresponding trajectories but parametric approaches tend to be limited in flexibility.

Note that the fourth and fifth columns of Table 2 is a statement about the flexibility of each method in accommodating varying scenarios of hazard rate functions (column 4) and time-varying HRs (column 5). Especially column 5 describes the capability of the methods to cope with varying NPH scenarios. However, this is not a statement as to whether estimates of the hazard rates and HR(t) are easy to obtain within the framework of the corresponding approach. The KM approach is an example where it is not possible to compute hazard rates and hence the time-varying HR(t) across two strata without further smoothing approaches.

The last column of Table 2 gives examples of software, including R-packages and SAS procedures, that are available for the groups of methodological approaches described.

Approaches with no or few assumptions on $\lambda^{\left(0\right)}(t)$ and HR(t) are suited for NPH scenarios that are more complex than those depicted in Figure 1(a) (delayed treatment effect), 1(b) (diminishing treatment effect), and 1(c) (crossing hazards). Such methods might also be utilized in the absence of knowledge of the trajectory of the HR and hazard rates or as a validity check of assumptions on its trajectory.

Stratification along the treatment indicator is a general tool to relax assumptions on the underlying estimation procedure. A stratified KM estimation approach along the treatment and placebo (comparator) group is suitable for any two possible trajectories of $S^{\left(0\right)}(t)$ and $S^{\left(1\right)}(t)$ or $\lambda^{\left(0\right)}(t)$ and HR(t) , respectively. If further (continuous) covariates are present the stratified Cox model might be utilized, where stratification along the treatment indicator is suitable for any trajectory of the HR(t) . Time-varying coefficients offer arbitrary flexibility, depending on the complexity one allows for γ(t) in $\lambda^{\left(Z\right)}(t)=\exp\;\{x^{T}\beta +Z\gamma (t)\}$ . High flexibility of the treatment effect can in particular be achieved if γ(t) is modeled via (penalized) splines or Aalen’s additive model. For the Royston-Parmar and the conditional transformation model, the basis functions in time can interact with the treatment indicator (or other covariates) in case of unknown or highly variable NPH scenarios. Machine learning procedures such as trees and forests as well as k-nearest neighbors or kernel approaches also offer high flexibility on $\lambda^{\left(0\right)}(t)$ and HR(t) .

Procedures with limited flexibility on HR(t) could be used if the trajectory is known/assumed, or if more flexible procedures suggest the appropriateness of more restrictive models. Less flexible models will typically be easier to analyze, as relatively few model parameters suffice to explain the trajectory of the treatment effect. Additionally, processing of model quantities, for example, hazard rates, to effect measures, such as the difference in RMST s or the aHR , might be more frequently analytically tractable than for the more flexible methods. Hence, results obtained from less flexible procedures might be easier to communicate. Further on, procedures with limited flexibility might avoid over-fitting the data which might result in more efficient estimates if the chosen procedure is well-suited for the data at hand.

The short- and long-term HR model introduced by Yang and Prentice^23^ is a suitable choice among the less flexible methods. The model moves the HR from an initial value HR(0) to HR(∞) in a monotone fashion.^24^ Hence, the model is suitable if PH or monotonically increasing/decreasing HRs are assumed. The Yang- and Prentice-model is in particular suited for delayed (Figure 1(a)), and diminishing treatment effects (Figure 1(b)) as well as crossing hazard (Figure 1(c)).^24^

For the change point model, a delayed or diminishing treatment effect as well as crossing hazards can be modeled by a single change point. More complex NPH scenarios might be accommodated by multiple change points, where Xu and Adak^2^ provide a tree-based method to determine the number and position of change points.

Furthermore, the accelerated failure time (AFT) model and its generalizations that also include covariates in the scale and shape parameters are a suitable choice. Note that the restrictions imposed on the HR(t) differ widely within the mentioned methods; for example, an AFT is way more restrictive than a regression model for location, scale, and shape parameters in general. Typically, effect parameters in a fully parametric model cannot be directly interpreted but most summary effect measures can be computed from the model.

The assumption of a homogeneous population or a homogeneous treatment effect can be dropped by utilizing frailty models. Both scenarios will typically lead to NPH on the population level, i.e. irrespective of individual, unobservable characteristics.^25,26^ Individual heterogeneity might even lead to crossing hazards on the population level if the treatment effect is beneficial but diminishing on the conditional level. This is caused by a catch-up process at later times of high-frail individuals from the treatment group who tend to survive longer due to the beneficial treatment.^27,p.252^ It also highlights that a population HR(t) above one not necessarily means that the treatment has a detrimental effect.

Empirical comparisons of NPH regression and estimation methods with simulated or real data without introducing new methodology have been rare in our literature review. Indeed, most papers provided simulation studies. However, giving recommendations on NPH methods based on the simulation studies is difficult for two reasons. Firstly, the simulation scenarios and procedures subject to investigation differ across the papers. Hence, an aggregated result is hard if not impossible to obtain from the existing simulation studies. Secondly, the simulation scenarios could have been chosen to demonstrate superiority of the new method.^28^

Based on the frequency of the methods in our literature review, time-varying coefficients for the hazard rates are the most typical choice for incorporating NPH covariate/treatment effects. Within that category, the $\text{aHR}(t^{*})$ via the weighted partial likelihood and time-varying coefficients via (cubic and B- ) splines, followed by change point models, are the most frequently studied methods. The second largest group is made up by parametric approaches. AFTs and generalized additive models for location scale and shape (GAMLSS) comprise the largest sub-group within the parametric approaches, followed by NPH approaches via the piecewise exponential model. See Table 2 for the frequencies of the sub-categories.

A review and simulation study on the $\text{aHR}(t^{*})$ can be found in Rauch et al.^17^ Rauch et al.^17^ investigated the performance of estimates of the $\text{aHR}(t^{*})$ either based on KM curves as discussed in Section S.2.1 or partial likelihood-based fitting procedures as discussed in Section S.2.3.5. Five different simulation scenarios, either with PH, strictly increasing or with strictly decreasing HR(t) , and administrative censoring as well as an administrative- combined with a random-censoring scheme were subject to investigation. The authors find few differences across the two estimation procedures when the shape parameter of the weighting function is 1 for the KM-based estimate. Inappropriately chosen weights might, however, inflate standard errors and introduce substantial bias. Different choices of weights might even result in opposite inference. Consequently, Rauch et al.^17^ suggest to carefully check the estimated survival curves to judge the plausibility of the estimates.

An investigation of weights in the context of weighted partial likelihood estimation of the $\text{aHR}(t^{*})$ can also be found in Schemper et al.^16^ The authors note that under PH, the $\text{aHR}(t^{*})$ leads to a loss of efficiency. However, for higher censoring rates and small deviations from PH, the loss in efficiency is reduced. Furthermore, the weighted partial likelihood has higher power than the Cox PH estimate under diminishing effects (if the weights emphasize early effects). In addition, the authors also highlight that the $\text{aHR}(t^{*})$ simplifies the analysis compared to models with time-varying coefficients for hazard rates.

From a theoretical point of view Rauch et al.^17^ also note that the partial likelihood estimate of the $\text{aHR}(t^{*})$ might consider further covariates what is not possible for KM-based estimates of the $\text{aHR}(t^{*})$ apart from a stratified analysis. The KM-based estimate of the $\text{aHR}(t^{*})$ , however, fulfills the independent increment property and so group-sequential and adaptive designs for tests relating to KM-based estimates of $\text{aHR}(t^{*})$ might be formulated.

A comparison of a (reduced rank) time-varying coefficient, gamma frailty, relaxed Burr, and a cure-rate model to real-world breast cancer data was conducted by Perperoglou et al.^29^ The authors emphasize interpretational differences across those models that might highlight different features of the data. In this sense, the time-varying coefficient model reveals the nature of the covariate effect, but it is not able to shed light on individual heterogeneity as the frailty model does. They conclude, that the specific research question should guide the model choice. Furthermore, the authors observe small differences in survival curves in their application and argue that the choice of how to tackle NPH is less important as long as the models are flexible enough for the data at hand.

## 6. Hypothesis tests for equality of survival curves

For the design and analysis of randomized controlled trials with time-to-event outcomes hypothesis tests for equality of survival curves from experimental and control treatment ( $H_{0}:S^{\left(0\right)}(t)=S^{\left(1\right)}(t)\;\forall \;t\geq 0$ ) are routinely applied. The equality of survival functions is also implied by the null hypothesis formulated in terms of the hazard rates $H_{0}:\lambda^{\left(0\right)}(t)=\lambda^{\left(1\right)}(t)\;\forall \;t\geq 0$ or in terms of the HR $H_{0}:\;\text{HR}(t)=1$ ∀t≥0 . Note, that conclusions for times beyond the maximum follow-up time $\tilde{t}$ should be avoided.

Moreover, null hypotheses based on summary effect measures, e.g., $H_{0}:\;\Delta \left(t^{*}\right)={\text{RMST}}^{\left(1\right)}(t^{*})-{\text{RMST}}^{\left(0\right)}(t^{*})=\;0$ or $H_{0}:\;\text{aHR}(t^{*})=1$ can also be considered as valid tests of the equality hypothesis, as rejection of a more specific null hypothesis implies rejection of equality. A “stronger” null hypothesis that survival in the experimental treatment is less or equal to the survival in the control arm, $H_{0}:S^{\left(1\right)}(t)\leq S^{\left(0\right)}(t)\;\forall \;t\geq 0$ , is also of interest. However, the implication of $S^{\left(1\right)}(t)\leq S^{\left(0\right)}(t)\;\forall \;t\geq 0\Rightarrow \lambda^{\left(1\right)}(t)\geq \lambda^{\left(0\right)}(t)$ only holds under the PH assumption but is not generally true under NPH.^30^

Table S4 gives an overview of the used null hypothesis in the articles focusing on hypothesis tests. We classified whether the null hypothesis was defined as equality of survival, less or equal survival in the experimental arm, or whether it was an average-based hypothesis.

Under the assumption of PH the log-rank test is the standard procedure. However, if the PH assumption does not hold, power is reduced and the alternative hypothesis cannot necessarily be interpreted as treatment benefit. Moreover, rejecting the null hypothesis $H_{0}:S^{\left(0\right)}(t)=S^{\left(1\right)}(t)\;\forall t\geq 0$ in settings with NPH means that there is a non-zero treatment effect at least in some time interval.

For situations in which the PH assumption may not hold, alternative hypothesis tests and sample size calculation approaches have been proposed, which we identified in the literature review.

In our literature review, we identified three categories of hypothesis tests for the above-mentioned null hypotheses in NPH scenarios: Log-rank tests, KM-based tests, and combination tests. Table 3 gives an overview of these different types of tests and provides examples for software, e.g. R-packages or SAS procedures. Additionally, Table S2 shows in which categories the identified articles fall. Table S4 provides an overview of whether the identified articles consider approaches for sample size calculation.

**Table 3.** Overview of hypothesis tests for NPH.

Category			Examples of
(Reference^a^)	Sub-category	Description	software
Log-rank tests (S.3.1)	Log-rank test	Most widely used statistical test to compare the overall survival of two groups. The null hypothesis is $H_{0}:\lambda^{\left(0\right)}(t)=\lambda^{\left(1\right)}(t),t\geq 0$ in case of a two-sided alternative or $H_{0}:\lambda^{\left(0\right)}(t)\leq \lambda^{\left(1\right)}(t),t\geq 0$ in case of a one-sided test.	survival, nph (R), LIFETEST (SAS)
	Weighted log-rank test	Augments the log-rank test with weights $w(t_{\left(i\right)})$ to emphasize observations based on their point in time. Null- and alternative hypotheses are identical to the standard log-rank test if the weights are positive.	survival, nph (R), LIFETEST (SAS)
	Modestly weighted log-rank test	Robust variation of the weighted Log-rank test. The weights are chosen such that in case of locally favorable hazards alone the test will not wrongly infer superiority of the treatment group. Null- and alternative hypotheses are identical to the standard log-rank test.	nphRCT (R)
KM-based tests (S.3.2)	Weighted KM tests	Tests based on the weighted sum of the differences of the KM estimates of the survival curves.	
	RMST	Test for differences in restricted mean survival up to $t^{*}$ based on the empirical survival curves. $H_{0}:\Delta (t^{*})={\text{RMST}}^{\left(1\right)}(t^{*})-{\text{RMST}}^{\left(0\right)}(t^{*})=0$ in case of a two-sided test and $H_{0}:\Delta (t^{*})={\text{RMST}}^{\left(1\right)}(t^{*})-{\text{RMST}}^{\left(0\right)}(t^{*})\leq 0$ in case of a one-sided alternative. Effect sizes are computed using Wald statistics. A maximum test statistic can be obtained from a set of potential time points $t^{*}\in \{t_{1},\ldots ,t_{K}\}$ .	nph, survRM2, survRM2adap (R), LIFETEST, RMSTREG (SAS)
	Average HR test statistic	Test for differences in average HRs of two groups. Null hypotheses for two- and one-sided alternatives are defined as $H_{0}:\text{aHR}=1$ and $H_{0}:\text{aHR}\geq 1$ , respectively.	nph (R)
	Window mean survival time	Test for differences in mean survival time of two groups between two time points $t_{1}^{*}$ and $t_{2}^{*}$ . Null- and alternative hypotheses are analogous to the test for difference in RMST.	
Combination tests (S.3.3)	Max combo test	Maximum test of K differently weighted log-rank test statistics. The p-value of the largest test statistic is obtained from the joint multivariate normal distribution of individual test statistics. Null- and alternative hypotheses are identical to the standard log-rank test.	nph, maxcombo (R)
	Cox test and RMST difference	Combination of Cox likelihood ratio test in a Cox regression model and test for difference in RMST. The global null hypothesis is the equivalence of survival curves in a two-group setting.	
	Multiple direction test	Combination of weighted log-rank statistics targeting a comprehensive range of alternatives. Critical values are calculated using permutation approaches. The null hypothesis is the equivalence of two survival distributions.	mdir.logrank
Other tests (S.3.4)	Test by Gorfine et al	Test for equivalence of survival functions of K groups based on sample size partitions. Under the null hypothesis, survival curves of all K groups are identical.	KONPsurv (R)
	Modification of the Kolmogorov Smirnov test	Generalization of the Kolmogorov Smirnov test for use with right-censored data. Null hypothesis is the equivalency of two survival curves.	
	Test by Sooriyarachchi and Whitehead	Test for differences in survival curves based on the log odds ratio of the probability of surviving past a given time point $t^{*}$ . The null hypothesis is the equivalency of two survival curves.	

More details on the categories can be found in the Online Supplement as indicated by the references given in brackets. KM: Kaplan-Meier; RMST: restricted mean survival time; HR: hazard ratio. ^a^ See corresponding sections in Appendix for the method description.

With prior knowledge of the NPH pattern, weighted log-rank tests can consider certain time periods to be more relevant than others. KM-based tests are especially appealing to practitioners due to their intuitive interpretation. Combination tests select a test statistic from a small set of prespecified test statistics based on the data and are therefore useful without any prior knowledge regarding the NPH pattern. Our literature review also identified articles reviewing and comparing hypothesis testing methods under different NPH settings. For instance, Yang^31^ applies different tests including weighted log-rank tests, combination tests, and Wald tests based on estimators of the average HR or RMST to different randomized controlled trials to illustrate the virtually ignorable loss of power for reasonably PH situations and emphasizes the substantial gain of power using these approaches in contrast to the standard log-rank test in NPH situations. Many new tests are tailored to specific NPH situations, see Section S.3. Therefore, Yang^31^ favors the adaptively weighted log-rank test due to its overall trade-off.

In the comparison study of Dormuth et al.,^4^ in which data sets of oncology trials were reconstructed, the proposed log-rank permutation test of Ditzhaus and Friedrich^32^ detected most differences between treatment groups. These results align with those of other articles investigating omnibus tests, e.g. Gorfine et al.^33^ and Royston and Parmar.^5^ If there is uncertainty regarding the underlying survival time distributions, a more recent article by Dormuth et al.^34^ recommends the use of omnibus tests for comparisons between groups.

Li et al.,^35^ Callegaro and Spiessens,^36^ Royston and Parmar^5^ and Lin et al.^37^ perform simulation studies for comparing different test statistics for settings with NPH. Li et al.^35^ applied amongst others tests of the log-rank test family, KM-based tests, and combination tests to situations of crossing survival curves at early, middle, and late times. They concluded that the adaptive Neyman’s smooth test^38^ and the two-stage procedure of Qiu and Sheng^39^ have higher power in the considered NPH settings, provide an acceptable power under PH, and their type I error rate is close to the nominal level. Therefore, Li et al.^35^ recommend the use of these tests as they are “the most stable and feasible approaches for a variety of situations and censoring rates.”

The comparison study of Callegaro and Spiessens^36^ involves, among others, the weighted log-rank test with weights of the Fleming-Harrington weight family, max combo tests, and the likelihood ratio test for testing the treatment effect in a Cox model with time-varying coefficients. Callegaro and Spiessens^36^ consider the latter to be often more powerful than the weighted log-rank tests.

Lin et al.^37^ compare tests of the class of weighted log-rank, KM, and combination tests. The comparison study did not identify a single test outperforming the others in all considered scenarios; e.g. delayed treatment onset, diminishing effects, crossing hazards, PHs, and delayed effects with converging tails. The comparison study suggests the max combo test as a robust test across different NPH patterns without prior knowledge of the pattern. The review of Mukhopadhyay et al.^40^ compared the log-rank test to the MaxCombo test in immo-oncology trials identified through a systematic literature review. The authors concluded that the MaxCombo test is a “pragmatic alternative” to the log-rank test assuming NPH. The simulations of Royston and Parmar^5^ suggest that the modified versatile weighted log-rank test, an unpublished modification of the versatile weighted log-rank test^41^ with Stata code available on request from Royston, performs best in terms of power under NPH (early, late or near PH treatment effect) without the preconceived type of treatment effect.

In the last 20 years, there have been constant publications on log-rank tests. Research on combination tests, KM-based tests, or other approaches has been comparatively rare. In the last 3 years, however, more research on these testing categories including permutation approaches, e.g. Ditzhaus and Friedrich^32^ and Ditzhaus et al.,^42^ was conducted.

## 7. Conclusions and discussion

We conducted a systematic literature review of effect estimation and testing methods that are able to cope with NPH in time-to-event analysis. Review articles focusing on different methods for NPH have been published previously. These reviews mostly focus either on a quantitative comparison for specific NPH scenarios,^35^ or a specific method class,^17^ or on a qualitative overview of available methods for specific NPH situations or disease areas, e.g. oncology.^1^ We conducted a systematic literature search for methodological approaches for any NPH scenario, any model class, and not restricted to a specific disease area. Therefore, our review gives a comprehensive overview of the methods proposed and applicable to NPH settings.

In total, our literature review includes 211 articles for final analysis. Of those articles, 113 focus on effect estimation, e.g. regression methods, 72 on testing, and 26 articles on both. In the effects estimation and testing literature, we identified categories to group articles according to their approach to the NPH situation. With respect to effect estimation, the categories are KM-based estimation approaches, stratified Cox model, time-varying coefficients for the hazard rates, transformation models with time-covariate interaction, short- and long-term HR, joint models, frailty models, parametric models, machine learning approaches and others. With respect to testing, the categories are log-rank tests, KM tests, combination tests, and other tests. We have also broken down some of the categories into smaller sub-categories and assigned each paper to at least one of them. An overview of the categories and subcategories is given in Tables 2 and 3, for estimation and testing approaches respectively. The tables and Sections 5 and 6 provide brief explanations of the categories. For a more detailed discussion including references to the original articles proposing specific methods, we refer to the Supplement S. The most common approaches to tackle NPH for effect estimation are time-varying coefficients for the hazard rates (47 papers), and parametric approaches that assume a distribution for the survival time (38 papers), such as GAMLSS models. The most common testing approach for NPH are variations of the log-rank test (63 papers). We extracted and documented the software (R and SAS) utilized in the papers under review. In addition, well-known software for the individual testing and estimation categories was added by the group of authors. For a more complete overview of available R packages for time-to-event analysis see the CRAN Task View homepage for Survival Analysis.^43^

For the literature review, we excluded standard methods such as the stratified Cox model, unless the baseline hazards were stratified by the treatment indicator. Consequently, our review may have missed certain innovative proposals in this area. In addition, we have excluded methods that utilize internal^44,p.198^ time-varying covariates which might lead to NPH over time, e.g. PKPD Models. Further, our search terms focused on terms related to NPH, which may not be a common term in other areas utilizing these methods. For review articles considered in this review, we manually added all investigated methods to the list of articles. Nevertheless, some of those may have been later discarded due to our in- or exclusion criteria, see Figure 3. Consequently, some of the considered review articles may investigate methods which have not been discussed in this review.

A broad range of different methods is available for both treatment effect estimation and hypothesis testing. However, there is no consensus on the best approaches under NPH. Most papers reported simulation studies (158 of 211 papers). Nevertheless, the NPH scenarios and the methods under comparison differ making it difficult to aggregate and compare results across evaluations. Moreover, the NPH scenario and the competitors to newly introduced methodology might have been chosen to demonstrate superiority of the newcomer.^28^ Only a few review articles comparing different methods through simulation studies (considered to be objective) have been identified by our review. In particular for effect estimation methodology, independent comparison studies including neutral comparison studies covering different NPH scenarios and a broad range of methods are not available. Review articles of testing procedures cover a broad range of different NPH settings and provide guidance for the choice of the test, which, however, can be different from one comparison study to another. These reviews offer some guidance on, for example, the permutation test by Ditzhaus and Friedrich,^32^ and the adaptively weighted log-rank test^24^ for specific NPH scenarios. Due to the hypothesis tests examined not being consistent across the comparison studies, it is difficult to make a general recommendation for the use of a specific hypothesis test.

The choice of an estimation method could be based on theoretical considerations. In the absence of strong prior knowledge of the treatment/covariate effect, time-dependent treatment coefficients for the hazard rates could be flexibly modeled via a treatment spline interaction, where the corresponding basis functions are constructed on time. In the case of strong prior knowledge, more restrictive models might be preferred, such as a (single) change point model for a delayed treatment effect (Figure 1(a)).

Moreover, different summary effect measures have been proposed which offer an alternative to the HR. The constant HR estimated by a Cox PH model is commonly used for time-to-event analysis but might be misleading under NPH as the HR is time-dependent in this case. Alternatives involve, for example, the average HR and the ratio of RMSTs. These depend on the choice of the pre-specified time interval which is restricted by the maximum follow-up time. Additionally, its usefulness depends on the pattern of the treatment effect. For instance, the difference of RMST between treatment groups is not useful for delayed treatment effects.^45^ Summary effect measures can be calculated based on KM curves. For more complex data, e.g. multiple continuous covariates, other methods presented in Section 5 can be used to model the survival curves. Depending on the choice of the estimation approach it might be difficult if not impossible to obtain specific summary effect measures, however. Dynamic, i.e. time-varying, effect measures could be used instead and could help to communicate how survival patterns are affected by the treatment over time. However, dynamic effect measures are less appropriate as a primary basis for binary decisions such as marketing authorizations. Nevertheless, they could be used to support a decision following a gatekeeping hypothesis test on any difference, hence disentangling the hypothesis test and estimation. As a drawback, such a decision procedure could not be clearly defined in advance. In contrast to this, single summarizing measures such as RMST difference can be used for both, hypothesis tests and estimation, and lead to an unambiguous binary decision procedure but require an upfront agreement on the most relevant measure.

We identified a variety of NPH approaches for both, effect estimation and testing procedures. Although a variety of NPH methods are available, they are still rarely applied. Statistical practice needs to change by adopting the NPHs approaches summarized in this paper. Adhering to invalid assumptions, i.e. PHs, might lead to less reliable conclusions than choosing a non-optimal NPH approach for the data at hand as illustrated in Section 2. To fill the gap in comparisons of the methods for NPH, our further assessment will explore the advantages and disadvantages under a wide range of NPH assumptions of a selection of the identified methods, see Klinglmüller et al.^46^

## Supplemental Material

sj-pdf-1-smm-10.1177_09622802241242325 - Supplemental material for Methods for non-proportional hazards in clinical trials: A systematic review Supplemental material, sj-pdf-1-smm-10.1177_09622802241242325 for Methods for non-proportional hazards in clinical trials: A systematic review by Maximilian Bardo, Cynthia Huber, Norbert Benda, Jonas Brugger, Tobias Fellinger, Vaidotas Galaune, Judith Heinz, Harald Heinzl, Andrew C Hooker, Florian Klinglmüller, Franz König, Tim Mathes, Martina Mittlböck, Martin Posch, Robin Ristl and Tim Friede in Statistical Methods in Medical Research

## Appendix

### A.1 Literature search and data extraction

#### A.1.1 Systematic literature search and study selection

We performed a comprehensive literature search using the electronic databases MEDLINE and EMBASE to identify relevant publications. Pubmed was used to search the database MEDLINE. EMBASE searches in both databases MEDLINE and EMBASE. However, we excluded the database MEDLINE for our search in EMBASE. Table 5 provides details of the literature search. The search includes two parts. One part of the search includes topic-related search terms such as “non-proportional hazards” in various spellings (part one of the search strategy (main search in MEDLINE and EMBASE), rows 1 to 9 of 5). In addition, in a second part of the search we used broader pre-specified topic-related terms (e.g. “crossing hazard*”, “delayed effect”, “treatment switch”). The part of the search with the pre-specified related search terms has been restricted to statistical journals using the Web of Science category ‘Statistics & Probability’ (part two of the search strategy (additional search in MEDLINE), row 10 to 19 of Table 3). Reference lists of included publications and relevant reviews were checked manually to identify any additional relevant articles that were not captured by the search.

We included methodological research articles, reviews, and clinical investigations with time-to-event endpoints where methods for non-proportional hazards were applied. More specifically, the inclusion and exclusion criteria specified in Table 4 have been used.

**Table 4.** Inclusion and exclusion criteria.

Inclusion criteria	Exclusion criteria
Methodological publications introducing methods for time-to-event analyses of clinical trials with non-proportional hazard (NPH) assumptions	Investigations assessing NPH assumptions without introducing new NPH methods in the analysis
Applications of new NPH methods in clinical trials with time-to-event endpoints	Applications of conventional methods for NPH such as stratified Cox regressions or the use of time-dependent covariates in proportional hazards models^12^
	Methods for competing risk or recurrent event analyses

#### A.1.2 Data extraction

Two reviewers screened the search results for relevant articles independently. The reviewers screened titles and abstracts and excluded papers that clearly did not meet the inclusion criteria. During the abstract screening, an article was included for the full-text screening, if at least one of the two reviewers included the abstract. For all selected abstracts, we obtained the full texts and two reviewers reviewed these according to the pre-specified inclusion criteria (e.g. statistical methods, simulations, time-to-event endpoints, non-proportionality assumption). From the full text, data on basic characteristics of the investigation, on proposed methods, and, if applicable, on simulation studies conducted were collected by two independent reviewers. A third reviewer resolved all disagreements on the inclusion of the full texts and on the extracted data. Characteristics of the proposed methods include whether the methods use frequentist or Bayesian, parametric or non-parametric approaches and whether the methods adjust for covariates. We distinguished between articles including methods for treatment effect estimation, hypothesis testing and manuscripts covering both. We also extracted the availability of a software for the proposed method and differentiated whether the software was freely available, e.g. Github repository for code, R package or Code snippets in text, available, e.g. available upon request from the author, or not available or mentioned in the text. Additionally, the proposed methods were classified into one of the predefined categories:

- Log-rank approaches, e.g. weighted log-rank test
- Kaplan-Meier curve-based approaches; e.g. weighted Kaplan-Meier and RMST
- Combination approaches; e.g. Breslow test, Lee’s combo test, or MaxCombo test
- Regression model (parametric/semiparametric/nonparametric)
- Other; if the previously mentioned classes do not fit the method.

#### A.1.3 Quantitative and qualitative analysis

Characteristics of articles and proposed methods are described by using descriptive analyses such as bar charts. The collected categorical variables are summarized by providing absolute and relative frequencies. In order to enable an overview of the methods proposed in the identified articles, we differentiated between methods for treatment effect estimation (see Section 5) and hypothesis test for the comparison of survival between treatment groups (see Section 6). We additionally introduced new categories for publications focusing on treatment effect estimations via e.g. regression approaches. The categories are Kaplan-Meier for effect estimation (Section S.2.1), stratified Cox (Section S.2.2), time-varying coefficients for the hazard rates (Section S.2.3), transformation models with time-covariate interaction (Section S.2.4), joint models (Section S.2.5), short- and long-term HR (Section S.2.6), frailty models (Section S.2.7), parametric regression models (Section S.2.8), machine learning approaches (Section S.2.9) and other modeling approaches (Section S.2.10). The main characteristics of these categories are introduced in a common notation and examples for the categories are provided and described in more detail (Section 5). All statistical analyses (tables and figures) are performed in R 4.1.2 (R Core Team, 2021).

#### A.1.4 Data extraction guideline

For the data extraction each reviewer was provided with the following instructions including the in-and exclusion criteria:

***CONFIRMS - Literature review*** AIM: Methods and statistical software for statistical analysis and reporting of clinical trials with time-to-event endpoints with non-proportional hazards INCLUSION:

- Methodological publications introducing methods for time-to-event analyses of clinical trials with non-proportional hazard (NPH) assumptions
- Applications of new NPH methods in clinical trials with time-to-event endpoints

EXCLUSION:

- Investigations assessing NPH assumptions without introducing new NPH methods in the analysis
- Applications of conventional methods for NPH such as stratified Cox regressions or the use of time-dependent covariates in proportional hazard models
- Methods for competing risk or recurrent event analyses

SCREENING–second step—full texts AND DATA EXTRACTION: Please fill in the columns L to O for all full texts

1. Type of paper (methods/applied/review)
2. For the inclusion/exclusion of the full text, please fill the columns L to N in the excel sheet:
  — Inclusion (yes/no)
  — Please provide the reason for non-inclusion (if “other”, please specify)

If the full text is included, please fill in the columns P to AH

1. Simulation study conducted (yes/no)
2. Software available (not available/available/freely available)
  — Not available/not mentioned: No mention of code availability made in the text
  — available: Availability mentioned, but code not freely available, e.g. “Data available upon request from the authors”
  — freely available: Code freely available (as stated in the paper, without background checks), e.g. Github repository for code, R package, Code snippets in the text,….

- If software freely available, software specification (text); e.g. name of R package, link to Github repository, etc.
- Provides program for sample size calculation (yes/no)
- Provides program for analysis (yes/no)

1. The following items capture information on the new method or methods. If multiple new methods are described, please classify each of them within this row. If the predefined categories (log-rank, Kaplan Meier,…) do not fit all of new methods well, please use the column “Specification of other class” and give some details on the new method or methods.
  — Classification of the method as separate items with dropdown (yes/no)
  — Log-rank approaches, e.g. weighted log-rank test
  — Kaplan-Meier curve-based approaches; e.g. weighted Kaplan-Meier and RMST
  — Combination approaches; e.g. Breslow test, Lee’s combo test, or MaxCombo test
  — Regression model
    * If regression model, parametric/semiparametric/nonparametric (dropdown)
  — Other class; if the above-mentioned classes do not fit the method
2. If other, specification of other class for the new NPH method or methods (text, if several new methods proposed, comment on them individually in this box)
3. Bayesian or frequentist approach of the method (dropdown)
4. Covariate adjustment included in the method (not discussed/stratification for factors/adjustment for covariates)
5. Methods for hypothesis testing (yes/no)
6. Methods for estimation (yes/no)
7. Interim analysis (yes/no)
8. Interesting case studies to inform simulation studies (yes/no); e.g. only RCTs and the data should be presented in sufficient detail to inform the simulation study, e.g. Kaplan-Meier curve with number of patients at risk
9. Comments (text); name of the methods and optionally further details

#### A.1.5 Search term for the literature review

**Table 5.** Search terms for the literature review.

	Search terms	
1	“non-proportional hazard” OR “non-proportional hazards” OR “nonproportional hazard” OR “nonproportional hazards” OR “non proportional hazard” OR “non proportional hazards”	Main search in MEDLINE and EMBASE without any journal restrictions
2	non-proportionality OR nonproportionality OR “non proportionality”	
3	(NPH OR NPHs OR “non PH” OR “non PHs” OR non-PH OR non-PHs) AND (assumption OR assumptions)	
4	“proportional hazard” OR “proportional hazards” OR “hazard assumption” OR “hazard assumptions”	
5	violation OR violations OR violated OR violat*	
6	#4 AND #5	
7	#1 OR #2 OR #3 OR #6	
8	“delayed treatment effect” OR “delayed treatment effects”	
9	#7 OR #8	
10	crossing AND (hazard OR hazards)	Additional search with broader search terms in journals of the category “Statistics & Probability”
11	crossing AND (curve OR curves)	
12	“delayed effect” OR “delayed effects”	
13	delayed AND treatment	
14	“treatment switch*” OR “heterogeneous patient*” OR “heterogeneous population*” OR “disease progression*”	
15	#10 OR #11 OR #12 OR #13 OR #14	
16	“hazard assumption” OR “hazard assumptions” OR time-to-event OR “time to event”	
17	#15 AND #16	
18	List of journals from the Web of Science category “Statistics & Probability”	
19	#17 AND #18	
20	#9 OR #19	
