# Supplementary material for: Methods for non-proportional hazards in clinical trials: A systematic review
Source: Stat Methods Med Res. 2024 Apr 9;33(6):1069–92. doi: 10.1177/09622802241242325 (PMC11162097; doi:10.1177/09622802241242325)
Supplement: sj-pdf-1-smm-10.1177_09622802241242325 - Supplemental material for Methods for non-proportional hazards in clinical trials: A systematic review [file sj-pdf-1-smm-10.1177_09622802241242325.pdf]

S Online Supplement

S.1 Notation

Table S1. Overview of notation used throughout the report

T	Time to event
C	Censoring time
x	Vector of covariates
Z	Treatment indicator, 1 if experimental, 0 for control
β	Vector of regression coefficients of x
γ	Regression coefficient of Z
N	Total sample size
δ	Event indicator, 1 if event, 0 if censored
$\lambda_0(t)$	Baseline hazard rate at time t
$\lambda^{(Z)}(t)$	Hazard rate of treatment group Z at time t (given x)
$\Lambda^{(Z)}(t)$	Cumulative hazard rate of treatment group Z $\int_0^t \lambda^{(Z)}(u)du$
$S^{(Z)}(t)$	Survival function of treatment group Z at time t (given x)
$Y_i(t)$	At risk indicator, $Y_i(t) = 1$ if $t \leq \min(T_i, C_i)$, 0 else
$Y(t) = \sum_i Y_i(t)$	Number of people at risk at time t
$Y^{(Z)}(t)$	Number of people at risk in treatment group Z at time t
$HR(t)$	Hazard ratio at time t (given x)
$aHR(t)$	Average HR up to time t
$cHR(t)$	Cumulative HR up to time t
$RMST(t)$	Restricted mean survival time up to t
t^*	Specific time point
$\tilde{t}$	Maximum follow-up time
$t_{(i)}$	i^{th} ordered distinct event time, $t_{(0)}$ defined as 0

S.2 Estimation approaches for NPH treatment effects

S.2.1 Kaplan-Meier based estimation approaches

The underlying basis of all approaches in this sub-section is the non-parametric Kaplan-Meier (KM) estimator of the survival function or the Nelson-Aalen (NA) estimator of the cumulative hazard function. The corresponding papers that fall into this category can be found in column A of Table S3.

The non-parametric KM estimate of the survival curve is defined as $\hat{S}^{(Z)}(t) = \prod_{t_{(i)} \leq t} \left(1 - \frac{d_{(i)}^{(Z)}}{Y^{(Z)}\left(t_{(i)}\right)}\right)$, where $d_{(i)}^{(Z)}$ is the number of events of treatment group Z observed at $t_{(i)}$ and the product iterates over the ordered distinct event times $t_{(i)}$. Alternatively, the NA estimate of the cumulative hazard function, i.e. $\widehat{\Lambda}^{(Z)}(t) = \sum_{t_{(i)} \leq t} d_{(i)}^{(Z)} / Y^{(Z)}\left(t_{(i)}\right)$, can be utilized. The treatment stratified KM estimates do not impose any modelling assumptions on $HR(t)$. Thus, any trajectory of $HR(t)$ can be incorporated.

The KM (NA) estimators result in step functions and thus the estimation of $HR(t)$ requires additional smoothing techniques, however. The $aHR(t^*)$ can be re-formulated as being an expression of the survival functions where the estimates might be obtained via KM [1].

A doubly-weighted Nelson-Aalen estimator that accounts for dependent censoring and treament-specific covariate distributions was introduced by [2] via inverse probability of treatment weighting and inverse probability of censoring weighting. The authors suggest to process the treatment-specific cumulative hazard estimators to cumulative treatment effect measures such as the cHR, relative risk, and differences in the RMST.

Quantiles and t-year survival rates can be obtained by standard statistical software which computes KM survival curves such as the R package `survival`. The R software package `survRM2` performs two-sample comparisons with the $RMST$ and also includes a function to perform an ANCOVA-type covariate adjustment. The SAS procedure LIFETEST can be utilized to obtain non-parametric estimates of the survival function and to carry out tests of homogeneity across strata or the association of numeric covariates on survival. The R package `AHR` provides software solution for the aHR.

S.2.1.1 Pseudo values

Pseudo-values are usually calculated from KM-based estimates, for example the $\widehat{RMST}(t^*)$ or $\hat{S}(t^*)$. The basic idea of the pseudo-value approach is to compute a quantity of interest at a given time t^*, e.g. $\widehat{RMST}(t^*)$ or $\hat{S}(t^*)$, and re-compute that measure with the ith observation dropped. Then, for example, $N\hat{S}(t^*) - (N-1)\ \hat{S}_{(-i)}(t^*)$, where $\hat{S}_{(-i)}$ denotes the KM estimate of the survival function with the ith observation dropped, is the so-called pseudo-value, and the ith entry of a new dependent variable. This value is an estimate of the survival function for the ith individual in this example. Hence, the interrelationship between the survival function and the cumulative hazard rate, $S^{(Z)}(t) = \exp(-\Lambda^{(Z)}(t))$, can be used to transform the pseudo value into an estimate of the log cumulative hazard rate $\Lambda^{(Z)}(t)$ by applying log-log transformation on the pseudo values. The pseudo value procedure reduces time-to-event data, that are usually defined by two variables (time, event indicator) to a single continuous variable, and allows modelling by common regression models, i.e. linear models with the treatment indicator being a covariate, with possibly transformed pseudo-values. [3]

Application of the pseudo value procedure on $\hat{S}(t^*)$, where the pseudo value $N\hat{S}(t^*) - (N-1)\hat{S}_{(i)}(t^*)$ is analysed by the linear regression model $x_i^T\beta + Z_i\gamma$, $i = 1,\dots, N$, gives estimates of $S^{(1)}(t^*) - S^{(0)}(t^*)$ through $\widehat{\gamma}$, assuming that x is held constant.

A weighted pseudo value approach to estimate survival probabilities at time t^* was introduced by [4]. They used the log-log transformation of the pseudo values and gave estimates for $cHR(t^*)$. The weights were proposed to overcome missing information on group membership in stem cell transplantation patients, where early death stops the search for a suitable stem cell donor.

Yang et al [5] utilized the pseudo-value approach for the $RMST$: Subject to investigation was the $RMST$ given survival up to a landmark t^* with (chosen) follow up time $t^* + w$, $cRMST(t^*, w)$, i.e. the expected life time within $(t^*, t^* + w)$ given survival up to t^*. Estimates of the $RMST$ were obtained via KM. Then, in a leave one out step, the $cRMST$ without the ith observation $c\widehat{RMS}T_{(-i)}(t^*, w)$ was computed. The individual differences $N^* c\widehat{RMS}T(t^*, w) - (N^* - 1)c\widehat{RMS}T_{(-i)}(t^*, w)$, with N^* being the sample size conditional on survival up to t^*, were then used as the dependent variable in a linear regression model $x_i^T\beta + Z_i\gamma$. If the multiple landmark time points are considered in a single analysis, covariate-time interactions might be utilized to obtain time-dependent covariate effects, e.g. $Z_i\gamma(t^*)$, with $\gamma(t^*) = \gamma_1 + t^*\gamma_2 + t^{*2}\gamma_3$, for the treatment component. The (time-dependent) treatment efficacy for such an approach can be evaluated via $\widehat{\gamma}(t^*)$ which estimates $cRMST^{(1)}(t^*, w) - cRMST^{(0)}(t^*, w)$.

Pseudo values were also considered by [6] and [7]. We did not encounter further effect measures in our literature review.

The R packages `pseudo` and `prodlim` as well as SAS and R functions in [8] are available for computing pseudo-values. Pseudo-values for $RMST(t)$ [but not for $S(t)$] can be calculated by PROC RMST in SAS.

S.2.1.2 Quantile regression

Quantile regression is an approach to systematically compare the estimated quantiles $t_\tau^{(Z)} = S^{(Z)^{-1}}(1-\tau)$, $Z = 1, 2$, and $\tau \in (0, 1)$.

A quantile regression approach has been established based on the generalized KM estimator [9] or alternatively, on martingale-based estimating equations [10]. In the latter case, the logarithm of the τth survival quantile $\ln\{t_\tau\}$ given covariate information is assumed to have a linear relationship with the covariates, i.e. $\ln\{t_\tau|x, Z\} = x^T\beta(\tau) + Z\gamma(\tau)$. The estimated treatment effect $\exp(\widehat{\gamma}(\tau))$ gives an expression of $\hat{t}_\tau^{(1)}/\hat{t}_\tau^{(0)}$ in the case of equality in x. A value larger than 1 of the above-mentioned ratio indicates a beneficial treatment effect at the respective quantile.

See [11] for an application to evaluate the long-term benefit of immunotherapy. Xue et al [12] developed a leave-one-out cross-validation approach for quantile regression.

An implementation of quantile regression including methods applicable to data with censored observations is available in the R package `quantreg`. The SAS procedure quantlife provides an implementation of quantile regression based on generalizations of the KM and NA estimator.

S.2.2 Stratified Cox model

The stratified Cox model is a well-known model that relaxes the PH assumption for the stratification variable(s). We excluded the basic stratified Cox model, where stratification by variables other than the treatment indicator is sufficient, from our literature review. The semi-parametric stratified Cox model, in particular, does not impose modelling restrictions on the trajectory of $HR(t)$ along the stratified variables. The hazard rate is defined as $\lambda^{(Z)}(t) = \exp(x^T\beta)\,\lambda_0^{(Z)}(t)$. The coefficients β can be estimated via the partial likelihood while the stratified baseline hazard $\widehat{\lambda_0^{(Z)}}$ can subsequently be obtained via the non-parametric Breslow estimate for each stratum.

Table S3 column B shows papers that belong to the category 'stratified Cox model'.

Again, estimation of $HR(t)$ requires additional smoothing techniques due to the step function nature of the Breslow estimate. An estimate of $cHR(t^*)$ was suggested by [13] and [14].

The R package `survival` and the SAS procedure phreg include the stratified Cox model.

S.2.3 Time varying coefficients for the hazard rates

Time-varying coefficients for the hazard rates $\gamma(t)$ can be utilized to incorporate, for example, a late or diluting treatment effect causing NPH. The hazard rate could be of the form $\lambda^{(Z)}(t) = \exp(Z\gamma(t) + x^T\beta)\,\lambda_0(t)$. Note that the time-varying treatment effect $\gamma(t)$ of those models can essentially be viewed as an interaction term of (some function of) time and the treatment indicator. The specific models differ in how those interactions are constructed.

The approaches to time-varying coefficients, or time-covariate interaction respectively, are plentiful and encompass change point models, fractional polynomials, spline-based approaches, other functional time-covariate interactions and the additive model. The admissible trajectory of $HR(t) = \exp(\gamma(t))$ depends on the complexity of $\gamma(t)$. The aHR, or weighted Cox regression, which will be discussed in Section S.2.3.5 further below can be seen as less complex summary measure in a time-varying coefficient setting. Columns of Table S3 that belong to the broader category 'time-varying coefficients' of Table S2 start with a 'C'.

Common approaches to fit the models are the maximization of the partial likelihood (e.g. [15], [16], or [17]), the full likelihood possibly via GLM routines (e.g. [18], [19], [20], or [21]) or via Bayesian approaches (see Table S3 column K). We added column K to Table S3 which indicates whether the corresponding paper took a Bayesian or a frequentist approach, such that the interested reader

might have a look at the corresponding papers to learn more about specific fitting procedures in detail.

S.2.3.1 Change point of the treatment coefficient

A late treatment effect, for example, might be considered by $\gamma(t) = \gamma\ I(t \geq t^*)$ where I denotes the indicator function, a diluting treatment effect through $\gamma(t) = \gamma_1 + \gamma_2\ I(t \geq t^*)$, with some (chosen) threshold value $t^* > 0$, $\gamma_2 > 0$. Johannes [22] suggested a grid search to find the location of the change point t^* associated to the highest partial likelihood value.
A generalized version of the single change point model, introduces a piecewise constant regression coefficient γ_k for each of the chosen disjoint time-intervals, i.e. $(t) = \sum_{k=1}^{K} \gamma_k I_k(t)$, where $I_k(t)$ is the indicator function which is unity if $t \in k^{th}$ interval and 0 else. A tree-based method to find multiple change points was suggested by [23]; a change point is chosen such that it maximizes the score test statistic. This process is iterated over the new time partitions resulting from the former step. In order to avoid over-fitting [23] also considered stopping criteria and a pruning mechanism.
Papers that were allocated to the category 'change point for time-varying effect' can be found in Table S3 column C.1.

S.2.3.2 Time varying coefficients with continuous paths

A smooth path of $\gamma(t)$ can be obtained by choosing a continuous function (in t). For example, $\gamma(t) = \gamma_0 + t\ \gamma_1 + t^2\gamma_2$ [24]. Gustafson [25] considered an additive hazard model where $\gamma(t) = \left(1 - \frac{t}{t^*}\right)\gamma_0 + \frac{t}{t^*}\gamma_1$ for $t < t^*$ and $\gamma(t) = \gamma_1$ else. Sauerbrei et al [17] chose $\gamma(t) = \gamma_0 + \log(t)\,\gamma_1$ as default and provided an algorithm for model selection: Firstly, start with an initial model, possibly incorporating functional forms and interactions of the covariates. Secondly, re-run the model on a restricted time interval $(0,\ t^*)$ and add relevant covariates from this step to the model of the entire time interval if necessary. Thirdly, for each covariate include time-varying coefficients, on the entire time interval, and add them to the final model if they provide a significant improvement. More flexible functions of $\gamma(t)$, obtained through fractional polynomials in time (see Section S.2.3.3), might be chosen if the enhanced model provides a significantly better fit as indicated by a deviance test.
Papers with some functional covariate-time interaction that do not belong to one of the more specific time-varying coefficient categories above or below can be found in column C.2 of Table S3.

S.2.3.3 Fractional polynomials

Fractional polynomials (FP) can be used to model a time-dependent treatment effect $\gamma(t)$. A $FP1(p)$ transformation of time is defined as t^p with some chosen p. By convention $FP1(0)$ is $\log(t)$. The time varying treatment coefficient with $FP1(p)$ transformation in t is then $\gamma(t) = \gamma_0 + t^p\gamma_1$. This can be extended to $FP2(p_1,\ p_2)$, this results in $\gamma(t) = \gamma_0 + t^{p_1}\gamma_1 + t^{p_2}\gamma_2$. Again, by convention, $FP2(p,p)$ refers to $\gamma(t) = \gamma_0 + t^p\gamma_1 + t^p\log(t)\ \gamma_2$. For applications see, for example, [26], [27], [18], or [17].

See Table S3, column C.3 to find papers which utilize FP to model time-varying coefficients.

S.2.3.4 Splines

A non-parametric approach for time-varying coefficients can be incorporated through the use of splines. A popular choice are restricted cubic splines. Splines, in general, require the selection of knots that slice the time scale into disjoint intervals. In the case of restricted cubic splines, local cubic polynomials are fitted [28]. The term "restricted" results from handling the tails linearly, i.e. from 0 to the first knot and from the last knot to infinity, the function is linear. We denote the corresponding time-varying treatment effect $\gamma(t) = \gamma_0 + \gamma_1 t + \sum_d B_d(t)\gamma_{d+1}$, where B_d denotes the corresponding basis function and the sum iterates over all basis functions. The number of basis functions depends on the number of knots. Applications can be found in [29] for an excess hazard model, [18] in a network meta-analysis and [27].

The spline approach can be extended to arbitrary choices of basis function. Let $\gamma(t) = \sum_d B_d(t)\gamma_d$. The B_d might, for example, be B-spline basis functions of arbitrary high degree and number of knots. The degree and the number of knots determine the number of basis functions or parameters, respectively, for the time-varying treatment coefficient. Overfitting can be avoided by introducing penalties on the flexibility. See, for example, [20] who exploited Poisson Likelihood regression, i.e. piecewise exponential hazards (see below), and penalized B-splines for survival model building. Similarly, [16] considered penalized B-splines and truncated splines. Argyropoulos and Unruh [19] also exploited the Poisson regression approach and include time-varying coefficients and multiple time scales via penalized cubic and thin plate splines. [30] considered time-varying coefficients via B-splines in an excess hazard model.

A spline-based time-varying coefficient in a Cox-model setting is available via the R package `dynsurv`. The R package `polspline` contains the function hare which utilizes linear splines to model the baseline hazard and covariate effects.

Column C.4 of Table S3 shows papers that utilized spline approaches in order to model time-varying coefficients.

Note, that for none of the aforementioned models, the time-varying coefficient approach is restricted to the treatment effect (or binary variables) but applies to covariates more generally.

S.2.3.5 Average hazard ratio and summary effect measures obtained by the weighted (partial) likelihood

The aHR can be calculated as summary measure over the follow-up period $\tilde{t}$ or some $t^* \leq \tilde{t}$. The summary measure has already been mentioned in Section S.2.1 based on KM estimates of the survival function. Plentiful estimation approaches of $aHR(t^*)$ based on the weighted partial likelihood are proposed in the literature. Consider the (true) model $\lambda^{(Z)}(t) = \exp(Z\gamma(t))\,\lambda_0(t)$. The idea is to estimate $\lambda^{(Z)}(t) = \exp(Z\gamma)\,\lambda_0(t)$ instead and interpret γ or $\exp(\gamma)$ as an average treatment effect by introducing weights on the partial likelihood

(see, for example, [31], [32], or [33]). Results from standard Cox regression depend on the censoring distribution and a weighted Cox regression with inverse probability of censoring weights are proposed. Schemper [33] has accordingly recommended choosing the weighting function $G(t) = S(t)/C(t)$, where $S(t)$ defines the event time survival function of the complete sample and $C(t)$ denotes the corresponding censoring survival function. For a review of differing definitions of the aHR, and different weighting functions , as well as an investigation of their performance see [33].

Such approaches are not restricted to an estimate of $aHR(t^*)$, however. Lin and León [34] obtained adjustment factors based on weights from the log-rank test where $\exp(\gamma)$ is the maximum treatment effect over the course of time.

An aHR estimator for the Yang and Prentice model (discussed in Section S.2.6) was proposed by [35].

Papers that approached the aHR or similar summary effect measures in a semi-parametric fashion through the weighted partial or pseudo-likelihood can be found in column C.5 (Table S3). Note, that we classified non-KM based estimations of the aHR, or weighted Cox regression, as time-varying coefficients in Table S2. Further notice, that papers which based the estimation of $aHR(t^*)$ on KM estimates are not subsumed in column C.5 but column A of Table S3.

The R package `coxphw`, and the SAS macro WCM provide weighted estimation of Cox regression which might be utilized to estimate the $aHR(t^*)$ with appropriate weights.

S.2.3.6 *Additive models*

Aalen's additive model also considers time-varying coefficients and NPH [36]. The additive model exploits the martingale representation $M(t)$ of the counting process $N(t)$, i.e. $M_i(t) = N_i(t) - \int_0^t Y_i(s)\lambda^{(Z_i)}(s)ds$. Note that $E(dM_i(t)|"history") = 0$ or equivalently $E(dN_i(t)|"history") = Y_i(t)\lambda^{(Z_i)}(t)dt$, where $dt \to 0$ and $dN_i(t) = 1$ denotes that the event occurred in an infinitesimal small interval following t, and 0 refers to no event in the aforementioned interval. The hazard rate is assumed to be additive in the predictors, i.e. $\lambda^{(Z)}(t) = x^T\beta(t) + Z\gamma(t)$. This leads to a twist in the interplay of covariates on absolute and relative differences of the hazard rates as compared to the multiplicative hazard model: keeping everything else constant, the treatment effect in an absolute sense is $\lambda^{(1)}(t) - \lambda^{(0)}(t) = \gamma(t)$ which is independent of the level of the other covariates, whereas the relative treatment effect is $HR(t) = \lambda^{(1)}(t)/\lambda^{(0)}(t) = 1 + \gamma(t)/x^T\beta$ which does depend on the level of the remaining covariates. If, for example, $\lambda^{(Z)}(t) = \lambda_0(t)\exp(x^T\beta(t) + Z\gamma(t))$ instead, this is the other way around.

Instead of optimizing the (partial) likelihood, least squares methodology is utilized. The individual squared error contribution at t is equal to $(dN_i(t) - Y_i(t)\lambda^{(Z_i)}(t)dt)^2$, where $\lambda^{(Z)}(t)dt$ is subject to estimation. Note that the estimates of $\lambda^{(Z)}(t)dt$ are not restricted to the interval $[0,1]$ and thus, might not be interpretable as probabilities.

The increments $\lambda^{(Z)}(t)dt$ will typically be estimated poorly. Estimates of $\Lambda^{(Z)}(t)$ can be obtained through $\sum_{i\geq 1:t_{(i)}\leq t}\widehat{\lambda^{(Z)}}\left(t_{(i)}\right)dt$, where the summation over the increments achieves stability in the estimates. (Chapter 4.2.1 in [36])
Applications of the additive model can be found in [37] and [38]. Dunson and Herring [39] placed a model selection prior on an additive-multiplicative survival model and restrict the additive part to ensure non-negative hazards. Martinussen and Pipper [40] developed an odds-of-concordance $\frac{P(T^{(0)}>T^{(1)})}{P(T^{(1)}>T^{(0)})}$ effect measure based on Aalen's additive model, where $T^{(Z)}$ refers to a survival time of group Z.
The R package `timereg` contains the additive model.
Papers focusing on the additive model can be found in column C.6 of table S3.

S.2.4 Transformation models with time-covariate interaction

In this section we placed the Royston-Parmar model as well as the conditional transformation model (CTM). Both approaches have in common that time and covariate dependent model quantities are modelled via splines and spline by covariate interaction. Also both approaches can be motivated as being generalizations of chosen parametric models. The Royston-Parmar and the CTM are very similar for appropriately chosen reference functions.

The starting point of the Royston-Parmar model is a transformation of the survival function which is denoted by $g(S^{(Z)}(t))$. Initially, the function is assumed to be linear in the covariates and the treatment indicator, i.e. $g(S^{(Z)}(t)) = g(S_0(t)) + x^T\beta + Z\gamma$. The point of reference is either the Weibull distribution and PH or the log-logisitc distribution and proportional odds. We focus on the Weibull case. Then, $g(S^{(Z)}(t)) = \ln\{-\ln\{S^{(Z)}(t)\}\} = \ln\{\Lambda_0(t)\} + x^T\beta + Z\gamma$. Note, that the Weibull AFT arises if $\ln\{\Lambda_0(t)\}$ is linear in $\ln\{t\}$. To allow for more flexibility, $\ln\{\Lambda_0(t)\}$ is replaced by a restricted (natural) cubic spline function of $\ln\{t\}$, $s(t;\omega) = \sum_d B_d(\ln\{t\})\omega_d$, where ω is a parameter vector, and the accelerated failure time interpretation is lost. In the log-logistic case, the same path is followed in analogy, except that the log-cumulative hazard function is replaced by the log-cumulative odds function, that is the log-odds of an event occurring in the interval $(0,t)$.
NPH (or non-proportional odds) can be incorporated by an interaction of the natural cubic splines and the treatment indicator, i.e. $s(t;\omega) + s(t,Z;\gamma)$, with $s(t,Z;\gamma) = \sum_d ZB_d(\ln\{t\})\gamma_d$, where γ is a vector of parameters. Further covariates might be included in $s(t,Z,X;\omega)$ in the same fashion to allow for more non-proportional effects. For sake of comparison with the conditional transformation model (CTM) we call the functions $s(\cdot)$ transformation functions.

For more detailed information about the Royston-Parmar model see [21]. An implementation of the Royston-Parmar model is available through the R package `flexsurv` [41].
The CTM formulates $S^{(Z)}(t)$ as $1 - G(\sum_d h_d(t,x,Z))$, where G is a chosen cumulative distribution function (cdf). If G is the minimum extreme value

distribution, the Cox model is a special case for appropriately chosen (or estimated) transformation functions h_d. In general, the transformation functions h_d consist of interactions of time and covariates as well as parameters which are subject to estimation. The transformation function for the treatment indicator could for example be $h(t,Z) = \sum_d B_d(t)Z\gamma_d$. Möst et al [42] suggested interactions of penalized B-splines in time t and B-splines or linear basis functions of covariates.

Consider the case where the reference cdf of the CTM is that of the standard minimum extreme value distribution $G(\cdot) = 1 - \exp\{-\exp\{\cdot\}\}$ and the Royston-Parmar has the Weibull model as reference as described above. Moreover, let all covariates be included in the NPH manner as illustrated above for the Royston-Parmar and the CTM. Then, the main difference between the Royston-Parmar and the CTM as reported here is the choice of basis functions, natural cubic splines vs penalized B-splines as proposed by [42], and the time scale on which the basis functions are computed, $\ln\{t\}$ vs t.

Papers that focus on the Royston-Parmar model or the CTM can be found in column F of Table S3. Note that all papers in that column focus on the Royston-Parmar models except for the paper [42], which is about the CTM.

S.2.5 Joint models

A NPH model can also be obtained by jointly modeling a longitudinal and a survival outcome. Certain time and treatment-dependent components of the longitudinal model might, for example, be the covariate input of the survival model. Let, $\eta(t,Z) = \gamma_L g(t,Z)$ be the conditional expectation of the longitudinal outcome at t (in the absence of further covariates), with $g(t,Z)$ being a chosen function of treatment and time and γ_L the corresponding coefficient vector in the longitudinal model. Then, the hazard might be modelled as $\lambda^{(Z)}(t) = \exp\left(x^T\beta + Z\gamma + \eta(t,Z)\gamma_2\right)\lambda_0(t)$. In this scenario, the treatment has a direct constant impact on the $HR(t)$ through γ as well as an indirect time-varying impact through $\eta(t,Z)\gamma_2$. See [43] for a model of that kind, that also includes further covariates and random effects in the longitudinal component.

Articles focusing on joint models can be found in column D of tables S3.

Xu et al [43] also discuss the posterior distribution of the $aHR(t^*)$ in a Bayesian setting.

The package and macro `JM` provides a software solution for joint modeling of longitudinal and time-to-event data in R and SAS, respectively. The SAS macro is based upon NLMIXED, MIXED, GLIMMIX, and LIFEREG.

S.2.6 Short- and long-term HR

The methods of Section S.2.3 accounting for a time-varying treatment effect can essentially be considered as time-covariate interaction. Alternatively, parametric functions of the $HR(t)$ can be assumed, where the time-varying effect results from interactions of parameters with time-dependent baseline measures such as $\Lambda_0(t)$, or $S_0(t)$. The models in this sub-section differ from fully parametric

approaches in that they have non- or semi-parametric components of the baseline measures. We gathered those models in columns F.1 and F.2 in Table S3.

Such a model was introduced by [44] and further studied by [45]. A potentially non-proportional treatment effect can be incorporated by the hazard function $\lambda^{(Z)}(t) = \frac{\exp(x^T\beta)}{\exp(-\gamma_1 Z)\ S^{(0)}(t)+\exp(-\gamma_2 Z)(1-S^{(0)}(t))}\lambda_0(t)$. The model is also termed short- and long-term HR model: Here, $\exp\{\gamma_1\}$ is the short-term and $\exp\{\gamma_2\}$ is the long-term hazard ratio of the treatment variable, respectively. This model includes strictly increasing and decreasing HR scenarios as well as PH if $\gamma_1 = \gamma_2$. Crossing survival curves as well as no initial treatment effect are also sub-models of the Yang and Prentice model [44]. The Yang and Prentice model readily delivers an estimate of $HR(t)$. An estimator of $aHR(t^*)$ has also been established [46].

A generalization of the PH model has also been considered through $\lambda^{(Z)}(t) = \lambda_0(t)\exp\left\{x^T(\beta_1+\beta_2)+Z\ (\gamma_1+\gamma_2)+\left(\exp(x^T\beta_2+Z\ \gamma_2)\ -1\right)\log[\Lambda_0(t)]\right\}$, where β_2 and γ_2 are additional model parameters. The PH model is obtained by $\beta_2 = \gamma_2 = 0$. The corresponding survival function equals $S^{(Z)}(t) = \exp\left\{-\exp(x^T\beta_1+Z\gamma_1)\Lambda_0(t)^{\exp(x^T\beta_2+Z\gamma_2)}\right\}$. Crossing survival curves are also possible. The baseline hazard function can, for example, be estimated via splines. [47]

Papers that have the Yang and Prentice model in focus as well as the model introduced by [47] can be found in Table S3 column E.

R Software packages for the Yang and Prentice model are available: YPPE which estimates the baseline quantities via piecewise exponential distribution [48], YPBP which estimates the baseline distribution via Bernstein polynomials [49] and YPmodel.

S.2.7 Frailty models

Frailty can introduce NPH on the population level even if the PH assumption holds on the individual level given the unobservable characteristics. Frailty, or unobserved heterogeneity, might be induced by unmeasured or unmeasurable covariates. High frail individuals are prone to "early" events due to a high individual or conditional hazard and vice versa. We denote the population hazard as the hazard rate with the individual frailty being "integrated out" in this paragraph. Assume an extreme case, with lower population hazard for the treatment group early on but higher population hazard at later stages, as compared to the control group. This is not necessarily a sign of a harming long-term treatment effect. Instead, this could be an indicator of successful treatment as this might be caused by long-term survival of high-frail individuals in the treatment group. The most straightforward frailty model can be expressed as $\lambda^{(Z)}(t|U) = U\exp\left(x^T\beta+Z\ \gamma\right)\lambda_0(t)$, with the frailty random variable $U \geq 0$. The corresponding population hazard rate is $\lambda^{(Z)}(t) = \int_0^\infty \lambda^{(Z)}(t|u)f_U(u|T\geq t)du$ with $f_U(u|T \geq t)$ being the frailty density of survivors. Analytical expressions for

$\lambda^{(Z)}(t)$ are available if $f_U(u)$ is, e.g., the gamma density or another density of the Power Variance Family (see, for example, Chapter 6.2.3 in [36]) among others. See Aalen et al [36] or Wienke [50] for a thorough discussion of individual frailty. A tutorial on frailty models that also discusses the population hazard ratio can be found in [51].
We took a very broad perspective of frailty in this work and also put, for example, cure-rate models in this category which could be motivated by a binary frailty model [50].
Unobserved heterogeneity has, for example, been considered through semi-parametric transformation models [52], spatially correlated frailty models [53], time-varying frailty models [54] and random delayed treatment effect two-point cure rate- [55], as well as responder-no-responder-models [56].
Papers having a frailty perspective are marked in column G of Table S3.
The SAS procedures phreg and nlmixed, the R packages `survival`, `coxme`, `frailtyEM`, and `frailtypack` provide frailty models.

S.2.8 Fully parametric models

For the sake of discussion we separate parametric approaches into four classes: piecewise exponential, accelerated failure time (AFT), first hitting time (FHT) and GLMs.

S.2.8.1 Piecewise exponential hazards

The stratified piecewise exponential model assumes a constant hazard rate $\lambda^{(Z)}(t) = \lambda^{(Z)}_{(k)}$ within the specified interval $t \in [t_{k-1}, t_k)$, with k= $1, \ldots, K$, $t_k < t_{k+1}$, $t_0 = 0$ and $t_K = \infty$. Then, the $HR(t)$ is piecewise constant. Further covariates might be included in the usual PH manner: $\lambda^{(Z)}(t) = \exp(x^T \beta^{(Z)})\, \lambda^{(Z)}_{(k)}$, where (elements of) $\beta^{(1)}$ might be equal to (their counterparts in) $\beta^{(0)}$ and t is in the kth time interval. The stratified piecewise exponential model approaches the stratified semi-parametric Cox model if the borders of the time intervals are set by the distinct event times.
Hagar et al [57] discussed a Bayesian non-proportional multiresolution hazard model. The hazard is piecewise constant and treatment specific. Across partitions, the hazard parameters might be correlated what can be regarded as a smoothening attempt. A second smoothening approach is imposed through the merging of adjacent time intervals if mortality patterns are statistically similar. Constant or time-varying covariate effects might also be added to the piecewise exponential model.
Papers that model time variant covariate effects via the piecewise exponential model can be found in Table S3, column H.1.
With respect to fitting procedures, the Poisson regression model (with an adequate offset) and its software implementations can be exploited (see, e.g., [58], [19] or [20]). The R packages `eha` and `pch` contain the piecewise exponential model. An R software implementation of the multi-resolution hazard model is available with the package `MRH`.

S.2.8.2 AFT and generalized additive models for location scale and shape

The AFT model assumes a distribution π with parameter vector $\theta^{(Z)}$ for the survival time T. The parameters in θ are (partially) dependent on group membership Z and maybe of further covariates. In particular, $\log(T) = x^T \beta + Z\, \gamma + \sigma \epsilon$, where the distribution assumption about the error term determines π. Frequent choices of π are the Weibull, Log-logistic, and Log-normal distribution. AFTs usually result in NPH, with the Weibull being a prominent exception. AFTs can be extended by also modelling scale and shape parameters, σ in the above example, via link functions and covariates. In the generalized case, even the Weibull model contains NPHs [59]. An extension to interval censored data and gamma frailty can be found in [60].

Umbrella distributions like the generalized gamma [61] and the generalized F distribution [62], which include “standard” distributions as special cases, have also been modelled in the AFT context and its generalizations. Extensions which consider all parameters as function of covariates were also discussed in [63].

An approach to dimension reduction was introduced by [64] with a focus on genomic data, utilizing the continuum power regression (CPR) framework. The CPR-step is supposed to obtain a dimension reduction in the covariates and includes OLS, partial least squares, and principal components regression as special cases. Censoring is accounted for by adding the mean residual lifetime on censored observations in the CPR-step. The K “remaining” components are then the covariate input of a generalized F or a semiparametric AFT model.

Delayed treatment effects [65] and cure rate models [66] were also considered for the Weibull model.

Papers with a focus on the AFT model or generalizations thereof are marked in column H.2 of Table S3.

A software implementation of the generalized gamma and generalized F distribution and other (user-defined) distributions is available via the R package `flexsurv` [41], where also more than one parameter might depend on covariates. The R package `brms` provides Bayesian parametric survival models. The R package `spBayesSurv` offers spatial as well as non-spatial Bayesian (generalized) AFTs (and others). The R package `mpr` can be used to fit, for example, Weibull models where both parameters depend on covariates. The R package `gamlss.cens` is an add-on package to GAMLSS for the purpose of fitting censored versions of an existing GAMLSS family distribution. The SAS procedure lifereg also offers an implementation of AFT models.

S.2.8.3 First hitting time

First hitting time (FHT) models approach the survival distribution via an underlying unobservable health process $H(t)$. An observable component of the process is the event or, more general a transition into another state. Typically, the event is defined to happen if $H(t) \leq 0$ for the first time. The Wiener process is commonly chosen for $H(t)$. This leads to T being inverse Gaussian distributed. The parameters of the Health process might be a function of covariates and

possibly of random effects [67]. Race and Pennell [68] added random effects utilizing the Dirichlet Process to model subject-specific initial state and drift of the Wiener process. Yu et al [69] modeled the drift parameter via cubic B-splines. He et al [70] considered a deterministic decay path where random shocks might cause the event “prematurely” in a hip fracture setting.
FHT models are marked in column H.3, Table S3.

S.2.8.4 GLMs & other parametric approaches

If the event indicator is regarded as the target variable, a Poisson GLM with log-link and an adequate offset is equivalent to the piecewise exponential hazard likelihood. Hence, Poisson GLMs have been utilized to fit the piecewise exponential survival model or as an approximation to a general survival likelihood by letting the time intervals for the distinct hazard parameters become small [19]. We mentioned this already in the discussion about time-varying coefficients. We did not classify those papers into the GLM category as they essentially modeled the common survival likelihood via GLM routines and hence, we feel, those papers are more adequately categorized into other classes, time-varying coefficients for example.
Others, however, took a more genuine GLM approach. Among them [71] who exploited the longitudinal nature of multiple sclerosis data-set to model transitions in the disability progression. Motivated by oncology studies with NPH, where events are observed through periodic screenings, [72] considered discrete time. Consequently, GLMs at the distinct time points including only at-risk individuals at the corresponding follow-up time $t_{(j)}$, i.e. $Y_i\left(t_{(j)}\right) = 1$, are suggested.
GLMs might be incorporated into other methods, trees, for example (see Section S.2.9).
Other parametric approaches including GLMs can be found in column H.4, Table S3.
GLM routines from standard statistical software can be used, for example, the SAS procedure glm.

S.2.9 Machine learning approaches

Wey et al [73] suggested an average survival model which is a weighted sum of parametric, semi-parametric, and non-parametric models. The weights in the suggested model are obtained through the minimization of a loss function. Distinct treatment group survival curves are obtained by “averaging out” the remaining covariates or confounders respectively. Inference regarding the treatment effect is then drawn from the difference in RMST of the treatment and the control group.
Lowsky et al [74] discussed a K-nearest neighbor approach for estimating survival curves via weighted Kaplan-Meier. The K-nearest neighbors of a new observation are determined by its distance to the covariate vectors in the data set. Then, a weighted KM curve is estimated. The weights for each observation are reciprocal to the distance to the new covariate vector and affect the number

of deaths as well as the size of the risk set at each event time. This procedure could, for example, be stratified by the treatment indicator in order to quantify the treatment effect given the remaining covariates.
The former two approaches can be found in column I.4, Table S3.
Alternative approaches include trees and survival forests. Papers concerning trees and forests are marked in column I.1, Table S3. The tree-based method by [23] to find change points for time-varying coefficients has already been discussed above.
A Bayesian additive regression tree (BART) for survival data with NPH was considered by [75]. The Likelihood is specified via probit regression at the distinct event times. The probability of an event, conditional on no previous events, at a given event time is derived via the BART. The BART is an ensemble of trees where the splitting criteria at the internal nodes within each tree are set by the event time and covariates. For a given covariate-time input the function value at the terminal node is then summed up over each tree. This estimate is used as the input of the standard normal cdf to obtain the probability of an event.
Soft BART (SBART) was considered for interval-censored data by [76]. The SBART extends the BART with a smooth regression function and better ability to remove irrelevant predictors.
Survival forests as well as an improved splitting criterion were discussed in [77]. A survival forest for a joint model was developed by [78].
Neural networks for censored survival data were discussed by [79] and [80] . Neural networks are marked in column I.2, Table S3. Kernel smoothing based approaches can be found in [81] regarding the hazard ratio and [82] with respect to differences in survival rates. Kernel smoothing based approaches are marked in column I.3, Table S3.
The R package `trtf` contains transformation trees and forests that can be utilized for time-to-event analysis. The SBART for discrete time-to-event analysis is implemented in the R package `BART`. The R package `mboost` includes a gradient boosting algorithm for right-censored data.

S.2.10 Other approaches

Some papers were difficult to categorize into one of the previous sections.
Among the papers in this section is [83], which discussed inverse probability of censoring weights (IPCW) approaches, the structural nested model (SNM), and the rank preserving structural failure time model (RPSFTM) in the context of treatment switching. Similarly to the SNM, the RPSFTM assumes that the treatment decreases or increases the survival time by the factor $\gamma > 0$. More precisely, $T^{(1)} = \gamma T^{(0)}$ and $\gamma > 1$ indicates a beneficial treatment effect. With that factor, counterfactual survival times are computed, i.e. $u_i = (\text{time } i \text{ spent in control group}) + \frac{\text{time } i \text{ spent in treatment group}}{\gamma}$. The parameter γ itself is found via a grid search, where the optimization criterion is a test statistic that compares the estimated survival curves of the placebo and the treatment group,

where for the latter the counterfactual survival times are utilized. The value for γ that makes the two groups most alike in the sense of the test statistic is the point estimate. The R package `rpsftm` provides a software solution for the RPSFTM. The IPCW approach, where the weights might be utilized to compute KM curves or weighted partial likelihood estimates, attempts to account for informative censoring.

Further proposals are a semi-parametric proportional likelihood ratio model [84], and concordance regression, where, brought into the two-sample setting, the likelihood is based upon $P(T^{(1)} > T^{(0)})$ [85]. A Bayesian non-parametric dependent Dirichlet process for modeling the time-to-event distribution was studied by [86]. Chen and Wang [87] apply an accelerated hazard model, where the hazard is equal to $\lambda^{(Z)}(t) = \lambda_0(t \exp\{Z\gamma + x^T\beta\})$, and the baseline hazard is a smoothed non-parametric estimate. The authors suggest that the accelerated hazard model might be a good choice if hazard rates are similar after the start of follow-up but go apart due to different ageing processes.

In column J, Table S3, we marked approaches that did not properly fit into other categories.

S.3 Hypothesis tests for equality of survival curves

S.3.1 Log-rank tests

The standard log-rank test statistic [88, 89] is the most widely used statistical test to compare the overall survival of two groups. The log-rank test is defined as

$$M_w = \frac{\sum_{i=1}^{D} w(t_{(i)})\Big[d_{i1} - Y^{(1)}(t_{(i)})\frac{d_i}{Y(t_{(i)})}\Big]}{\Big[\sum_{i=1}^{D} w(t_{(i)})^2 \frac{Y^{(1)}(t_{(i)})}{Y(t_{(i)})}(1 - \frac{Y^{(1)}(t_{(i)})}{Y(t_{(i)})})\frac{Y(t_{(i)})-d_i}{Y(t_{(i)})-1} d_i\Big]^{1/2}},$$

with the weight function $w(t_{(i)}) = 1$. The number of distinct event times of the pooled sample is denoted by D, d_i is the number of events at $t_{(i)}$, and d_{i1} refers to the number of events at $t_{(i)}$ in the experimental treatment group [90].

As the shape of the survival curve influences the power of the test, multiple proposals for the weight function $w(t)$ sensitive to particular NPH patterns are available [91].

Royston and Parmar (2020) [91] give an overview of the weight functions used for different NPH patterns, e.g. the Fleming-Harrington weight function $w(t_{(i)}) = \hat{S}\left(t_{(i)}^{-}\right)^{1}\left(1 - \hat{S}\left(t_{(i)}^{-}\right)\right)^{0}$, denoted $G^{1,0}$, or $w(t_{(i)}) = \hat{S}\left(t_{(i)}^{-}\right)^{0}\left(1 - \hat{S}\left(t_{(i)}^{-}\right)\right)^{1}$, denoted $G^{0,1}$, for early or late effects, respectively. Note that t^{-} denotes the time just before time t. For testing that survival in the treatment group is stochastically less than or equal to survival in the control arm ($H_0 : S_0(t) \leq S_1(t)\ \forall\ t \geq 0$), Magirr and Burman (2019) [92] propose *modestly weighted log-rank* tests. It is a variation of the weighted log-rank test which under arbitrary weights does not control the risk to conclude that a new treatment is more efficacious than standard care when it is uniformly inferior in terms of the survival function. Treatment may be uniformly inferior in terms of the survival function, but still, there may be time points at which the treatment has a favorable hazard. The log-rank test works at the level of the hazard function, so if enough weight is put on the possibly small time interval with a favorable hazard, the treatment is declared significantly better than the control, even though this local benefit does not translate into any survival benefit. The modestly weighted test is constructed in such a way, though, that this fallacy can never happen. The weights are set to $w(t_{(i)}) = \frac{1}{\max\{\hat{S}\left(t_{(i)}^{-}\right), \hat{S}(t^*)\}}$ with $\hat{S}(t^*)$ denoting the KM estimate at a certain time t^* based on the pooled data from both treatment arms. The choice of t^* is a trade-off. A bigger value of t^* results in lower weights for early events, which is useful for delayed effects, on the other hand too large t^* will lead to unnecessarily high weights $w(t_{(i)})$ for late events.

Sample size formulas for different weighted log-rank tests are given by e.g. Yung and Liu (2020) [93] and their R package npsurvSS. Wei and Wu (2020) [94] and Wu and Wei (2022) [55], Ye and Yu (2018) [95] provide R-code for their sample size formulas derived from weighted log-rank tests for cancer immunotherapy trials with delayed treatment effects.

Various authors also investigated the use of weighted log-rank tests in group-sequential trial designs. Group-sequential trial designs plan interim analyses at pre-specified time points. The interim analysis can lead to early efficacy or futility stopping because of either sufficiently convincing results or a further investigation not being justifiable.

The use of weighted log-rank test statistics in group-sequential designs can lead to a misspecified covariance of the test statistic due to the incorrectly estimated information fraction. The information fraction is not proportional to the number of interim events for weighted log-rank tests in general, e.g. late events on which the weight function usually places more weight in delayed treatment effect settings might be unavailable during interim analyses. Interim analyses are argued to be only sensible if a fixed time horizon for the final (primary) analysis is specified and if sufficient information up to the time horizon is available for the interim analysis [96].

Brummel and Gillen (2014) [97] focus on monitoring the weighted log-rank test statistic in group-sequential designs where information growth is nonlinear and propose using a constrained boundaries approach to maintain the planned operating characteristics of a group-sequential design. Hasegawa (2016) [66] proposes a semiparametric information fraction for group-sequential designs with delayed treatment effects. Kundu and Sarkar (2021) [98] focus on the deviation of information fractions in weighted log-rank test from that of standard log-rank test and propose a decomposition of effects on information fractions to provide a reasonable and practically feasible range of information fractions to work with.

Li et al (2021) [99] propose a group-sequential design based on the piecewise log-rank test, Zhang and Pulkstenis (2016) [100] provide closed-form solutions for the power and sample size calculation for group-sequential designs and Magirr and Jiménez (2022) [101] give practical guidance for the use of *modestly weighted log-rank tests* in group-sequential trials.

S.3.2 Kaplan-Meier based tests

Kaplan-Meier-based tests are another class of tests for testing the null hypothesis of equal survival in the two treatment groups. These tests are based on KM estimates or restricted mean survival time (RMST).

Weighted Kaplan-Meier tests are based on the weighted sum of the differences of the KM estimates of the survival curves. Uno et al (2015) [102] proposed a weighted Kaplan-Meier test with weights proportional to the observed standardized difference of the estimated survival curves at each time point.

The unweighted Kaplan Meier test results in the difference between two RMSTs describing the mean event-free survival time up to a pre-defined time point t^* [103]. Since equality of survival curves implies equal RMST, we can also test for the difference in RMST between treatment groups $\Delta(t^*) = RMST^{(1)}(\text{t}^*) - RMST^{(0)}(\text{t}^*) = \int_0^{t^*} S^{(1)}(t)\,dt - \int_0^{t^*} S^{(0)}(t)\,dt$ being zero, $H_0 : \Delta(t^*) = 0$. The null hypothesis can be tested using the Wald statistic $M_{RMST}(t^*) =$

$\sqrt{n}\ \widehat{\Delta}(t^{*})/\widehat{\sigma}(t^{*})$, with $\widehat{\sigma}(t^{*})$ denoting the variance of $\sqrt{n}\,\{\,\widehat{\Delta}(t^{*}) - \Delta(t^{*})\,\}$. Tests based on RMST do not rely on the PH assumption but are also not specifically designed to detect crossing survival curves [103].

Test procedures based on RMST are proposed by e.g. Horiguchi et al (2018) [104], Lawrence et al (2019) [105] and Sun et al (2018) [106].

The pre-specified time point t^{*} for RMST is selected data dependently in Horiguchi et al (2018). Therefore, a set of potential times $t^{*} = \{t_1^{*}, \ldots, t_K^{*}\}$ with a fixed number K is assumed. The null hypothesis that there is no difference between 2 event time distributions against a two-sided alternative $\Delta\left(t_k^{*}\right) \neq 0$ is tested with the test statistic $M_{RMST2} = \max_{t \in t^{*}} |M_{RMST}(t^{*})|$. The distribution under the null hypothesis is obtained using a wild bootstrap procedure. The approach of Horiguchi et al (2018) [104] is available in the R package `survRM2adapt`. For cure rate survival models Sun et al (2018) [106] compare different tests for cure rate survival data and showed in a simulation study that Kaplan-Meier-based tests (RMST test and weighted Kaplan Meier test) perform best among the considered test, e.g. log-rank, Wilcoxon rank test.

Rauch et al [1] compared two test statistics for the average hazard ratio aHR testing the null hypothesis $H_0 : aHR \geq 1$ to the standard log-rank test using the hazard ratio in settings with different underlying event times and censoring distributions. The two test statistics used for testing the average hazard ratio differ in their independent increments property. The comparison showed the advantage of the average hazard ratio tests in terms of power in NPH settings.

Window mean survival time proposed by Paukner and Chappell [107] keeps the interpretability of RMST and unweighted log-rank tests and improves the power to detect differences in survival curves under NPH caused by late crossing or diverging curves. The difference in window mean survival time of the two treatment groups is the area between the two survival curves from $t = t_1^{*}$ to $t = t_2^{*}$, with $0 \leq t_1^{*} < t_2^{*} \leq \tilde{t}$. The null hypothesis is that the difference in window mean survival time is zero. The test statistic is calculated by the ratio of the estimated difference in window mean survival time and its estimated variance. The simulation study of Paukner and Chappell [107] showed that the test of window mean survival time has higher power compared to the weighted log-rank test if the PH assumption holds.

Sample size formulas based on the RMST test are provided by e.g. Tang [108], Royston and Parmar [109]. Yung and Liu [93] provide a R package `npsurvSS` for sample size and power calculations based on Kaplan-Meier-based tests.

Brückner and Brannath (2017) developed group-sequential designs for the hazard ratio and proved that the sequential tests based on the average hazard ratio are asymptotically multivariate normal with independent increments property.

An approach using the weighted Kaplan-Maier test for the calculation of stage-wise p-values in adaptive survival trials allowing to use discrete surrogate information for the interim analysis while controlling the type I error rate was

proposed by Brückner et al [110]. Sample size re-estimation using Kaplan-Meier-based tests is investigated by Wang [111].

S.3.3 Combination tests

Combination tests combine tests within a class or across classes of tests. The idea underlying the combination of tests is the difficulty to predict the existence and severity of NPH caused by e.g. delayed treatment effects. Combination tests allow covering various scenarios. The maximum combination (max combo) test is an example of such combination tests and is defined as the maximum of several weighted log-rank test statistics. Using the Fleming-Harrington weighted log-rank test denoted $Z_{G^{\rho,\ \gamma}}$ the max combo test is defined as

$$Z_{max} = max_{\rho,\gamma}\{Z_{G^{\rho_1,\ \gamma_1}}, Z_{G^{\rho_2,\ \gamma_2}}, \ldots, Z_{G^{\rho_K,\ \gamma_K}}\}$$

where $Z_{G^{\rho_k,\ \gamma_k}}$ denotes one of K different weighted log-rank tests. However, the max combo test is not restricted to the Fleming-Harrington weight function in the log-rank tests. The p-value of the maximum combination test can be calculated based on the multivariate normal distribution. Ghosh et al [112] developed group sequential designs using two (*modestly) weighted log-rank tests* for the max combo test statistic. Ristl et al [113] investigated different sources of non-proportionality such as e.g. delayed treatment effect, disease progression, predictive biomarker subgroups, treatment switch after progression, and their effect on the power of weighted log-rank tests and maximum combination tests. Ristl et al [113] provide the R package nph to perform the statistical tests and to simulate survival data.
Sample size procedure for maximum combination tests are e.g. available in Tang (2021).
The use of combination tests specifically the max combo test in group sequential trial designs was investigated e.g. in Li et al [114], Wang et al [115], Prior [116]. Li et al [114] investigated obtaining the group sequential boundaries and the empirical power by simulation procedures for delayed treatment effects, whereas the approach of Wang et al [115] with an R-package GSMC available on GitHub is simulation free. Prior [116] investigated the use of different weighting functions in the maximum combination across the time points allowing flexibility to the accrued data.
Combination tests are not only restricted to the weighted log-rank test but can also involve other classes of tests, e.g. Royston and Parmar [117] combine the Cox test with a test of the RMST difference by obtaining the p-value of the combination test via selecting the smallest p-value of the single tests. The Cox test is based on the difference in log partial likelihoods of the Cox PH model with the binary treatment indicator as only covariate. It is closely equivalent to the standard log-rank test [117]. León et al [118] combine weighted log-rank tests with the RMST test. Chi and Tsai [119] propose the combination of weighted log-rank tests with weighted Kaplan-Meier tests. Zhang et al [15] propose a Cauchy combination test of multiple single change-point (CauchyCP) regression models.

S.3.4 Other tests

Besides the Kaplan-Meier-based test, the log-rank tests, and the combination approaches, we identified also articles that proposed hypothesis testing methods not fitting in either of these classes. For instance, Gorfine et al [120] proposed a test for K groups based on sample–space partitions, which is implemented in the R package `KONPsurv`.

A modification of the Kolmogorov Smirnov test was proposed by Fleming et al [121] who compare their proposal to the standard log-rank test and the Wilcoxon rank test for censored observations [122] and showed higher power under NPH. Sooriyarachchi and Whitehead (1998) propose a binary method for testing whether the survival curves of two treatment groups are equal. This approach needs the discretization of the time. The time intervals underlying the discrete time should include equal numbers of events. The effect measuring the treatment difference is the log odds ratio of the probability surviving past time point t^* in the two treatment groups. The test statistic is derived from the log-likelihood of the log odds ratio and the nuisance parameter of the probabilities. Permutation procedures can be used to obtain the distribution of the test statistic under the null hypothesis of equal survival functions. For combinations approaches permutation approaches were suggested by e.g. Brendel et al (2014) [123], Royston and Parmar (2016) [117], Ditzhaus and Friedrich (2020) [124], Ditzhaus et al (2021) [125] and Ditzhaus and Pauly (2019) [126]. The approaches [123, 126, 124] suggest a combination of weighted log-rank statistics targeting a comprehensive range of alternatives. These tests can be applied with the R-package `mdir.logrank`. Ditzhaus et al (2021) [125] provide the R package `GFDsurv` for their proposed approach.

For the application of newly proposed methods providing the corresponding software is of advantage. In some settings, numerical aspects are important in the development of such software. For most existing test statistics, Riemann integration is used. However, under complex NPH pattern involving high dimensional numerical integration this approach might not be feasible. Therefore, Tang [108] proposes a sample size and power calculation method for log-rank tests and RMST tests via product integration and provides sample SAS code as online supplementary material.

S.4 Classification of methods proposed in selected articles

Table S2. Classification of methods proposed in selected articles. A description of the methods can be found in Section 5 and Section 6. The corresponding references for the IDs are given in S5

	Hypothesis Tests				Effect Estimation and Regression Models									
ID	Log-rank tests	Kaplan-Meier (KM) based tests	Combination tests	Other tests	KM based estimation	Stratified Cox	Time varying coefficients	Transformation models	Short- and long-term HR	Joint models	Frailty models	Parametric Models	Machine Learning	Other
1	x													
9	x	x	x	x										
14						x	x	x				x		
16					x									
19			x											
20							x							
21								x						
22	x	x												
26					x									
27							x							
28			x											
30	x						x				x			
39	x		x											
40	x													
42												x		
46			x				x							
53				x										

56		x			x									
67					x									
69	x	x	x		x				x		x			
72	x													
75											x		x	
76											x			
79	x		x											
81			x											
83	x													
84	x													
86	x							x						
89	x													
91	x						x							
92												x		
95	x													
96				x										
97				x	x									
103										x				
110							x				x			
113	x													
116	x													
118		x									x	x		
119	x		x											
122												x		
123										x			x	
126					x									
134				x										

136	x		x											
142												x		
145	x		x											
148	x													
149				x							x			
154	x													
157												x		
159	x													
162				x										
167	x	x												
169	x						x				x			
174	x													
176												x		
184													x	
188							x							
195													x	
201	x													
207							x							
213		x												
235				x										x
242	x													
244	x													
245	x													
247		x			x									
251	x													
256	x													
265		x			x									

266							x							
267		x												
268		x	x				x		x					
269	x								x					
274														x
284					x									
296	x	x												
313												x		
328					x									
335												x		
343												x		
347	x													
359	x						x				x	x		
360							x							
365					x									
367							x					x		
376		x			x									
378													x	
382			x											
383													x	
384	x													
392	x													
396												x		
397	x											x		
402		x												
404								x						
413	x													

417							x							
422							x							
424								x						
425									x					
427	x		x											
433														x
435												x		
444	x		x											
445					x									
446		x										x		
452												x		
453									x					
456							x							
464														x
465	x													
467	x													
469									x					
470		x										x		
482							x							
501					x								x	
510												x		
511	x													
513							x							
514												x		
518										x				
520	x													
524							x							

525								x						
528							x							
529											x			
531		x			x									
535												x		
539							x							
542							x							
550								x						
553						x								
568					x			x						
571												x		
573											x	x		
575									x					
586												x		
588							x		x					
594					x									
596												x		
607														x
609														x
611											x	x		
613													x	
614											x	x		
618												x		
622							x							
626														x
630	x													
645						x								

655							x							
656							x							
658											x			
664											x			
665							x							
666												x		
667							x				x			
676	x													
677							x							
681	x													
682	x													
689	x	x					x							
694							x							
699												x		
700														x
701	x													
711												x		
716											x	x		
717													x	
719							x							
720							x							
722							x							
723							x							
732								x						
739							x							
740							x						x	
745							x							

751							x							
755							x							
773	x											x		
776	x													
777							x							
778	x													
781		x												
791												x		
796		x										x		
797	x													
804				x										
813													x	
821												x		
834							x							
M3		x												
M5	x		x											
M6				x										
M8				x										
M9		x												
M10				x										
M11			x											
M13									x					
E2		x												
E3							x							
E11	x	x	x				x							
E15	x	x	x											
E24		x												

E31	x						x							
E33	x		x											
E37	x													
E61												x		
E65	x													

S.5 *Classification of estimation methods*

Table S3. Classification of estimation methods (according to Section 5).
A: KM based estimation approaches (S.2.1); B: stratified Cox (S.2.2); C.1: change point for time varying effect (S.2.3.1); C.2: other time covariate interaction for time-varying coefficients (S.2.3.2); C.3 fractional polynomials (S.2.3.3); C.4 splines (S.2.3.4); C.5: aHR and summary effect measures obtained through weighted partial likelihood (S.2.3.5); C.6: additive model (S.2.3.6); D: joint models (S.2.5); E: Short- and long-term HR (S.2.6); F: transformation models with time-covariate interaction (S.2.4); G: frailty models (S.2.7); H.1: piecewise exponential hazard (S.2.8.1); H.2: AFT and GAMLSS (S.2.8.2); H.3: FHT (S.2.8.3); H.4: GLMs and other parametric approaches (S.2.8.4); I.1 trees and forests (S.2.9); I.2 neural networks (S.2.9); I.3 kernel smoothing (S.2.9); I.4 other machine learning (S.2.9); J: other modelling approaches (S.2.10); K: Bayesian fitting approach

ID	A	B	C.1	C.2	C.3	C.4	C.5	C.6	D	E	F	G	H.1	H.2	H.3	H.4	I.1	I.2	I.3	I.4	J	K
**14**		x			x						x		x	x								
**16**	x																					
**20**			x																			x
**21**											x											
**26**	x																					
**27**				x	x	x																
**30**			x									x										
**42**																x						
**46**			x	x																		
**56**	x																					
**67**	x																					

**69**	x									x		x										
**75**												x					x					x
**76**												x										
**86**											x											
**91**							x															
**92**															x							x
**97**	x																					
**103**									x				x									x
**110**			x									x										
**118**												x				x						
**122**													x			x						
**123**									x								x					
**126**	x																					
**142**														x								
**149**												x										
**157**														x								
**169**			x									x										
**176**																x						
**184**																			x			
**188**								x														
**195**																	x					
**207**					x																	
**235**																					x	
**247**	x																					
**265**	x																					
**266**							x															
**268**							x			x												

**269**										x												
**274**																					x	
**284**	x																					
**313**														x								
**328**	x																					
**335**													x									x
**343**														x								
**359**			x									x		x								
**360**						x																x
**365**	x																					
**367**				x										x								
**376**	x																					
**378**																				x		
**383**																	x					x
**396**														x								
**397**														x								
**404**											x											
**417**						x																
**422**						x																
**424**											x											
**425**										x												
**433**																					x	
**435**															x							
**445**	x																					
**446**													x									
**452**													x									
**453**										x												

**456**						x																
**464**																					x	
**469**										x												
**470**													x									
**482**						x																
**501**	x																			x		
**510**																x						
**513**								x														
**514**																x						
**518**									x													x
**524**								x														
**525**											x											
**528**				x																		
**529**												x										
**531**	x																					
**535**																x						
**539**							x															
**542**							x															
**550**						x																
**553**		x																				
**568**	x										x											
**571**														x								
**573**												x		x								x
**575**										x												
**586**													x									
**588**							x			x												
**594**	x																					

**596**														x								
**607**																					x	
**609**																					x	
**611**												x		x								
**613**																			x			
**614**												x			x							x
**618**															x							
**622**							x															
**626**																					x	x
**645**		x																				
**655**					x																	
**656**						x																
**658**												x										x
**664**												x										x
**665**			x																			
**666**														x								
**667**				x	x	x						x										
**677**				x																		
**689**							x															
**694**								x														x
**699**																x						
**700**																					x	
**711**														x								
**716**												x		x								
**717**																		x				x
**719**						x																
**720**							x															

**722**						x																
**723**					x																	
**732**											x											
**739**						x																
**740**			x														x					
**745**			x																			
**751**			x																			
**755**			x																			x
**773**														x								
**777**				x																		x
**791**																x						
**796**													x									
**813**																		x				
**821**														x								
**834**				x																		
**M13**						x				x												
**E3**							x															
**E11**							x															
**E31**							x															
**E61**																x						

S.6 Classification of articles including hypothesis tests

Table S4. Classification of articles including hypothesis tests including the definition of the null hypothesis.

	**Hypothesis Tests**								
ID	Log-rank tests	Kaplan-Meier based tests	Combination tests	Other tests	$S_1(t) = S_2(t)$	$S_1(t) \leq S_2(t)$	Other average based null hypothesis	Other/Not explicitly mentioned	Approach for sample size calculation
1	x					x			x
9	x	x	x	x	x		x (RMST)		
19			x			x			
22	x	x			x		x(milestone survival)		x
28			x		x				x
30	x				x				x
39	x		x		x				
40	x				x				
46			x				x(regression coefficient)		
53				x			x(relative time)		x
56		x					x(window mean survival time)		
69	x	x	x		x		x(RMST)		x
72	x				x				x
79	x		x				x(HR)		x
81			x				x(HR)		x
83	x						x(HR)		x
84	x						x(HR)		

86	x				x				
89	x				x				x
91	x						x(HR)		
95	x						x(HR)		x
96				x		x			x
97				x			x(Survival median)		
113	x						x(HR non inferiority)		x
116	x				x				x
118		x					x(RMST)		
119	x		x			x			
134				x				x	
136	x		x		x				
145	x		x		x				
148	x				x				
149				x				x	
154	x				x				x
159	x				x				x
162				x	x				
167	x	x			x				x
169	x				x				x
174	x				x				x
201	x					x			
213	x	x					x(RMST)		
235				x	x				
242	x				x				

244	x				x				
245	x						x(HR)		
247		x			x				
251	x				x				x
256	x				x				x
265		x					x(aHR non inferiority)		
267		x					x(RMST)		
268		x	x		x				
269	x				x				
296	x	x					x(HR)		x
347	x				x				
359	x				x				
376		x					x(aHR)		
382			x		x				
384	x				x				x
392	x				x				
397	x				x				
402		x			x				
413	x						x(parameter)		
427	x		x		x				
444	x		x				x(HR)		
446		x					x(RMST)		
465	x				x				x
467	x				x				
470		x					x(RMST)		x
511	x				x				

520	x				x				
531		x			x				
630	x				x				x
676	x						x(HR non inferiority)		
681	x					x			x
682	x				x				
689	x	x			x				
701	x				x				x
773	x				x			x	
776	x				x				x
778	x						x(regression coefficient and HR)		
781		x			x				
796		x					x(RMST)		
797	x				x				
804				x	x				
M3		x			x				
M5	x		x		x				
M6				x	x				
M8				x	x				
M9		x			x				
M10				x	x				
M11			x		x				
E2		x			x				x
E11	x	x	x		x		x(RMST)		

E15	x	x	x		x		x(RMST)		
E24		x					x(RMST)		
E31	x						x(HR)		
E33	x		x		x				
E37	x						x(HR)		x
E65	x				x				

S.7 Bibliographic information for all articles included in our systematic review

Table S5. Bibliographic information for all articles included in our systematic review. IDs correspond to the original set of 907 articles screened during the review. IDs without any letters are the articles identified by the PUBMED search. The letter "M" indicates the results of the manual search and "E" the identified articles of the EMBASE search.

ID	DOI	Title	First Author	Source	Year
1	10.1177/17407745211072848	Design and analysis of group-sequential clinical trials based on a modestly weighted log-rank test in anticipation of a delayed separation of survival curves: A practical guidance	Magirr D	Clin Trials	2022
9	10.1186/s12874-022-01520-0	Which test for crossing survival curves? A user's guideline	Dormuth I	BMC Med Res Methodol	2022
14	10.1177/09622802211070253	Challenges of modelling approaches for network meta-analysis of time-to-event outcomes in the presence of non-proportional hazards to aid decision making: Application to a melanoma network	Freeman SC	Stat Methods Med Res	2022
16	10.1200/PO.20.00164	Insights for Quantifying the Long-Term Benefit of Immunotherapy Using Quantile Regression	Mboup B	JCO Precis Oncol	2021
19	10.1002/bimj.202000169	Robust group sequential designs for trials with survival endpoints and delayed response	Ghosh P	Biom J	2022

20	10.1016/j .ejca.2021.11.002	Bayesian interpretation of immunotherapy trials with dynamic treatment effects	Castañon E	Eur J Cancer	2022
21	10.1001/-jamanetworkopen.2021.39573	Assessment of Treatment Effects and Long-term Benefits in Immune Checkpoint Inhibitor Trials Using the Flexible Parametric Cure Model: A Systematic Review	Filleron T	JAMA Netw Open	2021
22	10.1002/sim.9256	Complex survival trial design by the product integration method	Tang Y	Stat Med	2022
26	10.1177/09622802211041756	Weighted pseudo-values for partly unobserved group membership in paediatric stem cell transplantation studies	Mittlböck M	Stat Methods Med Res	2022
27	10.1002/sim.9259	Using fractional polynomials and restricted cubic splines to model non-proportional hazards or time-varying covariate effects in the Cox regression model	Austin PC	Stat Med	2022
28	10.1002/sim.9251	M&M: A maximum duration design with the Maxcombo test for a group sequential trial of an immunotherapy with a random delayed treatment effect	Li B	Stat Med	2022
30	10.1002/sim.9258	Cancer immunotherapy trial design with random delayed treatment effect and cure rate	Wu J	Stat Med	2022
39	10.1111/biom.13575	CASANOVA: Permutation inference in factorial survival designs	Ditzhaus M	Biometrics	2021

40	10.1080/10543406.2021 .1979575	Multi-arm multi-stage clinical trials for time-to-event outcomes	Dixit V	J Biopharm Stat	2021
42	10.1002/bimj.202000292	Nonproportional hazards model with a frailty term for modeling subgroups with evidence of long-term survivors: Application to a lung cancer dataset	Gazon AB	Biom J	2022
46	10.1177/09622802211037076	CauchyCP: A powerful test under non-proportional hazards using Cauchy combination of change-point Cox regressions	Zhang H	Stat Methods Med Res	2021
53	10.1002/bimj.202000043	Sample size calculation for two-arm trials with time-to-event endpoint for nonproportional hazards using the concept of Relative Time when inference is built on comparing Weibull distributions	Phadnis MA	Biom J	2021
56	10.1002/sim.9138	Window mean survival time	Paukner M	Stat Med	2021
67	10.1016/j .cmpb.2021.106155	Dynamic prediction and analysis based on restricted mean survival time in survival analysis with nonproportional hazards	Yang Z	Comput Methods Programs Biomed	2021
69	10.1016/j .critrevonc.2021.-103350	Critical review of oncology clinical trial design under non-proportional hazards	Anantha-krishnan R	Crit Rev Oncol Hematol	2021
72	10.1186/s12874-021-01286-x	Using the geometric average hazard ratio in sample size calculation for time-to-event data with composite endpoints	Cortés Martínez J	BMC Med Res Methodol	2021

75	10.1111/biom.13478	Semiparametric analysis of clustered interval-censored survival data using soft Bayesian additive regression trees (SBART)	Basak P	Biometrics	2021
76	10.1001/jamaoncol.2021.0289	Development and Evaluation of a Method to Correct Misinterpretation of Clinical Trial Results With Long-term Survival	Hsu CY	JAMA Oncol	2021
79	10.1177/0962280220988570	A unified approach to power and sample size determination for log-rank tests under proportional and nonproportional hazards	Tang Y	Stat Methods Med Res	2021
81	10.1002/pst.2116	A simulation-free group sequential design with max-combo tests in the presence of non-proportional hazards	Wang L	Pharm Stat	2021
83	10.1080/10543406.2020 .1815035	Statistical and practical considerations in designing of immuno-oncology trials	Mukho-padhyay P	J Biopharm Stat	2020
84	10.1002/sim.8905	On information fraction for Fleming-Harrington type weighted log-rank tests in a group-sequential clinical trial design	Kundu MG	Stat Med	2021
86	10.1016/j .esmoop.2020 .100043	Cediranib in addition to chemotherapy for women with relapsed platinum-sensitive ovarian cancer (ICON6): overall survival results of a phase III randomised trial	Ledermann JA	ESMO Open	2021

89	10.1007/s10985-021-09517-5	Testing equivalence of survival before but not after end of follow-up	Furberg JK	Lifetime Data Anal	2021
91	10.1002/pst.2092	A weighted log-rank test and associated effect estimator for cancer trials with delayed treatment effect	Yu C	Pharm Stat	2021
92	10.1007/s10985-020-09514-0	Semi-parametric survival analysis via Dirichlet process mixtures of the First Hitting Time model	Race JA	Lifetime Data Anal	2021
95	10.1177/0962280220980780	A group sequential design and sample size estimation for an immunotherapy trial with a delayed treatment effect	Li B	Stat Methods Med Res	2021
96	10.1002/pst.2091	Non-proportional hazards in immuno-oncology: Is an old perspective needed?	Magirr D	Pharm Stat	2021
97	10.1177/0962280220980784	Inferring median survival differences in general factorial designs via permutation tests	Ditzhaus M	Stat Methods Med Res	2021
103	10.1093/biostatistics/kxaa044	Bayesian design of clinical trials using joint models for longitudinal and time-to-event data	Xu J	Biostatistics	2020
110	10.1002/sim.8694	Design for immuno-oncology clinical trials enrolling both responders and nonresponders	Xu Z	Stat Med	2020
113	10.1002/pst.2069	Sample size calculation for log-rank test and prediction of number of events over time	Lu K	Pharm Stat	2021
116	10.1002/pst.2060	Cancer immunotherapy trial design with long-term survivors	Ding X	Pharm Stat	2021

118	10.1186/s12874-020-01098-5	Dynamic RMST curves for survival analysis in clinical trials	Liao JJZ	BMC Med Res Methodol	2020
119	10.1002/pst.2062	Delayed treatment effects, treatment switching and heterogeneous patient populations: How to design and analyze RCTs in oncology	Ristl R	Pharm Stat	2021
122	10.1016/j .jval.2020.03.010	Nonproportional Hazards in Network Meta-Analysis: Efficient Strategies for Model Building and Analysis	Wiksten A	Value Health	2020
123	10.1177/0962280220941532	Functional survival forests for multivariate longitudinal outcomes: Dynamic prediction of Alzheimer's disease progression	Lin J	Stat Methods Med Res	2021
126	10.1136/bmjopen-2019-033965	Moving beyond the Cox proportional hazards model in survival data analysis: a cervical cancer study	Li L	BMJ Open	2020
134	10.1111/biom.13315	Optimality of testing procedures for survival data in the nonproportional hazards setting	Arfè A	Biometrics	2021
136	10.1177/0962280220931560	Group sequential monitoring based on the maximum of weighted log-rank statistics with the Fleming-Harrington class of weights in oncology clinical trials	Prior TJ	Stat Methods Med Res	2020
142	10.1002/sim.8508	A multiparameter regression model for interval-censored survival data	Peng D	Stat Med	2020

145	10.1186/s13063-020-4153-2	A simulation study comparing the power of nine tests of the treatment effect in randomized controlled trials with a time-to-event outcome	Royston P	Trials	2020
148	10.1177/0962280220907355	K-sample omnibus non-proportional hazards tests based on right-censored data	Gorfine M	Stat Methods Med Res	2020
149	10.1186/s13045-020-0847-x	Treating non-responders: pitfalls and implications for cancer immunotherapy trial design	Xu Z	J Hematol Oncol	2020
154	10.1002/pst.2003	Designing cancer immunotherapy trials with delayed treatment effect using maximin efficiency robust statistics	Ding X	Pharm Stat	2020
157	10.1109/TCBB.2020.2965934	"Supervised Dimension Reduction for Large-Scale ""Omics"" Data With Censored Survival Outcomes Under Possible Non-Proportional Hazards"	Spirko-Burns L	IEEE/ACM Trans Comput Biol Bioinform	2021
159	10.1002/pst.1976	Study design of single-arm phase II immunotherapy trials with long-term survivors and random delayed treatment effect	Chu C	Pharm Stat	2020
162	10.1002/pst.2000	A resampling-based test for two crossing survival curves	Liu T	Pharm Stat	2020
167	10.1111/biom.13196	Sample size and power for the weighted log-rank test and Kaplan-Meier based tests with allowance for nonproportional hazards	Yung G	Biometrics	2020

169	10.1002/sim.8440	Cancer immunotherapy trial design with cure rate and delayed treatment effect	Wei J	Stat Med	2020
174	10.1002/pst.1982	Cancer immunotherapy trial design with delayed treatment effect	Wu J	Pharm Stat	2020
176	10.1177/0962280219883905	Long-term frailty modeling using a non-proportional hazards model: Application with a melanoma dataset	Calsavara VF	Stat Methods Med Res	2020
184	10.1002/sim.8363	Quantifying treatment effects using the personalized chance of longer survival	Zhao YQ	Stat Med	2019
188	10.3389/fpubh.2019.00201	Modeling Long-Term Graft Survival With Time-Varying Covariate Effects: An Application to a Single Kidney Transplant Centre in Johannesburg, South Africa	Achilonu OJ	Front Public Health	2019
195	10.1177/0962280219862586	Survival forests under test: Impact of the proportional hazards assumption on prognostic and predictive forests for amyotrophic lateral sclerosis survival	Korepanova N	Stat Methods Med Res	2020
201	10.1002/sim.8186	Modestly weighted log-rank tests	Magirr D	Stat Med	2019
207	10.1016/j.jamcollsurg.2019.04.019	A Tale of Two Kidneys: Differences in Graft Survival for Kidneys Allocated to Simultaneous Liver Kidney Transplant Compared with Contralateral Kidney from the Same Donor	Cannon RM	J Am Coll Surg	2019
213	10.1186/s13063-019-3251-5	Combined test versus log-rank/Cox test in 50 randomised trials	Royston P	Trials	2019

235	10.1111/biom.13007	Nonparametric group sequential methods for evaluating survival benefit from multiple short-term follow-up windows	Xia M	Biometrics	2019
242	10.1002/sim.7975	Design and monitoring of survival trials in complex scenarios	Luo X	Stat Med	2019
244	10.1002/sim.7958	Interim monitoring using the adaptively weighted log-rank test in clinical trials for survival outcomes	Yang S	Stat Med	2019
245	10.1002/sim.7937	Designing cancer immunotherapy trials with random treatment time-lag effect	Xu Z	Stat Med	2018
247	10.1002/sim.7936	Nonparametric adaptive enrichment designs using categorical surrogate data	Brückner M	Stat Med	2018
251	10.1002/pst.1878	Weighted log-rank test for time-to-event data in immunotherapy trials with random delayed treatment effect and cure rate	Liu S	Pharm Stat	2018
256	10.1111/biom.12916	A robust approach to sample size calculation in cancer immunotherapy trials with delayed treatment effect	Ye T	Biometrics	2018
265	10.3414/ME17-01-0058	The Average Hazard Ratio - A Good Effect Measure for Time-to-event Endpoints when the Proportional Hazard Assumption is Violated?	Rauch G	Methods Inf Med	2018

266	10.1016/j .conctc.2017.09.004	Estimation of treatment effects in weighted log-rank tests	Lin RS	Contemp Clin Trials Commun	2017
267	10.1002/sim.7661	A flexible and coherent test/estimation procedure based on restricted mean survival times for censored time-to-event data in randomized clinical trials	Horiguchi M	Stat Med	2018
268	10.1002/sim.7676	Improving testing and description of treatment effect in clinical trials with survival outcomes	Yang S	Stat Med	2019
269	10.1177/1740774518769865	A new modeling and inference approach for the Systolic Blood Pressure Intervention Trial outcomes	Yang S	Clin Trials	2018
274	10.1007/s10985-018-9428-5	Improved precision in the analysis of randomized trials with survival outcomes, without assuming proportional hazards	Díaz I	Lifetime Data Anal	2019
284	10.1186/s12874-017-0430-5	Assessing the effect of a partly unobserved, exogenous, binary time-dependent covariate on survival probabilities using generalised pseudo-values	Pötschger U	BMC Med Res Methodol	2018
296	10.1002/pst.1840	Design considerations in clinical trials with cure rate survival data: A case study in oncology	Sun S	Pharm Stat	2018
313	10.1002/sim.7421	A clinical trial design using the concept of proportional time using the generalized gamma ratio distribution	Phadnis MA	Stat Med	2017

328	10.1136/bmj.j2250	Life expectancy difference and life expectancy ratio: two measures of treatment effects in randomised trials with non-proportional hazards	Dehbi HM	BMJ	2017
335	10.1177/0962280216688034	Flexible modeling of the hazard rate and treatment effects in long-term survival studies	Hagar Y	Stat Methods Med Res	2017
343	10.1111/biom.12625	Multi-parameter regression survival modeling: An alternative to proportional hazards	Burke K	Biometrics	2017
347	10.1002/sim.7157	Designing therapeutic cancer vaccine trials with delayed treatment effect	Xu Z	Stat Med	2017
359	10.1002/pst.1760	Group sequential monitoring based on the weighted log-rank test statistic with the Fleming-Harrington class of weights in cancer vaccine studies	Hasegawa T	Pharm Stat	2016
360	10.1186/s40064-016-2210-9	Bayesian test for hazard ratio in survival analysis	Kim G	Springerplus	2016
365	10.1177/0962280216648724	A censored quantile regression approach for the analysis of time to event data	Xue X	Stat Methods Med Res	2018
367	10.1177/0962280216642264	Assessing methods for dealing with treatment switching in clinical trials: A follow-up simulation study	Latimer NR	Stat Methods Med Res	2018
376	10.1007/s10985-016-9360-5	Sequential tests for non-proportional hazards data	Brückner M	Lifetime Data Anal	2017
378	10.1002/sim.6929	Estimating restricted mean treatment effects with stacked survival models	Wey A	Stat Med	2016

382	10.1186/s12874-016-0110-x	Augmenting the logrank test in the design of clinical trials in which non-proportional hazards of the treatment effect may be anticipated	Royston P	BMC Med Res Methodol	2016
383	10.1002/sim.6893	Nonparametric survival analysis using Bayesian Additive Regression Trees (BART)	Sparapani RA	Stat Med	2016
384	10.1002/sim.6889	Design and analysis of clinical trials in the presence of delayed treatment effect	Sit T	Stat Med	2016
392	10.1002/sim.6801	Gaining power and precision by using model-based weights in the analysis of late stage cancer trials with substantial treatment switching	Bowden J	Stat Med	2016
396	10.1177/0962280215605107	An ensemble survival model for estimating relative residual longevity following stroke: Application to mortality data in the chronic dialysis population	Phadnis MA	Stat Methods Med Res	2017
397	10.1080/10543406.2015.-1094809	Interim analysis based on the weighted log-rank test for delayed treatment effects under staggered patient entry	Yoshida M	J Biopharm Stat	2016
402	10.1002/sim.6591	A versatile test for equality of two survival functions based on weighted differences of Kaplan-Meier curves	Uno H	Stat Med	2015
404	10.1016/S1470-2045(15)00086-8	Standard chemotherapy with or without bevacizumab for women with newly diagnosed ovarian cancer (ICON7): overall survival results of a phase 3 randomised trial	Oza AM	Lancet Oncol	2015

413	10.1016/j .cct.2015.05.018	Design of clinical trials with failure-time endpoints and interim analyses: An update after fifteen years	He P	Contemp Clin Trials	2015
417	10.1371/journal.-pone.0123784	Analysis of time to event outcomes in randomized controlled trials by generalized additive models	Argyro-poulos C	PLoS One	2015
422	10.1111/tri.12568	Post-transplant survival is improved for hepatitis C recipients who are RNA negative at time of liver transplantation	Fortune BE	Transpl Int	2015
424	10.1515/ijb-2014-0006	Conditional transformation models for survivor function estimation	Möst L	Int J Biostat	2015
425	10.1002/sim.6453	Assessing potentially time-dependent treatment effect from clinical trials and observational studies for survival data, with applications to the Women's Health Initiative combined hormone therapy trial	Yang S	Stat Med	2015
427	10.1371/journal.pone .0116774	Statistical inference methods for two crossing survival curves: a comparison of methods	Li H	PLoS One	2015
433	10.1177/0962280214557578	Adjusting for treatment switching in randomised controlled trials - A simulation study and a simplified two-stage method	Latimer NR	Stat Methods Med Res	2017
435	10.1002/sim.6356	A model for time to fracture with a shock stream superimposed on progressive degradation: the Study of Osteoporotic Fractures	He X	Stat Med	2015

Prepared using sagej.cls

444	10.1186/1745-6215-15-314	An approach to trial design and analysis in the era of non-proportional hazards of the treatment effect	Royston P	Trials	2014
445	10.1007/s10985-014-9301-0	Quantifying the average of the time-varying hazard ratio via a class of transformations	Chen Q	Lifetime Data Anal	2015
446	10.1200/JCO.2014.55.2208	Moving beyond the hazard ratio in quantifying the between-group difference in survival analysis	Uno H	J Clin Oncol	2014
452	10.1016/S1470-2045(14)70189-5	Ipilimumab versus placebo after radiotherapy in patients with metastatic castration-resistant prostate cancer that had progressed after docetaxel chemotherapy (CA184-043): a multicentre, randomised, double-blind, phase 3 trial	Kwon ED	Lancet Oncol	2014
453	10.1016/j .spl.2013.03.007	On penalized likelihood estimation for a non-proportional hazards regression model	Devarajan K	Stat Probab Lett	2013
456	10.1002/sim.6178	Impact of the model-building strategy on inference about nonlinear and time-dependent covariate effects in survival analysis	Wynant W	Stat Med	2014
464	10.1002/sim.6105	Likelihood approaches for proportional likelihood ratio model with right-censored data	Zhu H	Stat Med	2014

465	10.1002/pst.1609	Sample size determination for the weighted log-rank test with the Fleming-Harrington class of weights in cancer vaccine studies	Hasegawa T	Pharm Stat	2014
467	10.1080/07474946.2014 .856635	Flexibly Monitoring Group Sequential Survival Trials When Testing is Based Upon a Weighted Log-Rank Statistic	Brummel SS	Seq Anal	2014
469	10.1111/biom.12097	Efficient semiparametric estimation of short-term and long-term hazard ratios with right-censored data	Diao G	Biometrics	2013
470	10.1186/1471-2288-13-152	Restricted mean survival time: an alternative to the hazard ratio for the design and analysis of randomized trials with a time-to-event outcome	Royston P	BMC Med Res Methodol	2013
482	10.1002/sim.5961	Model building in nonproportional hazard regression	Rodríguez-Girondo M	Stat Med	2013
501	10.1002/sim.5673	A K-nearest neighbors survival probability prediction method	Lowsky DJ	Stat Med	2013
510	10.1080/10543406.2011 .616973	A threshold hazard model for estimating serious infection risk following anti-tumor necrosis factor therapy in rheumatoid arthritis patients	Fu B	J Biopharm Stat	2013
511	10.1080/10543406.2011.-616975	Power comparisons for group sequential tests with nonparametric statistics in case of nonproportional hazards	Demirhan H	J Biopharm Stat	2013

513	10.1155/2013/796270	Additive hazard regression models: an application to the natural history of human papillomavirus	Xie X	Comput Math Methods Med	2013
514	10.1111/biom.12002	Estimating time to disease progression comparing transition models and survival methods–an analysis of multiple sclerosis data	Mandel M	Biometrics	2013
518	10.1177/0962280212473348	Estimating efficacy in the presence of non-ignorable non-trial interventions in the Helsinki Psychotherapy Study	Härkänen T	Stat Methods Med Res	2016
520	10.1186/2051-1426-1-18	Statistical issues and challenges in immuno-oncology	Chen TT	J Immunother Cancer	2013
524	10.1007/s10985-012-9234-4	Estimation of odds of concordance based on the Aalen additive model	Martinussen T	Lifetime Data Anal	2013
525	10.1371/journal.pone .0047804	A flexible alternative to the Cox proportional hazards model for assessing the prognostic accuracy of hospice patient survival	Miladinovic B	PLoS One	2012
528	10.1186/1471-2369-13-130	All-cause and cause-specific mortality associated with diabetes in prevalent hemodialysis patients	Sattar A	BMC Nephrol	2012
529	10.1111/j.1541-0420.2012.01784.x	A general class of semiparametric transformation frailty models for non-proportional hazards survival data	Choi S	Biometrics	2012

531	10.1177/1740774512455464	Utilizing the integrated difference of two survival functions to quantify the treatment contrast for designing, monitoring, and analyzing a comparative clinical study	Zhao L	Clin Trials	2012
535	10.1007/s10985-012-9224-6	Robust inference in discrete hazard models for randomized clinical trials	Nguyen VQ	Lifetime Data Anal	2012
539	10.1002/sim.5440	Estimation of treatment effect under non-proportional hazards and conditionally independent censoring	Boyd AP	Stat Med	2012
542	10.1007/s10985-012-9222-8	Estimation of treatment effects based on possibly misspecified Cox regression	Hattori S	Lifetime Data Anal	2012
550	10.1056/NEJMoa1103799	A phase 3 trial of bevacizumab in ovarian cancer	Perren TJ	N Engl J Med	2011
553	10.1111/j.1541-0420.2011.01696.x	Empirical likelihood for cumulative hazard ratio estimation with covariate adjustment	Dong B	Biometrics	2012
568	10.1002/sim.4274	The use of restricted mean survival time to estimate the treatment effect in randomized clinical trials when the proportional hazards assumption is in doubt	Royston P	Stat Med	2011
571	10.1007/s00405-011-1601-3	A critical appraisal of different survival techniques in oral cancer patients	Köhler HF	Eur Arch Otorhinolaryngol	2012

573	10.1080/02664760903521476	Bayesian Parametric Accelerated Failure Time Spatial Model and its Application to Prostate Cancer	Zhang J	J Appl Stat	2011
575	10.1007/s10985-011-9195-z	Efficiency improvement in a class of survival models through model-free covariate incorporation	Garcia TP	Lifetime Data Anal	2011
586	10.1001/archinternmed.2010 .352	Similar outcomes with hemodialysis and peritoneal dialysis in patients with end-stage renal disease	Mehrotra R	Arch Intern Med	2011
588	10.1093/biostatistics/kxq061	Estimation of the 2-sample hazard ratio function using a semiparametric model	Yang S	Biostatistics	2011
594	10.1111/j.1541-0420.2010.01449.x	Double inverse-weighted estimation of cumulative treatment effects under nonproportional hazards and dependent censoring	Schaubel DE	Biometrics	2011
596	10.1111/j.1365-2753.2009.01190.x	SAS macros for point and interval estimation of area under the receiver operating characteristic curve for non-proportional and proportional hazards Weibull models	Mannan H	J Eval Clin Pract	2010
607	10.1093/bioinformatics/ btq035	Gene selection in microarray survival studies under possibly non-proportional hazards	Dunkler D	Bioinformatics	2010
609		Estimating a scale-change effect for time-varying phenotypes in genome-wide association studies	Chen YQ	J Appl Statist Sci	2010
611	10.1002/bimj.200800244	An accelerated failure time mixture cure model with masked event	Zhang JJ	Biom J	2009

613	10.1007/s10985-009-9132-6	Confidence intervals for the first crossing point of two hazard functions	Cheng MY	Lifetime Data Anal	2009
614	10.1093/biostatistics/kxp041	Bayesian random-effects threshold regression with application to survival data with nonproportional hazards	Pennell ML	Biostatistics	2010
618	10.1002/sim.3686	A semi-parametric threshold regression analysis of sexually transmitted infections in adolescent women	Yu Z	Stat Med	2009
622	10.1002/sim.3623	The estimation of average hazard ratios by weighted Cox regression	Schemper M	Stat Med	2009
626	10.1111/j.1541-0420.2008.01166.x	Bayesian nonparametric nonproportional hazards survival modeling	De Iorio M	Biometrics	2009
630	10.1002/sim.3501	Power and sample size calculation for log-rank test with a time lag in treatment effect	Zhang D	Stat Med	2009
645	10.1111/j.1541-0420.2007.00947.x	Estimating cumulative treatment effects in the presence of nonproportional hazards	Wei G	Biometrics	2008
655	10.1002/bimj.200610328	A new proposal for multivariable modelling of time-varying effects in survival data based on fractional polynomial time-transformation	Sauerbrei W	Biom J	2007
656	10.1002/bimj.200510325	A partial likelihood approach to smooth estimation of dynamic covariate effects using penalised splines	Brown D	Biom J	2007

658	10.1007/s10985-007-9035-3	Bayesian analysis of generalized odds-rate hazards models for survival data	Banerjee T	Lifetime Data Anal	2007
664	10.1111/j.1541-0420.2006.00571.x	Bayesian semiparametric dynamic frailty models for multiple event time data	Pennell ML	Biometrics	2006
665	10.1534/genetics.106.059808	Mapping temporally varying quantitative trait loci in time-to-failure experiments	Johannes F	Genetics	2007
666	10.1002/pst .213	Comparing proportional hazards and accelerated failure time models: an application in influenza	Patel K	Pharm Stat	2006
667	10.1002/sim.2729	Approaches in modelling long-term survival: an application to breast cancer	Perperoglou A	Stat Med	2007
676	10.1080/10543400600614791	Testing for crossover of two hazard functions using Gail and Simon's method	Chen YH	J Biopharm Stat	2006
677	10.1007/s10549-006-9231-y	Effects of estrogen receptor expression and histopathology on annual hazard rates of death from breast cancer	Anderson WF	Breast Cancer Res Treat	2006
681	10.1002/sim.2517	Evaluation of sample size and power for multi-arm survival trials allowing for non-uniform accrual, non-proportional hazards, loss to follow-up and cross-over	Barthel FM	Stat Med	2006
682	10.1002/gepi.20132	Family-based association test for time-to-onset data with time-dependent differences between the hazard functions	Jiang H	Genet Epidemiol	2006

689	10.1002/sim.2248	Interim analysis on survival data: its potential bias and how to repair it	van Houwelingen HC	Stat Med	2005
694	10.1007/s10985-004-0384-x	Bayesian model selection and averaging in additive and proportional hazards models	Dunson DB	Lifetime Data Anal	2005
699	10.1016/j .compbiolchem.-2005.02.001	Survival analysis of microarray expression data by transformation models	Xu J	Comput Biol Chem	2005
700	10.1111/j.0006-341X.2005.030814.x	Survival model predictive accuracy and ROC curves	Heagerty PJ	Biometrics	2005
701	10.1111/j.0006-341X.2005.031206.x	A sample size formula for the supremum log-rank statistic	Eng KH	Biometrics	2005
711	10.1023/b: lida.0000019255.21735.9b	Incorporating follow-up time in M-estimation for survival data	Heller G	Lifetime Data Anal	2004
716	10.1002/sim.1618	Parametric randomization-based methods for correcting for treatment changes in the assessment of the causal effect of treatment	Walker AS	Stat Med	2004
717		The effect of sample size and MLP architecture on Bayesian learning for cancer prognosis–a case study	Trinh QA	Stud Health Technol Inform	2003
719	10.1002/sim.1484	A relative survival regression model using B-spline functions to model non-proportional hazards	Giorgi R	Stat Med	2003
720	10.1093/biostatistics/1.4.423	Estimating average regression effect under non-proportional hazards	Xu R	Biostatistics	2000

722		Assessing time-by-covariate interactions in relative survival models using restrictive cubic spline functions	Bolard P	J Cancer Epidemiol Prev	2002
723	10.1002/sim.1411	Dynamic Cox modelling based on fractional polynomials: time-variations in gastric cancer prognosis	Berger U	Stat Med	2003
732	10.1002/sim.1203	Flexible parametric proportional-hazards and proportional-odds models for censored survival data, with application to prognostic modelling and estimation of treatment effects	Royston P	Stat Med	2002
739	10.1111/j.0006-341x.2002.00443.x	Generalized additive models with interval-censored data and time-varying covariates: application to human immunodeficiency virus infection in hemophiliacs	Bacchetti P	Biometrics	2002
740	10.1111/j.0006-341x.2002.00305.x	Survival analysis with time-varying regression effects using a tree-based approach	Xu R	Biometrics	2002
745	10.1002/sim.1061	Estimation of the survival function for Gray's piecewise-constant time-varying coefficients model	Valenta Z	Stat Med	2002
751	10.1016/s0895-4356(01)00363-8	Modelling time-dependent hazard ratios in relative survival: application to colon cancer	Bolard P	J Clin Epidemiol	2001
755	10.1111/j.0006-341x.1999.00585.x	Bayesian analysis and model selection for interval-censored survival data	Sinha D	Biometrics	1999

Prepared using sagej.cls

773	10.1002/(sici)1097-0258(19991015)18:19 <2617::aid-sim187>3.0.co;2-e"	Randomization-based methods for correcting for treatment changes: examples from the Concorde trial	White IR	Stat Med	1999
776	10.1002/(sici)1097-0258(19981115)17:21<2525-::aid-sim936>3.0.co;2-e"	Sample size determination in complex clinical trials comparing more than two groups for survival endpoints	Ahnn S	Stat Med	1998
777	10.1023/a:1009673932333	Flexible Bayesian modelling for survival data	Gustafson P	Lifetime Data Anal	1998
778	10.1023/a:1009689630516	Frailty models and rank tests	Oakes D	Lifetime Data Anal	1998
781		A method for sequential analysis of survival data with nonproportional hazards	Sooriyarachchi MR	Biometrics	1998
791	10.1002/(sici)1097-0258(19970830)16:16<1831-::aid-sim617>3.0.co;2-m	On a non-proportional hazards regression model for repeated medical random counts	Mackenzie G	Stat Med	1997
796	10.1016/s0197-2456(96)00089-x	Use of Irwin's restricted mean as an index for comparing survival in different treatment groups–interpretation and power considerations	Karrison TG	Control Clin Trials	1997
797	10.1002/(sici)1097-0258(19970330)16:6<695-::aid-sim436>3.0.co;2-c	A partially grouped log-rank test	Sposto R	Stat Med	1997
804		Some permutation tests for survival data	Sun Y	Biometrics	1996

Prepared using sagej.cls

813	10.1016/0304-3835(94)90095-7	A technique for using neural network analysis to perform survival analysis of censored data	De Laurentiis M	Cancer Lett	1994
821		A nonproportional hazards Weibull accelerated failure time regression model	Anderson KM	Biometrics	1991
834		Survival analysis of drug combinations using a hazards model with time-dependent covariates	Stablein DM	Biometrics	1980
M3	10.1111/j.0006-341X.2001.00837.x	Maximum of the Weighted Kaplan-Meier Tests with Application to Cancer Prevention and Screening Trials	Shen Y	Biometrics	2001
M5	10.1093/biomet/64.1.156	On distribution-free tests for equality of survival distributions	Tarone RE	Biometrika	1977
M6	10.1002/bimj.200310053	A new testing approach for comparing the overall homogeneity of survival curves	Lin X	Biometrical	2004
M8	10.2307/2556114	Modified Kolmogorov-Smirnov test procedures with application to arbitrarily right-censored data	Fleming TR	Biometrics	1980
M9	10.1002/pst.376	A new method for the comparison of survival distributions	Lin X	Pharm Stat 9(1): 67–76	2010
M10		A two-stage procedure for comparing hazard rate functions	Qiu P	J R Statist Soc B 70(1): 191–208	2008
M11	10.1081/SAC-100107779	Some versatile tests based on the simultaneous use of weighted log-rank and weighted kaplan-meier statistics	Chi Y	Commun Stat 30(4):743–759	2001

M13	10.1093/biomet/92.1.1	Semiparametric analysis of short-term and long-term hazard ratios with two-sample survival data	Yang S	Biometrika 2005; 92: 1–17	2005
E2	10.1007/s12561-0 21-09316-4	Sample Size Re-estimation with the Com-Nougue Method to Evaluate Treatment Effect	Wang J.	Statistics in Biosciences (2022) 14:1 (90-103). Date of Publication: 1 Apr 2022	2022
E3	10.1080/19466315.2020.-1801496	An Assessment of the Treatment Effect in Treatment of Physician Choice Trials	Mpofu P., Karuri S.W.	Statistics in Biopharmaceutical Research (2022) 14:1 (103-113). Date of Publication: 2022	2022
E11	10.1007/s12561-020-09276-1	On Weighted Log-Rank Combination Tests and Companion Cox Model Estimators	León L.F., Lin R., Anderson K.M.	Statistics in Biosciences (2020) 12:2 (225-245). Date of Publication: 1 Jul 2020	2020

E15	10.1080/19466315.2019 .1697738	Alternative Analysis Methods for Time to Event Endpoints Under Nonproportional Hazards: A Comparative Analysis	Lin R.S., Lin J., Roychoudhury S., Anderson K.M., Hu T., Huang B., Leon L.F., Liao J.J.Z., Liu R., Luo X., Mukhopadhyay P., Qin R., Tatsuoka K., Wang X., Wang Y., Zhu J., Chen T.-T., Iacona R.	Statistics in Biopharmaceutical Research (2020) 12:2 (187-198). Date of Publication: 2 Apr 2020	2020
E24	10.1080/19466315.2018 .1527249	Difference in Restricted Mean Survival Time: Small Sample Distribution and Asymptotic Relative Efficiency	Lawrence J., Qiu J., Bai S., Hung H.M.J.	Statistics in Biopharmaceutical Research (2019) 11:1 (61-66). Date of Publication: 2 Jan 2019	2019

E31	10.1016/j.conctc.2017.09.004	Estimation of treatment effects in weighted log-rank tests	Lin R.S., León L.F.	Contemporary Clinical Trials Communications (2017) 8 (147-155). Date of Publication: 1 Dec 2017	2017
E33	10.1080/19466315.2016 .1257436	Testing Treatment Effect in Randomized Clinical Trials With Possible Non-proportional Hazards	Callegaro A., Spiessens B.	Statistics in Biopharmaceutical Research (2017) 9:2 (204-211). Date of Publication: 3 Apr 2017	2017
E37	10.1080/19466315.2016.-1207560	Sample Size and Power of Survival Trials in Group Sequential Design With Delayed Treatment Effect	Zhang J., Pulkstenis E.	Statistics in Biopharmaceutical Research (2016) 8:3 (268-275). Date of Publication: 2 Jul 2016	2016

E61	10.2202/1557-4679.1125	Fitting smooth-in-time prognostic risk functions via logistic regression	Hanley J.A., Miettinen O.S.	International Journal of Biostatistics (2009) 5:1 Article Number: 3. Date of Publication: 2009	2009
E65	10.1177/009286150-704100412	Consequences of delayed treatment effects on analysis of time-to-event endpoints	Fine G.D.	Drug Information Journal (2007) 41:4 (535-539). Date of Publication: 2007	2007

References

[1] Rauch G, Brannath W, Brückner M et al. The average hazard ratio - a good effect measure for time-to-event endpoints when the proportional hazard assumption is violated? *Methods of information in medicine* 2018; 57(3): 89–100. DOI:10.3414/ME17-01-0058.

[2] Schaubel DE and Wei G. Double inverse-weighted estimation of cumulative treatment effects under nonproportional hazards and dependent censoring. *Biometrics* 2011; 67(1): 29–38. DOI:10.1111/j.1541-0420.2010.01449.x. URL https://pubmed.ncbi.nlm.nih.gov/20560935/.

[3] Andersen PK, Hansen MG and Klein JP. Regression analysis of restricted mean survival time based on pseudo-observations. *Lifetime Data Analysis* 2004; 10(4): 335–350. DOI:10.1007/s10985-004-4771-0. URL https://pubmed.ncbi.nlm.nih.gov/15690989/.

[4] Mittlböck M, Pötschger U and Heinzl H. Weighted pseudo-values for partly unobserved group membership in paediatric stem cell transplantation studies. *Statistical methods in medical research* 2022; 31(1): 76–86. DOI:10.1177/09622802211041756.

[5] Yang Z, Wu H, Hou Y et al. Dynamic prediction and analysis based on restricted mean survival time in survival analysis with nonproportional hazards. *Comput Methods Programs Biomed* 2021; 207: 106155. DOI: 10.1016/j.cmpb.2021.106155. URL https://www.sciencedirect.com/science/article/pii/S0169260721002297.

[6] Pötschger U, Heinzl H, Valsecchi MG et al. Assessing the effect of a partly unobserved, exogenous, binary time-dependent covariate on survival probabilities using generalised pseudo-values. *BMC medical research methodology* 2018; 18(1): 14. DOI:10.1186/s12874-017-0430-5.

[7] Li L, Yang Z, Hou Y et al. Moving beyond the cox proportional hazards model in survival data analysis: a cervical cancer study. *BMJ open* 2020; 10(7): e033965. DOI:10.1136/bmjopen-2019-033965.

[8] Klein JP, Gerster M, Andersen PK et al. Sas and r functions to compute pseudo-values for censored data regression. *Computer Methods and Programs in Biomedicine* 2008; 89(3): 289–300. DOI:10.1016/j.cmpb.2007.11.017.

[9] Portnoy S. Censored regression quantiles. *Journal of the American Statistical Association* 2003; 98(464): 1001–1012. URL http://www.jstor.org/stable/30045346.

[10] Peng L and Huang Y. Survival analysis with quantile regression models. *Journal of the American Statistical Association* 2008; 103(482): 637–649. DOI:10.1198/016214508000000355.

[11] Mboup B, Le Tourneau C and Latouche A. Insights for quantifying the long-term benefit of immunotherapy using quantile regression. *JCO precision oncology* 2021; 5: 173–176. DOI:10.1200/PO.20.00164.

[12] Xue X, Xie X and Strickler HD. A censored quantile regression approach for the analysis of time to event data. *Statistical methods in medical research* 2018; 27(3): 955–965. DOI:10.1177/0962280216648724.

[13] Dong B and Matthews DE. Empirical likelihood for cumulative hazard ratio estimation with covariate adjustment. *Biometrics* 2012; 68(2): 408–418. DOI:10.1111/j.1541-0420.2011.01696.x.

[14] Wei G and Schaubel DE. Estimating cumulative treatment effects in the presence of nonproportional hazards. *Biometrics* 2008; 64(3): 724–732. DOI:10.1111/j.1541-0420.2007.00947.x.

[15] Zhang H, Li Q, Mehrotra DV et al. Cauchycp: A powerful test under non-proportional hazards using cauchy combination of change-point cox regressions. *Statistical methods in medical research* 2021; 30(11): 2447–2458. DOI:10.1177/09622802211037076.

[16] Brown D, Kauermann G and Ford I. A partial likelihood approach to smooth estimation of dynamic covariate effects using penalised splines. *Biometrical journal Biometrische Zeitschrift* 2007; 49(3): 441–452. DOI: 10.1002/bimj.200510325.

[17] Sauerbrei W, Royston P and Look M. A new proposal for multivariable modelling of time-varying effects in survival data based on fractional polynomial time-transformation. *Biometrical journal Biometrische Zeitschrift* 2007; 49(3): 453–473. DOI:10.1002/bimj.200610328.

[18] Freeman SC, Cooper NJ, Sutton AJ et al. Challenges of modelling approaches for network meta-analysis of time-to-event outcomes in the presence of non-proportional hazards to aid decision making: Application to a melanoma network. *Statistical methods in medical research* 2022; 31(5): 839–861. DOI:10.1177/09622802211070253.

[19] Argyropoulos C and Unruh ML. Analysis of time to event outcomes in randomized controlled trials by generalized additive models. *PloS one* 2015; 10(4): e0123784. DOI:10.1371/journal.pone.0123784.

[20] Rodríguez-Girondo M, Kneib T, Cadarso-Suárez C et al. Model building in nonproportional hazard regression. *Statistics in medicine* 2013; 32(30): 5301–5314. DOI:10.1002/sim.5961.

[21] Royston P and Parmar MKB. Flexible parametric proportional-hazards and proportional-odds models for censored survival data, with application to prognostic modelling and estimation of treatment effects. *Statistics in medicine* 2002; 21(15): 2175–2197. DOI:10.1002/sim.1203. URL https://pubmed.ncbi.nlm.nih.gov/12210632/.

[22] Johannes F. Mapping temporally varying quantitative trait loci in time-to-failure experiments. *Genetics* 2007; 175(2): 855–865. DOI:10.1534/genetics.106.059808.

[23] Xu R and Adak S. Survival analysis with time-varying regression effects using a tree-based approach. *Biometrics* 2002; 58(2): 305–315. DOI:10.1111/j.0006-341x.2002.00305.x.

[24] Stablein DM, Carter WH and Wampler GL. Survival analysis of drug combinations using a hazards model with time-dependent covariates. *Biometrics* 1980; 36(3): 537. DOI:10.2307/2530223. URL http://www.jstor.org/stable/2530223.

[25] Gustafson P. Flexible bayesian modelling for survival data. *Lifetime Data Analysis* 1998; 4(3): 281–299. DOI:10.1023/a:1009673932333.

[26] Berger U, Schäfer J and Ulm K. Dynamic cox modelling based on fractional polynomials: time-variations in gastric cancer prognosis. *Statistics in medicine* 2003; 22(7): 1163–1180. DOI:10.1002/sim.1411.

[27] Austin PC, Fang J and Lee DS. Using fractional polynomials and restricted cubic splines to model non-proportional hazards or time-varying covariate effects in the cox regression model. *Statistics in medicine* 2022; 41(3): 612–624. DOI:10.1002/sim.9259.

[28] Hess KR. Assessing time-by-covariate interactions in proportional hazards regression models using cubic spline functions. *Statistics in Medicine* 1994; 13(10): 1045–1062. DOI:10.1002/sim.4780131007. URL https://mdanderson.elsevierpure.com/en/publications/assessing-timebycovariate-interactions-in-proportional-hazards-re.

[29] Bolard P, Quantin C, Abrahamowicz M et al. Assessing time-by-covariate interactions in relative survival models using restrictive cubic spline functions. *Journal of cancer epidemiology and prevention* 2002; 7(3): 113–122. URL https://pubmed.ncbi.nlm.nih.gov/12665210/.

[30] Giorgi R, Abrahamowicz M, Quantin C et al. A relative survival regression model using b-spline functions to model non-proportional hazards. *Statistics in medicine* 2003; 22(17): 2767–2784. DOI:10.1002/sim.1484.

[31] Boyd AP, Kittelson JM and Gillen DL. Estimation of treatment effect under non-proportional hazards and conditionally independent censoring. *Statistics in medicine* 2012; 31(28): 3504–3515. DOI:10.1002/sim.5440.

[32] Hattori S and Henmi M. Estimation of treatment effects based on possibly misspecified cox regression. *Lifetime Data Analysis* 2012; 18(4): 408–433. DOI:10.1007/s10985-012-9222-8.

[33] Schemper M, Wakounig S and Heinze G. The estimation of average hazard ratios by weighted cox regression. *Statistics in medicine* 2009; 28(19): 2473–2489. DOI:10.1002/sim.3623.

[34] Lin RS and León LF. Estimation of treatment effects in weighted log-rank tests. *Contemporary clinical trials communications* 2017; 8: 147–155. DOI:10.1016/j.conctc.2017.09.004.

[35] Yang S and Prentice RL. Estimation of the 2-sample hazard ratio function using a semiparametric model. *Biostatistics (Oxford, England)* 2011; 12(2): 354–368. DOI:10.1093/biostatistics/kxq061.

[36] Aalen O, Borgan O and Gjessing H. *Survival and Event History Analysis: A Process Point of View*. New york: Springer, 2008.

[37] Achilonu OJ, Fabian J and Musenge E. Modeling long-term graft survival with time-varying covariate effects: An application to a single kidney transplant centre in johannesburg, south africa. *Frontiers in public health* 2019; 7: 201. DOI:10.3389/fpubh.2019.00201.

[38] Xie X, Strickler HD and Xue X. Additive hazard regression models: an application to the natural history of human papillomavirus. *Computational and mathematical methods in medicine* 2013; 2013: 796270. DOI:10.1155/2013/796270.

[39] Dunson DB and Herring AH. Bayesian model selection and averaging in additive and proportional hazards models. *Lifetime Data Analysis* 2005; 11(2): 213–232. DOI:10.1007/s10985-004-0384-x.

[40] Martinussen T and Pipper CB. Estimation of odds of concordance based on the aalen additive model. *Lifetime Data Analysis* 2013; 19(1): 100–116. DOI:10.1007/s10985-012-9234-4.

[41] Jackson C. *Flexible Parametric Survival and Multi-State Models [R package flexsurv version 2.1]*. Comprehensive R Archive Network (CRAN), 2021. URL https://mran.microsoft.com/web/packages/flexsurv/index.html.

[42] Möst L and Hothorn T. Conditional transformation models for survivor function estimation. *The international journal of biostatistics* 2015; 11(1): 23–50. DOI:10.1515/ijb-2014-0006.

[43] Xu J, Psioda MA and Ibrahim JG. Bayesian design of clinical trials using joint models for longitudinal and time-to-event data. *Biostatistics (Oxford, England)* 2022; 23(2): 591–608. DOI:10.1093/biostatistics/kxaa044.

[44] Yang S and Prentice R. Semiparametric analysis of short-term and long-term hazard ratios with two-sample survival data. *Biometrika* 2005; 92(1): 1–17. URL http://www.jstor.org/stable/20441162.

[45] Yang S and Prentice RL. Assessing potentially time-dependent treatment effect from clinical trials and observational studies for survival data, with applications to the women's health initiative combined hormone therapy trial. *Statistics in medicine* 2015; 34(11): 1801–1817. DOI:10.1002/sim.6453.

[46] Yang S. Improving testing and description of treatment effect in clinical trials with survival outcomes. *Statistics in medicine* 2019; 38(4): 530–544. DOI:10.1002/sim.7676.

[47] Devarajan K and Ebrahimi N. On penalized likelihood estimation for a non-proportional hazards regression model. *Statistics & probability letters* 2013; 83(7): 1703–1710. DOI:10.1016/j.spl.2013.03.007.

[48] Demarqui F. *Yang and Prentice Model with Piecewise Exponential Baseline Distribution [R package YPPE version 1.0.1]*. Comprehensive R Archive Network (CRAN), 2020. URL https://cran.r-project.org/web/packages/YPPE/index.html.

[49] Demarqui F. *Yang and Prentice Model with Baseline Distribution Modeled by Bernstein Polynomials [R package YPBP version 0.0.1]*. Comprehensive R Archive Network (CRAN), 2020. URL https://cran.microsoft.com/snapshot/2022-05-21/web/packages/YPBP/index.html.

[50] Wienke A. *Frailty Models in Survival Analysis*. 1 ed. New York: Chapman and Hall/CRC, 2010.

[51] Balan TA and Putter H. A tutorial on frailty models. *Statistical methods in medical research* 2020; 29(11): 3424–3454. DOI:10.1177/0962280220921889. URL https://pubmed.ncbi.nlm.nih.gov/32466712/.

[52] Choi S and Huang X. A general class of semiparametric transformation frailty models for nonproportional hazards survival data. *Biometrics* 2012; 68(4): 1126–1135. DOI:10.1111/j.1541-0420.2012.01784.x.

[53] Zhang J and Lawson AB. Bayesian parametric accelerated failure time spatial model and its application to prostate cancer. *Journal of applied statistics* 2011; 38(2): 591–603. DOI:10.1080/02664760903521476.

[54] Pennell ML and Dunson DB. Bayesian semiparametric dynamic frailty models for multiple event time data. *Biometrics* 2006; 62(4): 1044–1052. DOI:10.1111/j.1541-0420.2006.00571.x.

[55] Wu J and Wei J. Cancer immunotherapy trial design with random delayed treatment effect and cure rate. *Statistics in medicine* 2022; 41(4): 786–797. DOI:10.1002/sim.9258.

[56] Xu Z, Zhu B and Park Y. Design for immuno-oncology clinical trials enrolling both responders and nonresponders. *Statistics in medicine* 2020; 39(27): 3914–3936. DOI:10.1002/sim.8694.

[57] Hagar Y, Dignam JJ and Dukic V. Flexible modeling of the hazard rate and treatment effects in long-term survival studies. *Statistical methods in medical research* 2017; 26(5): 2455–2480. DOI:10.1177/0962280216688034.

[58] Holford TR. The analysis of rates and of survivorship using log-linear models. *Biometrics* 1980; 36(2): 299. DOI:10.2307/2529982. URL http://www.jstor.org/stable/2529982.

[59] Burke K and MacKenzie G. Multi-parameter regression survival modeling: An alternative to proportional hazards. *Biometrics* 2017; 73(2): 678–686. DOI:10.1111/biom.12625.

[60] Peng D, MacKenzie G and Burke K. A multiparameter regression model for interval-censored survival data. *Statistics in medicine* 2020; 39(14): 1903–1918. DOI:10.1002/sim.8508.

[61] Cox C, Chu H, Schneider MF et al. Parametric survival analysis and taxonomy of hazard functions for the generalized gamma distribution. *Statistics in medicine* 2007; 26(23): 4352–4374. DOI:10.1002/sim.2836. URL https://onlinelibrary.wiley.com/doi/10.1002/sim.2836.

[62] Kalbfleisch JD and Prentice RL. *The statistical analysis of failure time data*. 2 ed. New York: EdWiley, 2002.

[63] Phadnis MA, Wetmore JB and Mayo MS. A clinical trial design using the concept of proportional time using the generalized gamma ratio distribution. *Statistics in medicine* 2017; 36(26): 4121–4140. DOI:10.1002/sim.7421.

[64] Spirko-Burns L and Devarajan K. Supervised dimension reduction for large-scale "omics" data with censored survival outcomes under possible non-proportional hazards. *IEEE/ACM transactions on computational biology and bioinformatics* 2021; 18(5): 2032–2044. DOI:10.1109/TCBB.2020.2965934.

[65] Yoshida M and Matsuyama Y. Interim analysis based on the weighted log-rank test for delayed treatment effects under staggered patient entry. *Journal of biopharmaceutical statistics* 2016; 26(5): 842–858. DOI:10.1080/10543406.2015.1094809.

[66] Hasegawa T. Group sequential monitoring based on the weighted log-rank test statistic with the fleming-harrington class of weights in cancer vaccine studies. *Pharmaceutical statistics* 2016; 15(5): 412–419. DOI:10.1002/pst.1760.

[67] Pennell ML, Whitmore GA and Ting Lee ML. Bayesian random-effects threshold regression with application to survival data with nonproportional hazards. *Biostatistics (Oxford, England)* 2010; 11(1): 111–126. DOI:10.1093/biostatistics/kxp041.

[68] Race JA and Pennell ML. Semi-parametric survival analysis via dirichlet process mixtures of the first hitting time model. *Lifetime Data Analysis* 2021; 27(1): 177–194. DOI:10.1007/s10985-020-09514-0.

[69] Yu Z, Tu W and Lee MLT. A semi-parametric threshold regression analysis of sexually transmitted infections in adolescent women. *Statistics in medicine* 2009; 28(24): 3029–3042. DOI:10.1002/sim.3686.

[70] He X, Whitmore GA, Loo GY et al. A model for time to fracture with a shock stream superimposed on progressive degradation: the study of osteoporotic fractures. *Statistics in medicine* 2015; 34(4): 652–663. DOI:10.1002/sim.6356.

[71] Mandel M, Mercier F, Eckert B et al. Estimating time to disease progression comparing transition models and survival methods–an analysis of multiple sclerosis data. *Biometrics* 2013; 69(1): 225–234. DOI:10.1111/biom.12002.

[72] Nguyen VQ and Gillen DL. Robust inference in discrete hazard models for randomized clinical trials. *Lifetime Data Analysis* 2012; 18(4): 446–469. DOI:10.1007/s10985-012-9224-6.

[73] Wey A, Vock DM, Connett J et al. Estimating restricted mean treatment effects with stacked survival models. *Statistics in medicine* 2016; 35(19): 3319–3332. DOI:10.1002/sim.6929.

[74] Lowsky DJ, Ding Y, Lee DKK et al. A k-nearest neighbors survival probability prediction method. *Statistics in medicine* 2013; 32(12): 2062–2069. DOI:10.1002/sim.5673.

[75] Sparapani RA, Logan BR, McCulloch RE et al. Nonparametric survival analysis using bayesian additive regression trees (bart). *Statistics in medicine* 2016; 35(16): 2741–2753. DOI:10.1002/sim.6893.

[76] Basak P, Linero A, Sinha D et al. Semiparametric analysis of clustered interval-censored survival data using soft bayesian additive regression trees (sbart). *Biometrics* 2021; DOI:10.1111/biom.13478.

[77] Korepanova N, Seibold H, Steffen V et al. Survival forests under test: Impact of the proportional hazards assumption on prognostic and predictive forests for amyotrophic lateral sclerosis survival. *Statistical methods in medical research* 2020; 29(5): 1403–1419. DOI:10.1177/0962280219862586.

[78] Lin J, Li K and Luo S. Functional survival forests for multivariate longitudinal outcomes: Dynamic prediction of alzheimer's disease progression. *Statistical methods in medical research* 2021; 30(1): 99–111. DOI:10.1177/0962280220941532.

[79] Trinh QA, Hoàng T, Dorizzi B et al. The effect of sample size and mlp architecture on bayesian learning for cancer prognosis–a case study. *Studies in health technology and informatics* 2003; 95: 504–509. URL https://pubmed.ncbi.nlm.nih.gov/14664037/.

[80] de Laurentiis M and Ravdin PM. A technique for using neural network analysis to perform survival analysis of censored data. *Cancer Letters* 1994; 77(2-3): 127–138. DOI:10.1016/0304-3835(94)90095-7.

[81] Cheng MY, Qiu P, Tan X et al. Confidence intervals for the first crossing point of two hazard functions. *Lifetime Data Analysis* 2009; 15(4): 441–454. DOI:10.1007/s10985-009-9132-6.

[82] Zhao YQ, Redman MW and LeBlanc ML. Quantifying treatment effects using the personalized chance of longer survival. *Statistics in medicine* 2019; 38(28): 5317–5331. DOI:10.1002/sim.8363.

[83] Latimer NR, Abrams KR, Lambert PC et al. Assessing methods for dealing with treatment switching in clinical trials: A follow-up simulation study. *Statistical Methods in Medical Research* 2018; 27(3): 765–784. DOI:10.1177/0962280216642264. URL https://doi.org/10.1177/0962280216642264. PMID: 27114326.

[84] Zhu H. Likelihood approaches for proportional likelihood ratio model with right-censored data. *Statistics in Medicine* 2014; 33(14): 2467–2479. DOI:10.1002/sim.6105.

[85] Dunkler D, Schemper M and Heinze G. Gene selection in microarray survival studies under possibly non-proportional hazards. *Bioinformatics (Oxford, England)* 2010; 26(6): 784–790. DOI:10.1093/bioinformatics/btq035. URL https://pubmed.ncbi.nlm.nih.gov/20118118/.

[86] de Iorio M, Johnson WO, Müller P et al. Bayesian nonparametric non-proportional hazards survival modeling. *Biometrics* 2009; 65(3): 762–771. DOI:10.1111/j.1541-0420.2008.01166.x. URL https://onlinelibrary.wiley.com/doi/full/10.1111/j.1541-0420.2008.01166.x.

[87] Chen YQ, Zhang X and Zhao LP. Estimating a scale-change effect for time-varying phenotypes in genome-wide association studies. *Journal of applied statistical science* 2010; 18(4): 477–493.

[88] Peto R and Peto J. Asymptotically efficient rank invariant test procedures. *Journal of the Royal Statistical Society Series A (General)* 1972; 135(2): 185. DOI:10.2307/2344317.

[89] Mantel N. Evaluation of survival data and two new rank order statistics arising in its consideration. *Cancer chemotherapy reports* 1966; 50(3): 163–170.

[90] Klein JP and Moeschberger ML. *Survival Analysis: Techniques for Censored and Truncated Data*. Second edition ed. Statistics for Biology and Health, New York, NY: Springer New York, 2003. ISBN 978-0-387-21645-4.

[91] Royston P and Parmar MKB. A simulation study comparing the power of nine tests of the treatment effect in randomized controlled trials with a time-to-event outcome. *Trials* 2020; 21(1): 315. DOI:10.1186/s13063-020-4153-2.

[92] Magirr D and Burman CF. Modestly weighted logrank tests. *Statistics in medicine* 2019; 38(20): 3782–3790. DOI:10.1002/sim.8186.

[93] Yung G and Liu Y. Sample size and power for the weighted log-rank test and kaplan-meier based tests with allowance for nonproportional hazards. *Biometrics* 2020; 76(3): 939–950. DOI:10.1111/biom.13196.

[94] Wei J and Wu J. Cancer immunotherapy trial design with cure rate and delayed treatment effect. *Statistics in medicine* 2020; 39(6): 698–708. DOI:10.1002/sim.8440.

[95] Ye T and Yu M. A robust approach to sample size calculation in cancer immunotherapy trials with delayed treatment effect. *Biometrics* 2018; 74(4): 1292–1300. DOI:10.1111/biom.12916.

[96] van Houwelingen HC, van de Velde CJH and Stijnen T. Interim analysis on survival data: its potential bias and how to repair it. *Statistics in medicine* 2005; 24(18): 2823–2835. DOI:10.1002/sim.2248.

[97] Brummel SS and Gillen DL. Flexibly monitoring group sequential survival trials when testing is based upon a weighted log-rank statistic. *Sequential analysis* 2014; 33(1): 39–59. DOI:10.1080/07474946.2014.856635.

[98] Kundu MG and Sarkar J. On information fraction for fleming-harrington type weighted log-rank tests in a group-sequential clinical trial design. *Statistics in medicine* 2021; 40(10): 2321–2338. DOI:10.1002/sim.8905.

[99] Li B, Su L, Gao J et al. A group sequential design and sample size estimation for an immunotherapy trial with a delayed treatment effect. *Statistical methods in medical research* 2021; 30(3): 904–915. DOI:10.1177/0962280220980780.

[100] Zhang J and Pulkstenis E. Sample size and power of survival trials in group sequential design with delayed treatment effect. *Statistics in Biopharmaceutical Research* 2016; 8(3): 268–275. DOI:10.1080/19466315.2016.1207560.

[101] Magirr D and Jiménez JL. Design and analysis of group-sequential clinical trials based on a modestly weighted log-rank test in anticipation of a delayed separation of survival curves: A practical guidance. *Clinical trials (London, England)* 2022; 19(2): 201–210. DOI:10.1177/17407745211072848.

[102] Uno H, Tian L, Claggett B et al. A versatile test for equality of two survival functions based on weighted differences of kaplan-meier curves. *Statistics in medicine* 2015; 34(28): 3680–3695. DOI:10.1002/sim.6591.

[103] Dormuth I, Liu T, Xu J et al. Which test for crossing survival curves? a user's guideline. *BMC medical research methodology* 2022; 22(1): 34. DOI:10.1186/s12874-022-01520-0.

[104] Horiguchi M, Cronin AM, Takeuchi M et al. A flexible and coherent test/estimation procedure based on restricted mean survival times for censored time-to-event data in randomized clinical trials. *Statistics in medicine* 2018; 37(15): 2307–2320. DOI:10.1002/sim.7661.

[105] Lawrence J, Qiu J, Bai S et al. Difference in restricted mean survival time: Small sample distribution and asymptotic relative efficiency. *Statistics in Biopharmaceutical Research* 2019; 11(1): 61–66. DOI:10.1080/19466315.2018.1527249.

[106] Sun S, Liu G, Lyu T et al. Design considerations in clinical trials with cure rate survival data: A case study in oncology. *Pharmaceutical statistics* 2018; 17(2): 94–104. DOI:10.1002/pst.1840.

[107] Paukner M and Chappell R. Window mean survival time. *Statistics in medicine* 2021; 40(25): 5521–5533. DOI:10.1002/sim.9138.

[108] Tang Y. Complex survival trial design by the product integration method. *Statistics in medicine* 2022; 41(4): 798–814. DOI:10.1002/sim.9256.

[109] Royston P and Parmar MKB. Restricted mean survival time: an alternative to the hazard ratio for the design and analysis of randomized trials with a time-to-event outcome. *BMC medical research methodology* 2013; 13: 152. DOI:10.1186/1471-2288-13-152.

[110] Brückner M, Burger HU and Brannath W. Nonparametric adaptive enrichment designs using categorical surrogate data. *Statistics in medicine* 2018; 37(29): 4507–4524. DOI:10.1002/sim.7936.

[111] Wang J. Sample size re-estimation with the com-nougue method to evaluate treatment effect. *Statistics in Biosciences* 2022; 14(1): 90–103. DOI: 10.1007/s12561-021-09316-4.

[112] Ghosh P, Ristl R, König F et al. Robust group sequential designs for trials with survival endpoints and delayed response. *Biometrical journal Biometrische Zeitschrift* 2022; 64(2): 343–360. DOI:10.1002/bimj.202000169.

[113] Ristl R, Ballarini NM, Götte H et al. Delayed treatment effects, treatment switching and heterogeneous patient populations: How to design and analyze rcts in oncology. *Pharmaceutical statistics* 2021; 20(1): 129–145. DOI:10.1002/pst.2062.

[114] Li B, Su L, Ye Y et al. M&m: A maximum duration design with the maxcombo test for a group sequential trial of an immunotherapy with a random delayed treatment effect. *Statistics in medicine* 2022; 41(4): 815–830. DOI:10.1002/sim.9251.

[115] Wang L, Luo X and Zheng C. A simulation-free group sequential design with max-combo tests in the presence of non-proportional hazards. *Pharmaceutical statistics* 2021; 20(4): 879–897. DOI:10.1002/pst.2116.

[116] Prior TJ. Group sequential monitoring based on the maximum of weighted log-rank statistics with the fleming-harrington class of weights in oncology clinical trials. *Statistical methods in medical research* 2020; 29(12): 3525–3532. DOI:10.1177/0962280220931560.

[117] Royston P and Parmar MKB. Augmenting the logrank test in the design of clinical trials in which non-proportional hazards of the treatment effect may be anticipated. *BMC medical research methodology* 2016; 16: 16. DOI: 10.1186/s12874-016-0110-x.

[118] León LF, Lin R and Anderson KM. On weighted log-rank combination tests and companion cox model estimators. *Statistics in Biosciences* 2020; 12(2): 225–245. DOI:10.1007/s12561-020-09276-1.

[119] Chi Y and Tsai MH. Some versatile tests based on the simultaneous use of weighted logrank and weighted kaplan-meier statistics. *Communications in Statistics: Simulation and Computation* 2001; 30(4): 743–759. DOI: 10.1081/SAC-100107779.

[120] Gorfine M, Schlesinger M and Hsu L. K-sample omnibus non-proportional hazards tests based on right-censored data. *Statistical methods in medical research* 2020; 29(10): 2830–2850. DOI:10.1177/0962280220907355.

[121] Fleming TR, O'Fallon JR, O'Brien PC et al. Modified kolmogorov-smirnov test procedures with application to arbitrarily right-censored data. *Biometrics* 1980; 36(4): 607. DOI:10.2307/2556114.

[122] Gehan EA. A generalized wilcoxon test for comparing arbitrarily singly-censored samples. *Biometrika* 1965; 52(1-2): 203–224. DOI:10.1093/biomet/52.1-2.203.

[123] Brendel M, Janssen A, Mayer CD et al. Weighted logrank permutation tests for randomly right censored life science data. *Scandinavian Journal of Statistics* 2014; 41(3): 742–761. DOI:10.1111/sjos.12059.

[124] Ditzhaus M and Friedrich S. More powerful logrank permutation tests for two-sample survival data. *Journal of Statistical Computation and Simulation* 2020; 90(12): 2209–2227. DOI:10.1080/00949655.2020.1773463.

[125] Ditzhaus M, Genuneit J, Janssen A et al. Casanova: Permutation inference in factorial survival designs. *Biometrics* 2021; DOI:10.1111/biom.13575.

[126] Ditzhaus M and Pauly M. Wild bootstrap logrank tests with broader power functions for testing superiority. *Computational Statistics & Data Analysis* 2019; 136: 1–11. DOI:10.1016/j.csda.2019.02.001.
